# The domain interface method in non-conforming domain decomposition multifield problems

**DOI:** 10.1007/s00466-016-1361-4

**Published:** 2016-12-26

**Authors:** O. Lloberas-Valls, M. Cafiero, J. Cante, A. Ferrer, J. Oliver

**Affiliations:** 1 CIMNE – Centre Internacional de Metodes Numerics en Enginyeria, Campus Nord UPC, Mòdul C-1 101, c/ Jordi Girona 1-3, 08034 Barcelona, Spain; 2 E.T.S d’Enginyers de Camins, Canals i Ports, Technical University of Catalonia (Barcelona Tech), Campus Nord UPC, Mòdul C-1, c/ Jordi Girona 1-3, 08034 Barcelona, Spain; 3 Escola Superior d’Enginyeries Industrial, Aeroespacial i Audiovisual de Terrassa, Technical University of Catalonia (Barcelona Tech), Campus Terrassa UPC, c/ Colom 11, 08222 Terrassa, Spain

**Keywords:** Domain decomposition methods, Non-conforming interface, Weak coupling techniques for non-matching meshes, Mixed formulations

## Abstract

The Domain Interface Method (DIM) is extended in this contribution for the case of mixed fields as encountered in multiphysics problems. The essence of the non-conforming domain decomposition technique consists in a discretization of a fictitious zero-thickness interface as in the original methodology and continuity of the solution fields across the domains is satisfied by incorporating the corresponding Lagrange Multipliers. The multifield DIM inherits the advantages of its irreducible version in the sense that the connections between non-matching meshes, with possible geometrically non-conforming interfaces, is accounted by the automatic Delaunay interface discretization without considering master and slave surfaces or intermediate surface projections as done in many established techniques, e.g. mortar methods. The multifield enhancement identifies the Lagrange multiplier field and incorporates its contribution in the weak variational form accounting for the corresponding consistent stabilization term based on a Nitsche method. This type of constraint enforcement circumvents the appearance of instabilities when the Ladyzhenskaya–Babuška–Brezzi (LBB) condition is not fulfilled by the chosen discretization. The domain decomposition framework is assessed in a large deformation setting for mixed displacement/pressure formulations and coupled thermomechanical problems. The continuity of the mixed field is studied in well selected benchmark problems for both mixed formulations and the objectivity of the response is compared to reference monolithic solutions. Results suggest that the presented strategy shows sufficient potential to be a valuable tool in situations where the evolving physics at particular domains require the use of different spatial discretizations or field interpolations.

## Introduction

The growing demand of industrial complex simulations, together with the continuous improvement of computer technology, have stimulated the popularity of algorithms capable of processing large systems of equations. This is the case of algorithms based on domain decomposition techniques which basically stem from the need of decomposing a large discretization into a number of smaller discretizations, e.g. using finite elements (FEs), or from the assembly of independently generated meshes which form part of a whole system. The latter scenario can be found for instance during simulations of an aircraft where both wings and fuselage discretizations are designed independently. In these situations, the type of algorithms capable of gluing non-matching meshes are crucial and represent the main focus in the present contribution.

Examples of non-conforming interfaces arising from the assembly of non-matching grids can be found in [24, 25] where particular structural components are reused in evolving designs such as wings or rotor blades among diverse aircraft fuselages. The most complex and time-evolving situations for non-conforming interfaces can be definitely found in the field of contact mechanics [16, 28, 31, 41] which has boosted the appearance of a considerable number of strategies to handle non-matching grids. It is also necessary to resort to these type of algorithms when the nature of the physics involved in each of the studied domains is remarkably different, e.g. fluid structure interaction [10] or because the spatial resolution for the adjacent discretizations is significantly different, e.g. multiscale and multiresolution techniques [21, 22].

**Fig. 1:**
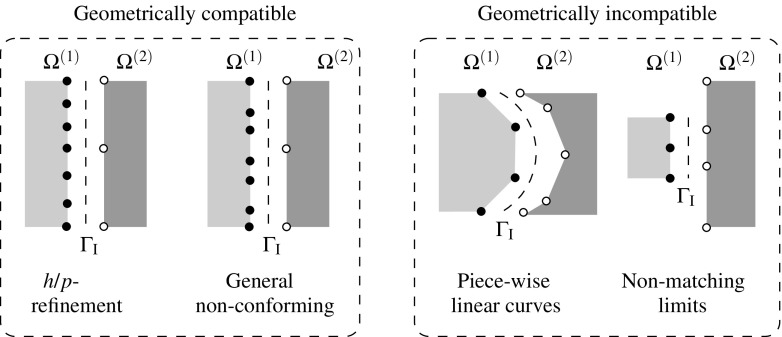
Geometrically compatible (*left*) and geometrically incompatible (*right*) interfaces

The most general scenario at a non-conforming interface $\Gamma _{\text {I}}$ arises when the geometrical compatibility between the non-matching meshes is not satisfied, i.e. when the boundaries of the domains at the common discretized interface are not identical in the undeformed configuration (cf. Fig. 1).

Many Domain Decomposition techniques assume that the interface is geometrically compatible [33] but more general situations may arise from the assembly of independent discretizations and curved interfaces with piece-wise linear distinct discretizations. In such scenarios, powerful techniques are required such as mortar techniques [5, 30–32, 40] or the recently introduced DIM [8].

In mortar methods, the coupling constraints are enforced in a weak sense, i.e. in an integral or average sense along portions of the interface, and are often referred to as segment-to-segment techniques. In general, a displacement field at the interface, ${\mathbf{u}}_{\Gamma _{\text {I}}}$, is taken as reference in order to generate the constraints. A frequently adopted choice is to consider the reference field based on one of the domain discretizations at the common interface.

**Fig. 2:**
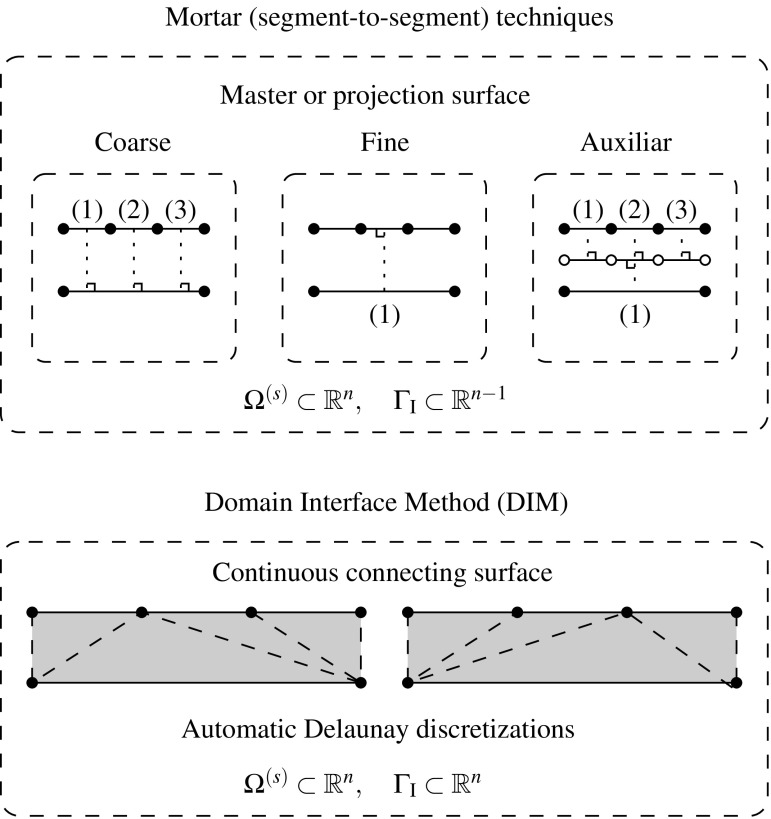
Mortar versus continuous connecting interface strategies

One significant drawback of such classical mortar techniques arises when the coarsest discretization is selected for the reference solution field. In such scenarios, the performance of mortar methods might be less optimal as argued in [8, 18]. More advanced mortar techniques [30–32, 40] consider a more convenient Lagrange multiplier space or even a third auxiliary surface with an optimal node distribution in order to minimize the gap between domains (cf. Fig. 2). This may circumvent these flaws although the associated computational cost due to the extra unknowns may increase as well as the complexity of the resulting algorithm.

**Fig. 3:**
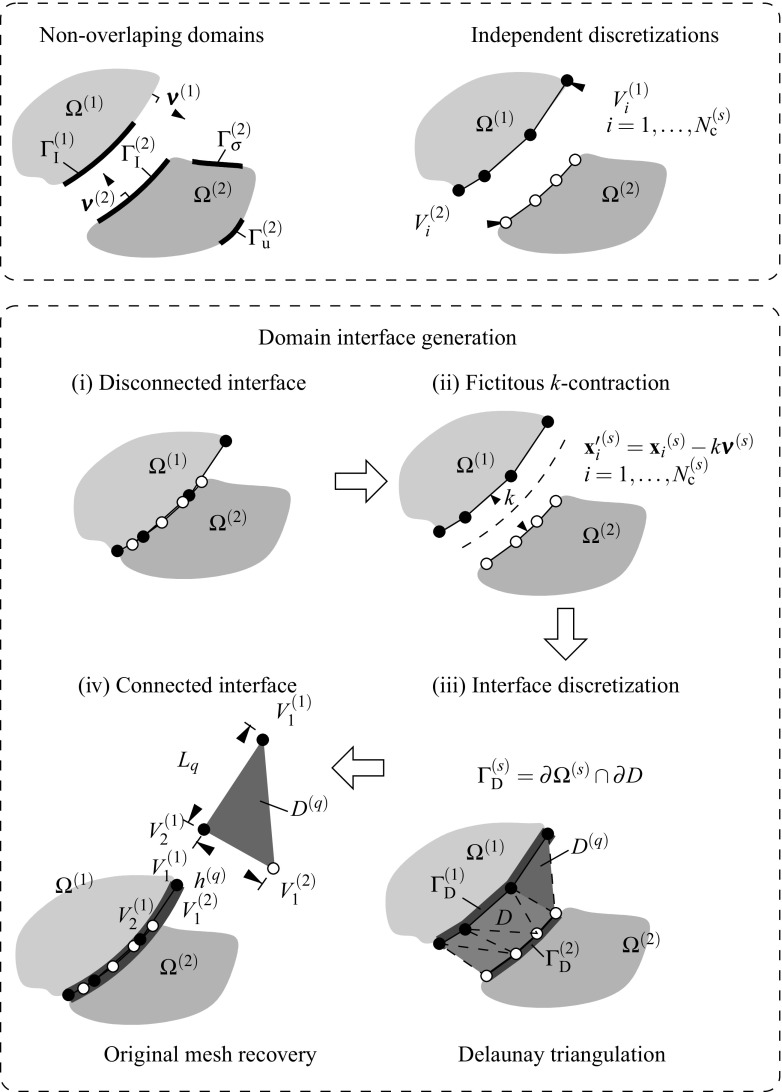
Generation of the domain interface: (*i*) domain discretizations, (*ii*) fictitious domain contractions, (*iii*) Delaunay triangulations and (*iv*) original mesh recovery

In contrast with established mortar methods, the DIM [8] is based on a continuous Delaunay discretization of the interface obtained after a fictitious contraction of the adjacent domains (cf. Figs. 2, 3). This technique has proven to achieve equivalent optimal performances without the drawback of selecting a projection surface since this is automatically accounted for by the Delaunay interface discretization. Moreover, the adjacent interface patch discretizations has been utilized to remove the singularities that arise when the decomposed domain is floating, i.e. exhibits rigid body modes (RBMs). This is performed in a non-intrusive fashion compared to other established dual formulations.

Lagrange multipliers [1] are typically employed to enforce the necessary constraints at general interfaces, e.g. possibly non-conforming. To this end, the term concerning the mechanical work at the non-conforming interface is added to the variational statement. Special care needs to be taken when selecting the shape functions associated to the mesh discretizations for both adjacent domains and the distribution functions of the Lagrange multipliers. In other words, the selection of such functions can not be arbitrary since they need to fulfill the Ladyzhenskaya–Babuška–Brezzi (LBB) condition (also known as the *inf-sup* condition) [2] in order to guarantee that both discretizations converge to the right solution upon mesh refinement. In the current approach a Lagrange multiplier method is adopted to enforce the interface constraints but a Nitsche method [27] is considered to stabilize the system. Such method essentially modifies the weak form by adding a term with a positive parameter that depends on the particular mesh size but does not lead to an ill-conditioned system even for small values of the parameter. A Nitsche method to account for the interface constraints derived from domain decomposition methods has been introduced in [4] and the extra term added at the variational principle basically couples the multipliers with the stress fields at the interface [17]. A main advantage is that the discretization of the hybrid solution field containing the Lagrange multipliers can be performed without any constraints since the stability is accounted for by the extra Nitsche-type term. In the present contribution, this feature is specially valuable since the multifield nature of the problem renders a particularly hybrid solution field. Some generalities of the DIM are revisited in Sect. 2 and the multifield extensions to the method in order to tackle mixed incompressible ${{\mathbf {{u}}}}/p$ and coupled thermomechanical ${{\mathbf {{u}}}}/p/\theta$ problems are detailed in Sects. 3 and 4. The reader that is not familiar with the constitutive model details for the mixed formulations is referred to Appendices 1 and 1 and the references therein for a complete overview. The domain decomposition framework is validated considering benchmark examples for both mixed type formulations in Sect. 6.

## General description

The geometrical aspects of the DIM introduced in [8] are summarized in this section for the case of an irreducible formulation. The concept of geometrical gap and the Lagrange multipliers identification are revisited and the basic notation is introduced.

Finite strain theory is considered in our developments, although an infinitesimal deformation approach can be recovered by considering small displacements (i.e. compared to the domain dimensions) and infinitesimal displacement gradients. Compact notation is utilized for tensor quantities in this contribution.

### Geometrical description of the DIM

Consider the union of two non-overlapping domains $\Omega ^{(1)}$ and $\Omega ^{(2)}$ as depicted in Fig. 3 (top). Dirichlet and Neumann boundary conditions are imposed at each domain $\Omega ^{(s)}$ along disjoint regions depicted by $\Gamma _{\text {u}}^{(s)}$ and $\Gamma _{\sigma }^{(s)}$, respectively. The domain interface $\Gamma _{\text {I}}^{(s)}=\partial \Omega ^{(s)} \cap \partial \Omega ^{(q)}$ with outward unit normal ${\pmb {\nu }}^{(s)}$ where $\partial \Omega$ stands for the domain boundaries of the adjacent domains *s* and *q*.

An independent FE discretization of the two bodies, leads to a number of $N_{\lambda }^{(s)}$ vertices at the domain boundary $\partial \Omega ^{(s)}$ around $\Gamma _{\text {I}}^{(s)}$. These vertices are involved in the interface discretization that characterizes the DIM (cf. step (i) in Fig. 3). The interface generation is summarized in the chart sequence in Fig. 3 and basically starts with a fictitious contraction of the vertices $V_i$ in the direction $-{\pmb {\nu }}^{(s)}$ by a magnitude *k* as indicated in step (ii). As discussed in [8], the magnitude of *k* does not interfere with the results although $k\approx h_{\text {e}}$ is commonly adopted in our analyses where $h_{\text {e}}$ denotes an average equivalent FE size.

A Delaunay triangulation defining the interface domain

1 $$\begin{aligned} D=\underset{q=1}{\overset{N_{\text {q}}}{\bigcup }} D^{(q)} \end{aligned}$$

is obtained based on the resulting fictitious coordinates ${{\mathbf {{x}}}}^{\prime }_i$ of the new vertices. The interface patches are denoted by $D^{(q)}$ and the interface surface $\Gamma _{\text {D}}^{(s)}=\partial \Omega ^{(s)} \cap \partial D$. Note that for the case of a geometrically compatible interface, $\Gamma _{\text {I}}^{(s)}=\Gamma _{\text {D}}^{(s)}$ since the trace of the interface coincides with the interface discretizations of the adjacent domains.

**Fig. 4:**
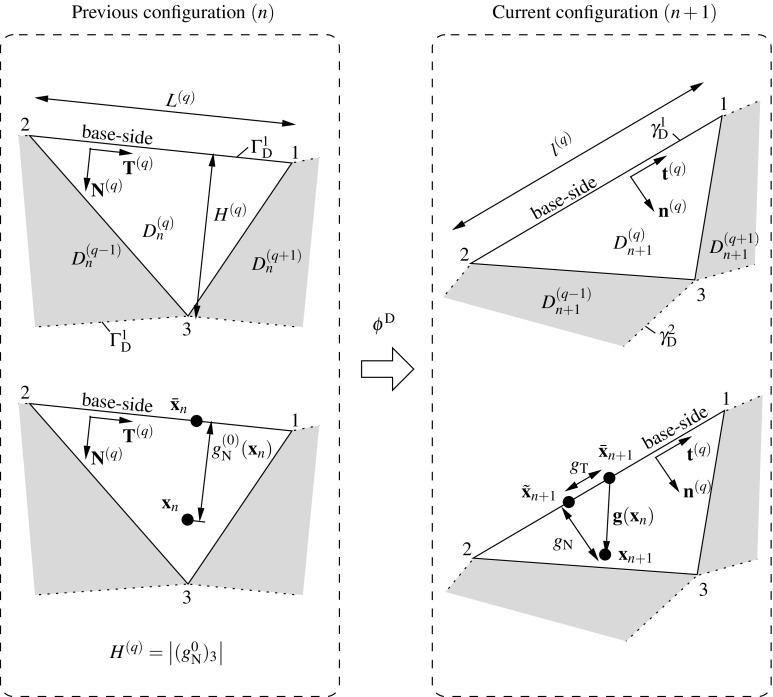
Geometrical definition of the gap ${\mathbf{g}}({\mathbf{x}}_n)$. Previous (*left*) and current (*right*) configurations are related through the incremental motion of the interface domain $\phi ^{\text {D}}$

Triangular linear elements $D^{(q)}$ are considered in our analyses for the discretization of the interface. In this situation, all integrals over a geometrically incompatible interface converge to a bounded value when the distance $h\rightarrow 0$

2 $$\begin{aligned} \displaystyle \sum _{p=1}^{N_{\text {q}}}\lim _{h^{(q)}\rightarrow 0} \int _{D^{(q)}} \dfrac{1}{h^{(q)}}(\bullet )\; \text {d} D= \dfrac{1}{2} \sum _{q=1}^{N_{\text {q}}}\int _{L^{(q)}}(\bullet )\; \text {d} L + \mathcal {O}(h), \end{aligned}$$

Note that the error due to the piece-wise linear approximation of the interface is $\mathcal {O}(h)$. For the case of geometrically compatible interfaces $\mathcal {O}(h)\rightarrow 0$ and

3 $$\begin{aligned} \displaystyle \sum _{q=1}^{N_{\text {p}}}\lim _{h^{(q)}\rightarrow 0} \int _{D^{(q)}} \dfrac{1}{h^{(q)}}(\bullet )\; \text {d} D\approx \dfrac{1}{2} \sum _{q=1}^{N_{\text {q}}}\int _{L^{(q)}}(\bullet )\; \text {d} L. \end{aligned}$$

The main strength of the method is that both adjacent domain interfaces $\Gamma _{\text {D}}^{(s)}$ are automatically taken into account in the construction of the interface links since the integration is performed over $D^{(q)}$ when $h\rightarrow 0$. This represents an obvious advantage from the user point of view considering that no decision has to be made on which interface discretization is selected for the projection of the interface quantities, e.g. in the mortar method [5].

The corresponding deformation map for each domain $\Omega ^{(s)}$ is denoted by $\phi _t^{(s)}({{\mathbf {{X}}}})\equiv \phi ^{(s)}({{\mathbf {{X}}}},t)\; : \; \Omega _{0}^{(s)}\times [0,T]\rightarrow \Omega _{t}^{(s)}$, where material points ${{\mathbf {{X}}}}^{(s)}\in \Omega _{0}^{(s)}$ at the reference configuration are mapped onto the current configuration ${{\mathbf {{x}}}}^{(s)}=\phi _t^{(s)}({{\mathbf {{X}}}})\in \Omega _{t}^{(s)}$. Such current configuration ${{\mathbf {{x}}}}^{(s)}$ can be also obtained in terms of the total displacement field ${{\mathbf {{U}}}}_{t}^{(s)}({{\mathbf {{X}}}}^{(s)})$ as

4 $$\begin{aligned} {{\mathbf {{x}}}}^{(s)}=\phi _t^{(s)}({{\mathbf {{X}}}})={{\mathbf {{X}}}}^{(s)}+{{\mathbf {{U}}}}_{t}^{(s)}({{\mathbf {{X}}}}^{(s)}). \end{aligned}$$

A pseudo-time domain $t\in [0,T]$ is considered in our computations with subdivisions in discrete intervals $[t_n,t_{n+1}]$ of incremental time length $\Delta t=t_{n+1}-t_n$. Configurations at the previous $t_n$ and current $t_{n+1}$ times are denoted as $\Omega _{n}^{(s)}=\phi _{t_n}^{(s)}(\Omega _{0}^{(s)})\equiv \phi _{n}^{(s)}(\Omega _{0}^{(s)})$ and $\Omega _{n+1}^{(s)}=\phi _{t_{n+1}}^{(s)}(\Omega _{0}^{(s)})\equiv \phi _{{n+1}}^{(s)}(\Omega _{0}^{(s)})$, respectively.

As shown in [8], an expression of the incremental motion $\phi ^{(s)}$ can be found by substitution of the current and previous configurations leading to

5 $$\begin{aligned} \phi _{n+1}^{(s)}\left( {(\phi _{n}^{(s)})^{-1}({{\mathbf {{x}}}}_{n}^{(s)})} \right) =\phi ^{(s)}({{\mathbf {{x}}}}_{n}^{(s)})={{\mathbf {{x}}}}_{n+1}^{(s)}. \end{aligned}$$

Considering the incremental motion in (5), the incremental field

6 $$\begin{aligned} {{\mathbf {{u}}}}^{(s)}({{\mathbf {{x}}}}_n^{(s)})=\phi ^{(s)}({{\mathbf {{x}}}}_{n}^{(s)})-{{\mathbf {{x}}}}_{n}^{(s)}={{\mathbf {{x}}}}_{n+1}^{(s)}-{{\mathbf {{x}}}}_{n}^{(s)}, \quad \forall {{\mathbf {{x}}}}_{n}^{(s)}\in \Omega _n^{(s)}. \end{aligned}$$

The incremental motion of the interface domain can be expressed as

7 $$\begin{aligned} \phi ^{\text {D}}({{\mathbf {{x}}}}_n^{(s)})\equiv {{\mathbf {{x}}}}_{n+1}({{\mathbf {{x}}}}_{n})={{\mathbf {{x}}}}_{n}+{{\mathbf {{u}}}}^{\text {D}}({{\mathbf {{x}}}}_{n}), \quad \forall {{\mathbf {{x}}}}_{n}\in D_n^{(q)}, \end{aligned}$$

where $D_n^{(q)}$ denotes the interface domain *D* at time $t_n$. The current and previous domain interfaces are related by $D_{n+1}=\phi ^{\text {D}}(D_{n})$ and $\gamma _{\text {D}}=\phi ^{\text {D}}(\Gamma _{\text {D}})$ sets the link between current and previous interface surfaces, respectively (cf. Fig. 4).

A linear interpolation of the displacement increments ${\hat{{\mathbf {{u}}}}}_i^{\text {D}}$ corresponding to the interface element vertices is employed to compute the incremental displacement field at the interface domain ${{\mathbf {{u}}}}^{\text {D}}$ as

8 $$\begin{aligned} {{\mathbf {{u}}}}^{\text {D}}({{\mathbf {{x}}}}_{n})\equiv {{\mathbf {{u}}}}^{(q)}({{\mathbf {{x}}}}_{n})=\sum _{i=1}^{3}\mathbb {N}^{\text {u,D}}_i({{\mathbf {{x}}}}_{n}){\hat{{\mathbf {{u}}}}}_i^{\text {D}}, \quad \forall {{\mathbf {{x}}}}_{n}\in D_n^{(q)}, \end{aligned}$$

where $\mathbb {N}^{\text {u,D}}_i$ constitute the linear shape functions for three-node triangular finite elements employed to interpolate the interface displacements. The incremental gradient deformation tensor ${{\mathbf {{f}}}}^{\text {D}}$ can be expressed as

9 $$\begin{aligned} {{\mathbf {{f}}}}^{\text {D}}=\bar{\pmb {\nabla }}\left( {\phi ^{\text {D}}({{\mathbf {{x}}}}_{n})} \right) =\dfrac{\partial {{\mathbf {{x}}}}_{n+1}}{\partial {{\mathbf {{x}}}}_{n}}={\mathbf{1}}+ \bar{\pmb {\nabla }}({{\mathbf {{u}}}}^{\text {D}}), \end{aligned}$$

${{\mathbf {{1}}}}$ being the second order unity tensor and ${\bar{\pmb {\nabla }}}$ denoting the material gradient with respect to the reference previous configuration *n*. Due to the linear interpolation of the incremental displacements in (8), the incremental gradient deformation tensor ${{\mathbf {{f}}}}^{\text {D}}$ is constant at every patch $D^{(q)}$. As introduced in [8] and illustrated in Fig. 4, the normal to the base-line of $D^{(q)}$ in the sense of the normal to the adjacent domain ${\pmb {\nu }}^{(s)}$ is denoted by ${\mathbf{N}}^{(q)}$ and ${\mathbf{T}}^{(q)}=\hat{\mathbf{e}}\times {\mathbf{N}}^{(q)}$, where $\hat{\mathbf{e}}$ denotes the out-of-plane unit vector. With these definitions in hand and considering the incremental motion $\phi ^{\text {D}}$, the current tangential and normal unit vectors read

10 $$\begin{aligned} \begin{aligned}&{{\mathbf {{t}}}}^{(q)}=\dfrac{\phi ^{\text {D}}({{\mathbf {{T}}}}^{(q)})}{\left| \left| \phi ^{\text {D}}({{\mathbf {{T}}}}^{(q)})\right| \right| }= \dfrac{{\mathbf{f}}^{(q)}\cdot {{\mathbf {{T}}}}^{(q)}}{\left| \left| {\mathbf{f}}^{(q)}\cdot {{\mathbf {{T}}}}^{(q)}\right| \right| }\\&{{\mathbf {{n}}}}^{(q)}={{\mathbf {{t}}}}^{(q)}\times \hat{\mathbf{e}}. \end{aligned} \end{aligned}$$

It should be stressed that these vectors are constant within every patch (cf. (10)) but discontinuous across the interface patches since their definition depends only on the local base-line for every interface patch.

The initial normal gap $g_{\text {N}}^{0}$ depicted in Fig. 4 is obtained at the previous configuration *n* for a given point ${\mathbf{x}}_n$ and its normal projection to the base-line $\bar{\mathbf{x}}_n$ as

11 $$\begin{aligned} g_{\text {N}}^{0}({\mathbf{x}}_n)=({\mathbf{x}}_n-\bar{\mathbf{x}}_n)\cdot {\mathbf{N}}^{(q)}. \end{aligned}$$

Consequently, the final gap vector

12 $$\begin{aligned} {{\mathbf {{g}}}}({\mathbf{x}}_n)={\mathbf{x}}_{n+1}-\bar{\mathbf{x}}_{n+1}=\phi ^{\text {D}}({\mathbf{x}}_n)-\phi ^{\text {D}}(\bar{\mathbf{x}}_n), \end{aligned}$$

where ${\mathbf{x}}_{n+1}$ and $\bar{\mathbf{x}}_{n+1}$ stand for the convected points ${\mathbf{x}}_{n}$ and $\bar{\mathbf{x}}_{n}$, respectively. Expressing ${{\mathbf {{g}}}}({\mathbf{x}}_n)$ as a sum of its normal and tangential projections onto the current base-side, equation (12) can be rewritten as

13 $$\begin{aligned} {{\mathbf {{g}}}}({\mathbf{x}}_n)=g_{\text {N}}({\mathbf {{x}}}_n){{\mathbf {{n}}}}^{(q)}+\,g_{\text {T}}({\mathbf {{x}}}_n){{\mathbf {{t}}}}^{(q)} \Rightarrow \left\{ \begin{aligned}&g_{\text {N}}({\mathbf {{x}}}_n)={{\mathbf {{g}}}}({\mathbf{x}}_n)\cdot {{\mathbf {{n}}}}^{(q)}\\&g_{\text {T}}({\mathbf {{x}}}_n)={{\mathbf {{g}}}}({\mathbf{x}}_n)\cdot {{\mathbf {{t}}}}^{(q)}. \end{aligned} \right. \end{aligned}$$

**Fig. 5:**
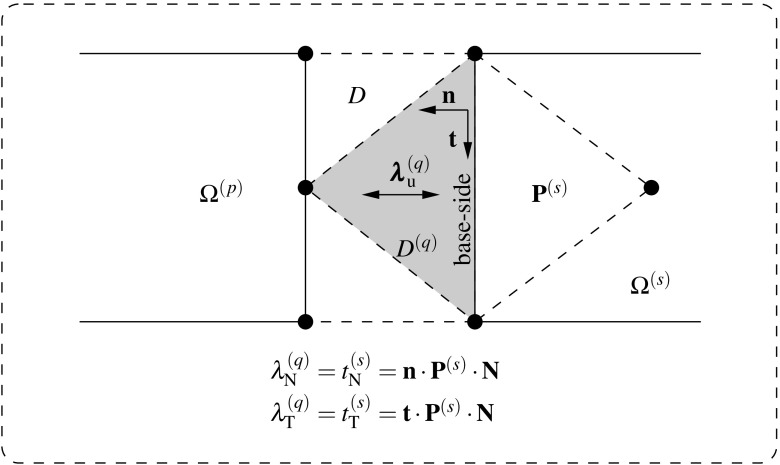
Lagrange multiplier identification at the interface

The normal gap $g_{\text {N}}({\mathbf {{x}}}_n)$ is interpreted as a projection in the direction of ${{\mathbf {{n}}}}^{(q)}$ and, consequently, denotes penetration when $g_{\text {N}}({\mathbf {{x}}}_n)\,{<}\,0$. In the same spirit, the tangential gap $g_{\text {T}}({\mathbf {{x}}}_n)$ stands for the slid distance in the sense of ${{\mathbf {{t}}}}^{(q)}$. The gap is conveniently expressed in terms of the displacement field as detailed in [8]. It essentially considers a Taylor series expansion of $\phi ^{\text {D}}({{\mathbf {{x}}}}_n)$ around $\bar{{\mathbf {{x}}}}_n$ up to second order terms in order to obtain the dependency on the displacements ${{\mathbf {{u}}}}^{(q)}$. A dimensionless measure of the gap, i.e. the gap intensity $\bar{{\mathbf {{g}}}}({\mathbf {{x}}}_n)$ is defined as

14 $$\begin{aligned} \bar{g}_{\text {N}}({\mathbf {{x}}}_n) = \dfrac{{g}_{\text {N}}({\mathbf {{x}}}_n)}{\left| {g}_{\text {N}}^{0}({\mathbf {{x}}}_n)\right| } \quad \text {and} \quad \bar{g}_{\text {T}}({\mathbf {{x}}}_n) = \dfrac{{g}_{\text {T}}({\mathbf {{x}}}_n)}{\left| {g}_{\text {T}}^{0}({\mathbf {{x}}}_n)\right| }. \end{aligned}$$

Finally, one obtains the expression of the gap ready to be utilized in the variational statements as

15 $$\begin{aligned} \begin{aligned} {\bar{g}}_{\text {N}}({\mathbf {{x}}}_n)&=sign\left( {g_{\text {N}}^{0}({\mathbf{x}}_n)} \right) {\mathbf{n}}^{(q)}\cdot \left( {{\mathbf{N}}^{(q)}+\bar{\pmb {\nabla }}({{\mathbf {{u}}}}^{(q)})\cdot {\mathbf{N}}^{(q)}} \right) ,\\ {\bar{g}}_{\text {T}}({\mathbf {{x}}}_n)&=sign\left( {g_{\text {N}}^{0}({\mathbf{x}}_n)} \right) {\mathbf{t}}^{(q)}\cdot \left( {{\mathbf{N}}^{(q)}+\bar{\pmb {\nabla }}({{\mathbf {{u}}}}^{(q)})\cdot {\mathbf{N}}^{(q)}} \right) . \end{aligned} \end{aligned}$$

The interface traction vector ${\mathbf{t}}_{\text {I}}$ defined at the surface $\Gamma _{\text {D}}^{(s)}$ is expressed in terms of the first Piola-Kirchoff stress tensor ${\mathbf{P}}^{(s)}$ at time $n+1$ with respect to the configuration at time *n* and the normal vector ${{\mathbf {{N}}}}^{(q)}={\pmb {\nu }}_{n}^{(s)}$ as

16 $$\begin{aligned} {\mathbf{t}}_{\text {I}}({{\mathbf {{x}}}_n},{{\mathbf {{N}}}})^{(s)}={\mathbf{P}}^{(s)}\cdot {{\mathbf {{N}}}}^{(q)}, \quad \forall {{\mathbf {{x}}}}_n \in \Gamma _{\text {D}}^{(s)}. \end{aligned}$$

The normal and tangential components of ${\mathbf{t}}_{\text {I}}$ are obtained w.r.t. the current normal and tangential vectors as

17 $$\begin{aligned} \left. \begin{aligned} {t}_{\text {I,N}}({\mathbf {{x}}}_n)&={{\mathbf {{n}}}}^{(q)}\cdot {\mathbf{P}}^{(s)}\cdot {{\mathbf {{N}}}}^{(q)}\\ {t}_{\text {I,T}}({\mathbf {{x}}}_n)&={{\mathbf {{t}}}}^{(q)}\cdot {\mathbf{P}}^{(s)}\cdot {{\mathbf {{N}}}}^{(q)} \end{aligned} \right\} \quad \quad \forall {{\mathbf {{x}}}}_n \in \Gamma _{\text {D}}^{(s)} \end{aligned}$$

and are depicted in Fig. 5 inside the surface $\gamma _{\text {D}}^{(s)}$. The normal and tangential components of the interface traction vector implicitly define the Lagrange multipliers that enforce displacement compatibility between domains at each interface patch $D^{(q)}$ as

18 $$\begin{aligned} \left. \begin{aligned} {\mathbf{\lambda }}_{\text {N}}({\mathbf {{x}}}_n)&={t}_{\text {I,N}}({\mathbf {{x}}}_n)\\ {\mathbf{\lambda }}_{\text {T}}({\mathbf {{x}}}_n)&={t}_{\text {I,T}}({\mathbf {{x}}}_n) \end{aligned} \right\} \quad \forall {{\mathbf {{x}}}}_n \in \Gamma _{\text {D}}^{(s)}. \end{aligned}$$

In this scenario, the Lagrange multipliers ${\mathbf{\lambda }}_{\text {N}}$ and ${\mathbf{\lambda }}_{\text {T}}$ are identified as the normal and tangential stresses that connect the adjacent domains (cf. Fig. 5).

#### Remark 2.1

The identification of the Lagrange multipliers ${\mathbf{\lambda }}_{\text {N}}$ and ${\mathbf{\lambda }}_{\text {T}}$ stem from the variational statement corresponding to the weak form of the governing equations

19 $$\begin{aligned} \delta \Pi ^{\text {u}}({{\mathbf {{u}}}},{\pmb {\lambda }}_{\text {I}},\delta {{\mathbf {{u}}}}):= & {} \delta \Pi ^{\text {u}}_{\text {int,ext}}({{\mathbf {{u}}}},\delta {{\mathbf {{u}}}})+ \delta \Pi ^{\text {u}}_{\text {I}}({{\mathbf {{u}}}},{\pmb {\lambda }}_{\text {I}},\delta {{\mathbf {{u}}}})=0,\nonumber \\&\forall \delta {{\mathbf {{u}}}}^{(s)} \in {\hat{{\pmb {\mathcal {V}}}}}_{\text {u}}, \end{aligned}$$

where $\delta {{\mathbf {{u}}}}$ and ${\hat{{\pmb {\mathcal {V}}}}}_{\text {u}}$ denote the displacement variations and their corresponding function space (cf. Appendices 1 and 1 for a more detailed formulation). The energy functionals $\delta \Pi ^{\text {u}}$, $\delta \Pi ^{\text {u}}_{\text {int,ext}}$ and $\delta \Pi ^{\text {u}}_{\text {I}}$ denote the total mechanical work, the mechanical work due to external and internal forces and the mechanical work performed at the interface $\Gamma _{\text {I}}$, respectively, and can be written as:

20 $$\begin{aligned}&\delta \Pi ^{\text {u}}_{\text {int,ext}}({{\mathbf {{u}}}},\delta {{\mathbf {{u}}}})=-\sum _{s=1}^{N_{s}}\left\{ \int _{\Omega _{n}^{(s)}}{{\mathbf {{P}}}}^{(s)}: \bar{\pmb {\nabla }}(\delta {{\mathbf {{u}}}}^{(s)})\;d\Omega \right\} \nonumber \\&\quad +\sum _{s=1}^{N_{\text {s}}}\left\{ \int _{\Gamma _{\sigma }^{(s)}}\hat{{\mathbf {{t}}}}^{(s)}\cdot \delta {{\mathbf {{u}}}}^{(s)}\;d\Gamma \right\} , \end{aligned}$$

21 $$\begin{aligned}&\delta \Pi ^{\text {u}}_{\text {I}}({{\mathbf {{u}}}},{\pmb {\lambda }}_{\text {u}})=\sum _{q=1}^{N_{q}}\left\{ \int _{D_{n}^{(q)}}{\pmb {\lambda }}_{\text {u}}^{(q)}\cdot \delta {\bar{{\mathbf {{g}}}}}^{(q)}({{\mathbf {{u}}}}^{\text {D}})\;dD\right\} , \end{aligned}$$

where $\hat{{\mathbf {{t}}}}$ denote the imposed tractions. The term concerning the interface work $\delta \Pi ^{\text {u}}_{\text {I}}$ can be further elaborated as^1^:

22 $$\begin{aligned} \delta \Pi ^{\text {u}}_{\text {I}}({{\mathbf {{u}}}},{\pmb {\lambda }}_{\text {u}})&=\int _{D_{n}}{\pmb {\lambda }}_{\text {u}}\cdot \delta {\bar{{\mathbf {{g}}}}}({{\mathbf {{u}}}}^{\text {D}})\;dD\nonumber \\&=\int _{D_{n}}{\pmb {\lambda }}_{\text {u}}\cdot \frac{\partial }{\partial {{N}}}\left( {\delta {{\mathbf {{u}}}}^{\text {D}}} \right) \;dD \nonumber \\&=\int _{\partial D_{n}}{\delta {{\mathbf {{u}}}}^{\text {D}}}\cdot {\pmb {\lambda }}_{\text {u}}\;dD-\int _{D_{n}}{\delta {{\mathbf {{u}}}}^{\text {D}}}\cdot \frac{\partial {\pmb {\lambda }}_{\text {u}}}{\partial {{N}}}\;dD\nonumber \\&=\int _{\partial D_{n}}{\delta {{\mathbf {{u}}}}^{\text {D}}}\cdot {\pmb {\lambda }}_{\text {u}}\;dD, \end{aligned}$$

where integration by parts is employed considering the expression of the gap as a function of the displacements in (15) and the constant character of the Lagrange multipliers ${\pmb {\lambda }}_{\text {u}}$. In the limit where the $h^{(q)}$ tends to zero, the expression in (22) can be written as

23 $$\begin{aligned} \delta \Pi ^{\text {u}}_{\text {I}}({{\mathbf {{u}}}},{\pmb {\lambda }}_{\text {u}})= \lim _{h^{(q)}\rightarrow 0} \int _{\partial D_{n}}{\delta {{\mathbf {{u}}}}^{\text {D}}}\cdot {\pmb {\lambda }}_{\text {u}}\;dD = \int _{\Gamma _{\text {I}}}{\delta {{\mathbf {{u}}}}}\cdot {\pmb {\lambda }}_{\text {u}}\;d\Gamma . \end{aligned}$$

The total work in (19) can be rewritten considering that ${\bar{\pmb {\nabla }}}\cdot \left( {{{\mathbf {{P}}}}^{(s)}\cdot \delta {{\mathbf {{u}}}}^{(s)}} \right) =\left( {{\bar{\pmb {\nabla }}}\cdot {{\mathbf {{P}}}}^{(s)}} \right) \cdot {\delta {{\mathbf {{u}}}}^{(s)}}+{{\mathbf {{P}}}}^{(s)}:{\bar{\pmb {\nabla }}}\left( {\delta {{\mathbf {{u}}}}^{(s)}} \right)$ and employing the divergence theorem taking into account all boundary contributions, i.e. $\Gamma = \Gamma _{\sigma } \cup \Gamma _{\text {u}} \cup \Gamma _{\text {I}}$, as

24 $$\begin{aligned} \delta \Pi ^{\text {u}}({{\mathbf {{u}}}},\delta {{\mathbf {{u}}}})=&-\sum _{s=1}^{N_{s}}\left\{ \int _{\Omega _{n}^{(s)}}\left( {{\bar{\pmb {\nabla }}}\cdot {{\mathbf {{P}}}}^{(s)}} \right) \cdot \delta {{\mathbf {{u}}}}^{(s)}\;d\Omega \right\} \nonumber \\&\quad +\sum _{s=1}^{N_{\text {s}}}\left\{ \int _{\Gamma _{\sigma }^{(s)}}\left( {{\hat{{\mathbf {{t}}}}}-{{\mathbf {{P}}}}\cdot {{\mathbf {{N}}}}} \right) \cdot \delta {{\mathbf {{u}}}}^{(s)}\;d\Gamma \right\} \nonumber \\&\quad +\int _{\Gamma _{\text {I}}}\left( {{\pmb {\lambda }}_{\text {u}}-{{\mathbf {{P}}}\cdot {{\mathbf {{N}}}}}} \right) \cdot {\delta {{\mathbf {{u}}}}}\;d\Gamma =0. \end{aligned}$$

Note that the boundary integral over $\Gamma _{\text {u}}$ vanishes according to the definition of the displacement variations $\delta {{\mathbf {{u}}}}$ (cf. (168) in Appendix 1). From the variational principle in (24) one can identify the corresponding Euler-Lagrange equations and natural boundary conditions as

25 $$\begin{aligned}&{\bar{\pmb {\nabla }}}\cdot {{\mathbf {{P}}}}={\mathbf {{0}}}, \qquad \qquad \qquad \qquad \quad \forall {{\mathbf {{x}}}}^{(s)} \in \Omega ^{(s)}, \end{aligned}$$

26 $$\begin{aligned}&{\hat{{\mathbf {{t}}}}}-{{{\mathbf {{P}}}}\cdot {{\mathbf {{N}}}}}={\mathbf {{0}}},\qquad \qquad \qquad \quad \forall {{\mathbf {{x}}}}^{(s)} \in \Gamma _{\sigma }^{(s)}, \end{aligned}$$

27 $$\begin{aligned}&{{\pmb {\lambda }}_{\text {u}}}-{{{\mathbf {{P}}}}\cdot {{\mathbf {{N}}}}}={{\pmb {\lambda }}_{\text {u}}}-{{\mathbf {{t}}}}_{\text {I}}={{\mathbf {{0}}}}, \quad \forall {{\mathbf {{x}}}} \in \Gamma _{\text {I}}, \end{aligned}$$

which concludes the proof that identifies the Lagrange multipliers ${\pmb {\lambda }}_{\text {u}}$ with the tractions ${{\mathbf {{t}}}}_{\text {I}}$ that connect the adjacent domains.

**Fig. 6:**
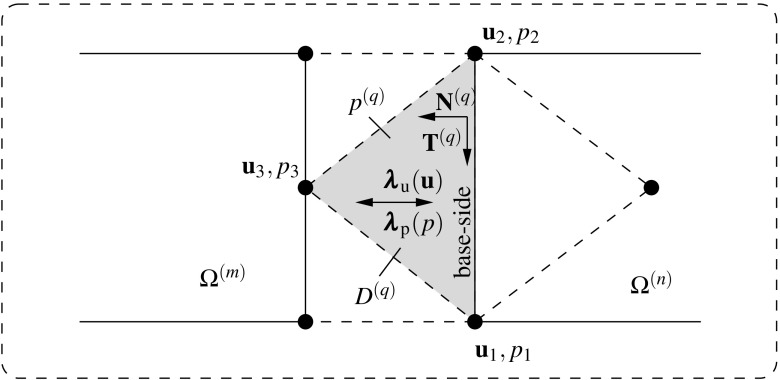
Lagrange multiplier identification at the discretized interface for the mixed ${{\mathbf {{u}}}}/p$ DIM formulation

The strong and weak forms of the irreducible problem employing the DIM are introduced in detail in [8] and skipped in the present approach in order to focus only on the additional formulation content. In this spirit, the following sections are devoted to present a complete variational formulation of the DIM for the mixed ${\mathbf {{u}}}/p$ and thermo-mechanical mixed ${\mathbf {{u}}}/p/\theta$ formulations.

## DIM for mixed ${\mathbf {{u}}}/p$ formulations

The DIM method introduced in the previous sections, and described in detail in [8], is modified in this Section in order to tackle incompressible problems using mixed ${\mathbf {{u}}}/p$ formulations (cf. Appendix 1). The kernel of the extension resides in the identification of the Lagrange multiplier $\lambda _{\text {p}}$ for the correct pressure transference between domains which forces the pressure gap $g_{\text {p}}$ to nullify. Figure 6 illustrates the Lagrange multiplier for both displacement ${\pmb {\lambda }}_{\text {u}}({{\mathbf {{u}}}})$ and pressure $\lambda _{\text {p}}(p)$ fields inside a triangular interface patch $D^{(q)}$ with nodal quantities ${\mathbf {{u}}}_1$ to ${\mathbf {{u}}}_3$ and $p_1$ to $p_3$. The pressure $p^{(q)}$ at the interface patch $D^{(q)}$ is calculated by linearly interpolating the pressure unknowns $p_i^{D}$ as

28 $$\begin{aligned} p^{\text {D}}({{\mathbf {{x}}}}_{n})\equiv {p}^{(q)}({{\mathbf {{x}}}}_{n})=\sum _{i=1}^{3}\mathbb {N}^{\text {p,D}}_i({{\mathbf {{x}}}}_{n})p_i^{\text {D}}, \quad \forall {{\mathbf {{x}}}}_{n}\in D_n^{(q)}. \end{aligned}$$

The geometrical pressure gap $g_{\text {p}}$ is defined for each discretized interface patch $D^{(q)}$ as

29 $$\begin{aligned} g_{\text {p}}=\frac{\partial p^{(q)}}{\partial {N}^{(q)}}=\bar{{\mathbf {{\nabla }}}} p^{(q)}\cdot {{\mathbf {{N}}}}^{(q)} \end{aligned}$$

and is understood as the pressure gradient projected onto the normal to the base side of the triangular patch ${{\mathbf {{N}}}}^{(q)}$. The effective pressure gap ${\bar{g}}_{\text {p}}$ is found normalizing the pressure gap $g_{\text {p}}$ as

30 $$\begin{aligned} {\bar{g}}_{\text {p}}=\frac{g_{\text {p}}}{\left| g_{\text {p}}\right| }. \end{aligned}$$

### Strong and weak forms of the mixed ${\mathbf{u}}/p$ DIM formulation

The strong form of the ${\mathbf{u}}/p$ DIM problem is obtained considering the equations shown in Appendix 1 at each domain $\Omega ^{(s)}$ and accounting for the interface connections between domains as:

31 $$\begin{aligned}&\text {FIND:}\left\{ \begin{aligned}&{{\mathbf {{u}}}}^{(s)}({{\mathbf {{x}}}}^{(s)}_{n}): \quad&\Omega ^{(s)}_{n}\rightarrow \mathbb {R}^{2}, \\&p^{(s)}({{\mathbf {{x}}}}^{(s)}_{n}): \quad&\Omega ^{(s)}_{n}\rightarrow \mathbb {R}, \\&{\pmb {\lambda }}_{\text {u}}({{\mathbf {{x}}}}_{n}): \quad&D_{n}\rightarrow \mathbb {R}^{2}, \\&{\lambda }_{\text {p}}({{\mathbf {{x}}}}_{n}): \quad&D_{n}\rightarrow \mathbb {R}, \end{aligned} \right. \\&\text {FULFILLING:}\nonumber \end{aligned}$$

32 $$\begin{aligned}&\text {Equilibrium equation:} \quad \bar{\pmb {\nabla }}\cdot {{\mathbf {{P}}}}^{(s)}={{\mathbf {{0}}}}, \quad \text {in } \Omega ^{(s)}_{n} \end{aligned}$$

33 $$\begin{aligned}&\text {Constitutive pressure equation:} \quad \frac{p^{(s)}}{\kappa }=\frac{ln(J)^{(s)}}{J^{(s)}}, \quad \text {in } \Omega ^{(s)}_{n} \end{aligned}$$

34 $$\begin{aligned}&\text {Dirichlet's boundary conditions:}\quad {\mathbf{u}}^{(s)}={\hat{\mathbf{u}}^{(s)}}, \quad \text {in } \Gamma ^{(s)}_{\text {u}} \end{aligned}$$

35 $$\begin{aligned}&\text {Neumann's boundary conditions:}\quad {{{\mathbf {{t}}}}^{(s)}} ={{\mathbf {{P}}}}^{(s)}\cdot {{\mathbf {{N}}}}^{(s)}={\hat{\mathbf{t}}^{(s)}}, \text {in } \Gamma ^{(s)}_{\sigma } \end{aligned}$$

36 $$\begin{aligned}&\text {Lagrange multiplier identification:}\quad \left\{ \begin{aligned} \lambda _{\text {N}}&= {t}_{\text {N}}^{(s)}\\ \lambda _{\text {T}}&= {t}_{\text {T}}^{(s)}\\ \lambda _{\text {p}}&=0 \end{aligned} \right. ,\quad \text {in }D_{n}, \end{aligned}$$

37 $$\begin{aligned}&\text {Compatibility constraints:}\quad \left\{ \begin{aligned}&\bar{g}_{\text {N}}({\mathbf {{u}}}^{\text {D}})=0\\&\bar{g}_{\text {T}}({\mathbf {{u}}}^{\text {D}})=0\\&\bar{g}_{\text {p}}(p)=0 \end{aligned} \right. ,\quad \text {in }D_{n}, \end{aligned}$$

${\bar{\pmb {\nabla }}}$ being the material Nabla operator containing derivatives with respect to the previous configuration, ${{\mathbf {{P}}}}$ the first Piola-Kirchhoff stress tensor and ${\hat{\mathbf{t}}}$ and ${\hat{\mathbf{u}}}$ the prescribed tractions and displacements at the corresponding boundaries $\Gamma _{\sigma }$ and $\Gamma _{u}$, respectively. The bulk modulus is denoted by $\kappa$ and $J=\left| \left| \mathbf{f}\right| \right|$ is the determinant of the deformation gradient tensor. A proper justification of the pressure Lagrange multiplier identification in Eq. (36) is provided in Remark 3.2 after the introduction of the weak form of the problem.

As mentioned in Appendix 1, Eq. (32) is solved considering the splitting of the Cauchy stress tensor into the spheric and deviatoric counterparts.

The strong form of the problem stated in (32) to (37) is expressed through the equivalent variational principles. To this end, the solution ${\pmb {\mathcal {V}}}_{\square }$ and weighting $\hat{\pmb {\mathcal {V}}}_{\square }$ spaces for the displacements and pressure fields together with the Lagrange multiplier space ${\pmb {\mathcal {L}}}_{\square }$ for the solution and weighting functions read:

38 $$\begin{aligned}&{\pmb {\mathcal {V}}}_{\text {u}}^{(s)}:=\left\{ {{\mathbf {{u}}}}\Big /{{\mathbf {{u}}}}\Big |_{{\Omega }^{(s)}} \in {\mathcal {H}}^{1}(\Omega ^{(s)}), \quad {\mathbf{u}}^{(s)}={\hat{\mathbf{u}}}^{(s)} \quad \text {in}\quad \Gamma _{\text {u}}^{(s)}\right\} ,\end{aligned}$$

39 $$\begin{aligned}&\hat{\pmb {\mathcal {V}}}_{\text {u}}^{(s)}:=\left\{ \delta {{\mathbf {{u}}}}\Big / \delta {{\mathbf {{u}}}}\Big |_{{\Omega }^{(s)}}\in {\mathcal {H}}^{1}(\Omega ^{(s)}), \quad \delta {\mathbf{u}}^{(s)}={{\mathbf {{0}}}} \quad \text {in}\quad \Gamma _{\text {u}}^{(s)}\right\} ,\end{aligned}$$

40 $$\begin{aligned}&{{\mathcal {V}}}_{\text {p}}^{(s)}:=\left\{ p\Big /p\Big |_{\Omega ^{(s)}} \in {\mathcal {L}}^{2}(\Omega ^{(s)})\right\} ,\end{aligned}$$

41 $$\begin{aligned}&\hat{{\mathcal {V}}}_{\text {p}}^{(s)}:=\left\{ \delta {p}\Big / \delta {p}\Big |_{\Omega }^{(s)}\in {\mathcal {L}}^{2}(\Omega ^{(s)})\right\} ,\end{aligned}$$

42 $$\begin{aligned}&{\pmb {\mathcal {L}}}_{\text {u}}:={{\mathcal {L}}}^2(D),\end{aligned}$$

43 $$\begin{aligned}&{{\mathcal {L}}}_{\text {p}}:={{\mathcal {L}}}^2(D), \end{aligned}$$

${\mathcal {H}}^{1}(\Omega )$ and ${{{L}}}^{2}(\text {D})$ being the Sovolev space of functions with square integrable derivatives and the Lebesgue space of square integrable functions, respectively. The variational statement for the mixed ${{\mathbf {{u}}}}/p$ formulation reads:

44 $$\begin{aligned}&\text {FIND:}\left\{ \begin{aligned}&{{\mathbf {{u}}}}\in {\pmb {\mathcal {V}}}_{\text {u}}^{(s)}: \quad&\Omega _{n}^{(s)}\rightarrow \mathbb {R}^{2},\\&{p}\in {{\mathcal {V}}}_{\text {p}}^{(s)}: \quad&\Omega _{n}^{(s)}\rightarrow \mathbb {R},\\&{\pmb {\lambda }}_{\text {u}}\in {\pmb {\mathcal {L}}}_{\text {u}}: \quad&D_{n}\rightarrow \mathbb {R}^{2},\\&{{\lambda }}_{\text {p}}\in {{\mathcal {L}}}_{\text {p}}: \quad&D_{n}\rightarrow \mathbb {R}, \end{aligned} \right. \\&\text {FULFILLING:}\nonumber \end{aligned}$$

45 $$\begin{aligned}&\delta \Pi ^{\text {u}}({{\mathbf {{u}}}},{\pmb {\lambda }}_{\text {u}},{\delta {{\mathbf {{u}}}}}){=}\delta \Pi ^{\text {u}}_{\text {int,ext}}({{\mathbf {{u}}}},{\delta {{\mathbf {{u}}}}})+\delta \Pi ^{\text {u}}_{\text {I}}({{\mathbf {{u}}}},{\pmb {\lambda }}_{\text {u}},{\delta {{\mathbf {{u}}}}})=0,\nonumber \\&\quad \forall \delta {{\mathbf {{u}}}} \in \hat{\pmb {\mathcal {V}}}_{\text {u}}^{(s)}, \end{aligned}$$

46 $$\begin{aligned}&\delta \Pi ^{\text {p}}(p,\lambda _{\text {p}},\delta {p}){=}\underbrace{\sum _{s=1}^{N_{s}}\left\{ \int _{\Omega _{n}}{\delta p}^{(s)}\left( {\frac{ln(J^{(s)})}{J^{(s)}}-\frac{1}{\kappa }p^{(s)}} \right) \;d\Omega \right\} }_{\delta \Pi ^{\text {p}}_{\Omega }({{\lambda }_{\text {p}}},{\delta {p}})}\nonumber \\&\quad +\delta \Pi ^{\text {p}}_{\text {I}}({{\lambda }_{\text {p}}},{\delta {p}})=0, \quad \forall \delta {p} \in \hat{{\mathcal {V}}}_{\text {p}}^{(s)}, \end{aligned}$$

47 $$\begin{aligned}&\text {AND}\nonumber \\&\delta \Pi _{\lambda _{\text {N}}}({{\mathbf {{u}}}}^{\text {D}},\delta {{\lambda }}_{\text {N}})=0 ,\quad \forall \delta {{\lambda }}_{\text {N}} \in {{\mathcal {L}}}_{\text {u}}, \end{aligned}$$

48 $$\begin{aligned}&\delta \Pi _{\lambda _{\text {T}}}({{\mathbf {{u}}}}^{\text {D}},\delta {{\lambda }}_{\text {T}})=0 ,\quad \forall \delta {{\lambda }}_{\text {T}} \in {{\mathcal {L}}}_{\text {u}}, \end{aligned}$$

49 $$\begin{aligned}&\delta \Pi _{\lambda _{\text {p}}}({p}^{\text {D}},\delta {{\lambda }}_{\text {p}})=0 ,\quad \forall \delta {{\lambda }}_{\text {p}} \in {{\mathcal {L}}}_{\text {p}}. \end{aligned}$$

The part of the mechanical work corresponding to internal and external forces reads

50 $$\begin{aligned} \delta \Pi ^{\text {u}}_{\text {int,ext}}({{\mathbf {{u}}}},\delta {{\mathbf {{u}}}})=\delta \Pi ^{\text {u}}_{\text {int}}({{\mathbf {{u}}}},\delta {{\mathbf {{u}}}})+\delta \Pi ^{\text {u}}_{\text {ext}}(\delta {{\mathbf {{u}}}}) \end{aligned}$$

with

51 $$\begin{aligned} \delta \Pi ^{\text {u}}_{\text {int}}({{\mathbf {{u}}}},\delta {{\mathbf {{u}}}})=-\sum _{s=1}^{N_{s}}\left\{ \int _{\Omega _{n}^{(s)}}{{\mathbf {{P}}}}^{(s)}: \bar{\pmb {\nabla }}(\delta {{\mathbf {{u}}}}^{(s)})\;d\Omega \right\} \end{aligned}$$

and

52 $$\begin{aligned} \delta \Pi ^{\text {u}}_{\text {ext}}(\delta {{\mathbf {{u}}}})=\sum _{s=1}^{N_{\text {s}}}\left\{ \int _{\Gamma _{\sigma }^{(s)}}\hat{{\mathbf {{t}}}}^{(s)}\cdot \delta {{\mathbf {{u}}}}^{(s)}\;d\Gamma \right\} . \end{aligned}$$

The work performed at the interface by the virtual gap reads

53 $$\begin{aligned} \delta \Pi ^{\text {u}}_{\text {I}}({{\mathbf {{u}}}},{\pmb {\lambda }}_{\text {u}})= & {} \int _{D_{n}}{\pmb {\lambda }}_{\text {u}}\cdot \delta {\bar{{\mathbf {{g}}}}}({{\mathbf {{u}}}}^{\text {D}})\;\textit{dD}= \int _{D_{n}}{{\lambda }}_{\text {N}} \delta {\bar{{g}}}_{\text {N}}({{\mathbf {{u}}}}^{\text {D}})\;\textit{dD}\nonumber \\&+\int _{D_{n}}{{\lambda }}_{\text {T}} \delta {\bar{{g}}}_{\text {T}}({{\mathbf {{u}}}}^{\text {D}})\;\textit{dD}, \end{aligned}$$

where $\delta {\bar{{\mathbf {{g}}}}}$ denotes the gap intensity variations. The interface normal and tangential work contributions can also be written in terms of the displacement variations following the expressions of the gap intensity variations developed in [16, 28]. The work performed by the pressure Lagrange multipliers at the interface can be expressed as:

54 $$\begin{aligned} \delta \Pi ^{\text {p}}_{\text {I}}({{\lambda }}_{\text {p}},\delta p)=\int _{D_{n}}{{\lambda }}_{\text {p}} \delta {g}_{\text {p}}^{\text {D}}\;\textit{dD}. \end{aligned}$$

The variational statements for the displacement constraint equations are introduced in detail in [8] and read:

55 $$\begin{aligned} \delta \Pi _{\lambda _\text {N}}({{\mathbf {{u}}}}^{\text {D}},\delta {{\lambda }}_{\text {N}})&= \int _{D_{n}}\delta {{\lambda }}_{\text {N}}{\bar{g}}_{\text {N}}({{\mathbf {{u}}}}^{\text {D}})\;\textit{dD}, \end{aligned}$$

56 $$\begin{aligned} \delta \Pi _{\lambda _\text {T}}({{\mathbf {{u}}}}^{\text {D}},\delta {{\lambda }}_{\text {T}})&= \int _{D_{n}}\delta {{\lambda }}_{\text {T}}{\bar{g}}_{\text {T}}({{\mathbf {{u}}}}^{\text {D}})\;\textit{dD} \end{aligned}$$

and force the interface work to nullify in an average sense along the domain interface $D_n$. Accordingly, the new variational statement for the interface pressure constraint is expressed as:

57 $$\begin{aligned} \delta \Pi _{\lambda _{\text {p}}}(p,\delta {\lambda }_{\text {p}})=\int _{D_{n}}\delta {\lambda }_{\text {p}}g_{\text {p}}^{\text {D}}\;\textit{dD}. \end{aligned}$$

#### Remark 3.1

In order to stabilize the pressure field for the case of compressible and nearly compressible materials the projection term in (183) is added to the weak form of the constitutive equation concerning the incompressibility condition in (46). Such projection term arises from the Polynomial Pressure Projection (PPP) method briefly introduced in Appendix 1.

#### Remark 3.2

The identification of the pressure Lagrange multipliers ${\mathbf{\lambda }}_{\text {p}}$ stem from the variational statement corresponding to the weak form of the governing equations in (46). The expression in (54) concerning the interface work $\delta \Pi ^{\text {p}}_{\text {I}}$ developed by the pressure Lagrange multipliers can be further elaborated as:

58 $$\begin{aligned}&\delta \Pi ^{\text {p}}_{\text {I}}({{\lambda }}_{\text {p}},\delta p)=\int _{D_{n}}{{\lambda }}_{\text {p}} \delta {g}_{\text {p}}^{\text {D}}\;\textit{dD}=\int _{D_{n}}{{\lambda }}_{\text {p}}\frac{\partial }{\partial {N}}\left( {\delta p^{\text {D}}} \right) \;\textit{dD} \nonumber \\&\quad =\int _{\partial D_{n}}\delta p^{\text {D}}\lambda _{\text {p}} \;\textit{dD}-\int _{D_{n}}\delta p^{\text {D}} \frac{\partial \lambda _{\text {p}}}{\partial {{N}}} \;\textit{dD}=\int _{\partial D_{n}}\delta p^{\text {D}} \lambda _{\text {p}} \;\textit{dD}, \end{aligned}$$

where integration by parts is employed considering the expression of the pressure gap in (29) and the constant character of the pressure Lagrange multipliers ${{\lambda }}_{\text {p}}$. In the limit where $h^{(q)}$ tends to zero, the expression in (58) can be written as

59 $$\begin{aligned} \delta \Pi ^{\text {p}}_{\text {I}}({{\lambda }}_{\text {p}},\delta p)=\lim _{h^{(q)}\rightarrow 0} \int _{\partial D_{n}}\delta p^{\text {D}} \lambda _{\text {p}} \;\textit{dD} = \int _{\Gamma _{\text {I}}}\delta p \lambda _{\text {p}} \;d\Gamma . \end{aligned}$$

The total virtual work performed by the pressure variations reads

60 $$\begin{aligned} \delta \Pi ^{\text {p}}(p,\delta {p})= & {} \sum _{s=1}^{N_{s}}\left\{ \int _{\Omega _{n}}{\delta p}^{(s)}\left( {\frac{ln(J^{(s)})}{J^{(s)}}- \frac{1}{\kappa }p^{(s)}} \right) \;d\Omega \right\} \nonumber \\&+\int _{\Gamma _{\text {I}}}\delta p \lambda _{\text {p}} \;d\Gamma =0, \quad \forall \delta {p} \in \hat{{\mathcal {V}}}_{\text {p}}^{(s)}, \end{aligned}$$

and, therefore, the associated Euler-Lagrange equations with the corresponding natural boundary conditions can be identified as:

61 $$\begin{aligned}&\frac{p^{(s)}}{\kappa }=\frac{ln(J)^{(s)}}{J^{(s)}}, \quad \forall {{\mathbf {{x}}}} \in \Omega ^{(s)}, \end{aligned}$$

62 $$\begin{aligned}&\lambda _{\text {p}}=0, \quad \forall {{\mathbf {{x}}}} \in \Gamma _{\text {I}}, \end{aligned}$$

which concludes the proof that identifies the pressure Lagrange multipliers $\lambda _{\text {p}}$ at the interface.

### Discretization using FE and lambda-solvability of the mixed ${{\mathbf {{u}}}}/p$ system

A Galerkin-based spatial discretization is considered in which the solution field components and their variations at each domain $\Omega ^{(s)}$ are interpolated as indicated in (174) to (177) (cf. Appendix 1). The Lagrange multipliers and their variations connecting displacement and pressure fields are interpolated as

63 $$\begin{aligned} {\pmb {\lambda }}_{\text {u}}({{\mathbf {{x}}}}_n)&=\sum _{b} {\Psi }^{\text {u}}_{b}({{\mathbf {{x}}}}_n){\pmb {\Lambda }_{\text {u},b}}\quad \forall {{\mathbf {{x}}}}_n \in D_n\end{aligned}$$

64 $$\begin{aligned} \delta {\pmb {\lambda }}_{\text {u}}({{\mathbf {{x}}}}_n)&=\sum _{b} {\Psi }^{\text {u}}_{b}({{\mathbf {{x}}}}_n)\delta {\pmb {\Lambda }_{\text {u},b}}\quad \forall {{\mathbf {{x}}}}_n \in D_n,\end{aligned}$$

65 $$\begin{aligned} {{\lambda }}_{\text {p}}({{\mathbf {{x}}}}_n)&=\sum _{b} {\Psi }^{\text {p}}_{b}({{\mathbf {{x}}}}_n){{\Lambda }}_{\text {p},b}\quad \forall {{\mathbf {{x}}}}_n \in D_n,\end{aligned}$$

66 $$\begin{aligned} \delta {{\lambda }}_{\text {p}}({{\mathbf {{x}}}}_n)&=\sum _{b} {\Psi }^{\text {p}}_{b}({{\mathbf {{x}}}}_n)\delta {{\Lambda }}_{\text {p},b}\quad \forall {{\mathbf {{x}}}}_n \in D_n, \end{aligned}$$

where the subscript *b* stands for the the discrete nodes corresponding to the Lagrange multipliers interpolation using the domain-wise constant shape functions ${\Psi }^{\text {u}}$ and ${\Psi }^{\text {p}}$ which read:

67 $$\begin{aligned} {\Psi }^{\text {u}}({{\mathbf {{x}}}}_n)=\left\{ \begin{aligned}&{\mathbf {{1}}} \;\forall {{\mathbf {{x}}}}_n \in D_{n}^{(p)}\\&{\mathbf {{0}}} \;\forall {{\mathbf {{x}}}}_n \not \in D_{n}^{(p)} \end{aligned} \right. , \quad {\Psi }^{\text {p}}({{\mathbf {{x}}}}_n)=\left\{ \begin{aligned}&1 \;\forall {{\mathbf {{x}}}}_n \in D_{n}^{(p)}\\&0 \;\forall {{\mathbf {{x}}}}_n \not \in D_{n}^{(p)} \end{aligned} \right. \end{aligned}$$

The corresponding FE approximations of the mixed variational statements in (45) to (49) are denoted as follows:

68 $$\begin{aligned} \delta \Pi ^{\text {u}}({{\mathbf {{u}}}},\delta {{\mathbf {{u}}}},{\pmb {\lambda }}_{\text {u}})\approx \,&\delta \Pi ^{\text {u,h}}({\hat{{\mathbf {{u}}}}},\delta {\hat{{\mathbf {{u}}}}})\nonumber \\ =\,&\delta \Pi ^{\text {u,h}}_{\text {int,ext}}({\hat{{\mathbf {{u}}}}},\delta {\hat{{\mathbf {{u}}}}})+\delta \Pi ^{\text {u,h}}_{\text {I}}({\hat{{\mathbf {{u}}}}},{\pmb {\Lambda }}_{\text {u}},\delta {\hat{{\mathbf {{u}}}}}), \end{aligned}$$

69 $$\begin{aligned} \delta \Pi ^{\text {p}}({{p}},\lambda _{\text {p}},\delta {{p}})\approx \,&\delta \Pi ^{\text {p,h}}({\hat{p}},\Lambda _{\text {p}},\delta {\hat{p}})=\delta \Pi ^{\text {p,h}}_{\Omega }({\hat{p}},\delta {\hat{p}})\nonumber \\&\quad +\delta \Pi _{\text {I}}^{\text {p,h}}(\hat{p},\Lambda _{\text {p}},\delta \hat{p}), \end{aligned}$$

70 $$\begin{aligned} \delta \Pi _{\lambda _\text {N}}({{\mathbf {{u}}}}^{\text {D}},\delta {{\lambda }}_{\text {N}})\approx \,&\delta \Pi ^{\text {h}}_{\lambda _\text {N}}({\hat{{\mathbf {{u}}}}}^{\text {D}},\delta {{\Lambda }}_{\text {N}}), \end{aligned}$$

71 $$\begin{aligned} \delta \Pi _{\lambda _\text {T}}({{\mathbf {{u}}}}^{\text {D}},\delta {{\lambda }}_{\text {T}})\approx \,&\delta \Pi ^{\text {h}}_{\lambda _\text {T}}({\hat{{\mathbf {{u}}}}}^{\text {D}},\delta {{\Lambda }}_{\text {T}}), \end{aligned}$$

72 $$\begin{aligned} \delta \Pi _{\lambda _{\text {p}}}(p^{\text {D}},\delta {\lambda }_{\text {p}})\approx \,&\delta \Pi ^{\text {h}}_{\lambda _{\text {p}}}(\hat{p}^{\text {D}},\delta {\Lambda }_{\text {p}}), \end{aligned}$$

Considering that (68) to (72) hold for any virtual quantity $\delta {\hat{{\mathbf {{u}}}}}$, $\delta {\hat{p}}$, $\delta {{\Lambda }}_{\text {N}}$, $\delta {{\Lambda }}_{\text {T}}$ and $\delta {\Lambda }_{\text {p}}$, the resulting discrete problem consists in finding the nodal quantities ${\hat{{\mathbf {{u}}}}},{\hat{p}},{\pmb {\Lambda }_{\text {u}}}$ and $\Lambda _{\text {p}}$ such that the following residual is nullified:

73 $$\begin{aligned}&{{\mathbf {{R}}}}^{\text {u}}({\hat{{\mathbf {{u}}}}},{\pmb {\Lambda }}_{\text {u}},{\hat{p}}) = {{\mathbf {{R}}}}^{\text {u}}_{\text {int,ext}}({\hat{{\mathbf {{u}}}}},{\hat{p}})+{{\mathbf {{R}}}}^{\text {u}}_{\text {I}}({\hat{{\mathbf {{u}}}}},{\pmb {\Lambda }}_{\text {u}}) ={{\mathbf {{0}}}}, \end{aligned}$$

74 $$\begin{aligned}&{\text {R}}^{\text {p}}(\hat{p},\Lambda _{\text {p}}) = {\text {R}}^{\text {p}}_{\Omega }(\hat{p},\Lambda _{\text {p}})+{{\mathbf {{R}}}}^{\text {p}}_{\text {I}}({\hat{p}},\Lambda _{\text {p}})=0, \end{aligned}$$

75 $$\begin{aligned}&\tilde{{\mathbf {{R}}}}_{\lambda _{\text {u}}}({\hat{{\mathbf {{u}}}}}^{\text {D}}) = \left[ \begin{array}{c} \tilde{R}_{\lambda _\text {N}}({\hat{{\mathbf {{u}}}}}^{\text {D}})\\ \tilde{R}_{\lambda _\text {T}}({\hat{{\mathbf {{u}}}}}^{\text {D}}) \end{array}\right] ={{\mathbf {{0}}}}, \end{aligned}$$

76 $$\begin{aligned}&{\text {R}}_{\lambda _{\text {p}}}({\hat{p}}^{\text {D}})=0. \end{aligned}$$

The residuals concerning the mechanical ${{\mathbf {{R}}}}^{\text {u}}_{\text {int,ext}}$ and incompressible part ${\text {R}}^{\text {p}}$ are detailed in (180) and (181) (cf. Appendix 1). The discrete contributions of the normal and tangential Lagrange multipliers ${{\mathbf {{R}}}}^{\text {u}}_{\text {I}}$ as well as the contributions $\tilde{{\mathbf {{R}}}}_{\lambda _{\text {u}}}$ has been already introduced in [8] for the irreducible formulation. The contribution of the pressure Lagrange multipliers at the interface

77 $$\begin{aligned} {{\mathbf {{R}}}}^{\text {p}}_{\text {I}}({\hat{p}},\Lambda _{\text {p}})=\int _{D_{n}}\left[ \Psi ^{\text {p}}\Lambda _{\text {p}}{\bar{\pmb {\nabla }}}{\mathbb {N}}^{\text {p}}\cdot {{\mathbf {{N}}}}\right] \;\textit{dD} \end{aligned}$$

and the corresponding residual of the pressure Lagrange multiplier restriction

78 $$\begin{aligned} {\text {R}}_{\lambda _{\text {p}}}({\hat{p}}^{\text {D}})=\int _{D_{n}}\left[ {\bar{\pmb {\nabla }}}{\mathbb {N}}^{\text {p}}\cdot {{\mathbf {{N}}}}{\hat{p}}^{\text {D}}\right] \;\textit{dD}. \end{aligned}$$

The discrete equations (73) to (76) are linearized and solved incrementally via a Newton-Raphson procedure leading to the following system:

79 $$\begin{aligned} \left[ \begin{array}{c} {{\mathbf {{R}}}}^{\text {u}}\\ {\text {R}}^{\text {p}}\\ \hline \tilde{{\mathbf {{R}}}}_{\lambda _{\text {u}}}\\ {\text {R}}_{\lambda _{\text {p}}} \end{array} \right] + \left[ \begin{array}{cc|cc} \dfrac{\partial {{\mathbf {{R}}}}^{\text {u}}}{\partial {\hat{{\mathbf {{u}}}}}} &{} \dfrac{\partial {{\mathbf {{R}}}}^{\text {u}}}{\partial {\hat{p}}}&{} {\dfrac{\partial {{\mathbf {{R}}}}^{\text {u}}}{\partial {\pmb {\Lambda }_{\text {u}}}}} &{} 0\\ \dfrac{\partial {\text {R}}^{\text {p}}}{\partial {\hat{{\mathbf {{u}}}}}} &{} \dfrac{\partial {\text {R}}^{\text {p}}}{\partial {\hat{p}}} &{} {{\mathbf {{0}}}}^{\text {T}} &{} \dfrac{\partial {\text {R}}^{\text {p}}}{\partial {\Lambda _{\text {p}}}}\\ \hline \dfrac{\partial \tilde{{\mathbf {{R}}}}_{\lambda _{\text {u}}}}{\partial {\hat{{\mathbf {{u}}}}}} &{} {{0}} &{} \dfrac{\partial \tilde{{\mathbf {{R}}}}_{\lambda _{\text {u}}}}{\partial {\pmb {\Lambda }_{\text {u}}}} &{} 0 \\ {{\mathbf {{0}}}}^{\text {T}} &{} \dfrac{\partial {\text {R}}_{\lambda _{\text {p}}}}{\partial {\hat{p}}} &{} {{\mathbf {{0}}}}^{\text {T}} &{} 0 \end{array} \right] \left[ \begin{array}{c} \Delta {\hat{{\mathbf {{u}}}}}\\ \Delta {\hat{p}}\\ \hline \Delta {\pmb {\Lambda }_{\text {u}}}\\ \Delta {\Lambda _{\text {p}}} \end{array} \right] ={{\mathbf {{0}}}}. \end{aligned}$$

As argued in [8], the component $\dfrac{\partial {\tilde{{\mathbf {{R}}}}}_{\lambda }}{\partial {\pmb {\Lambda }_{\text {u}}}}$ differs from zero due to the introduction of a stabilization term added to the constraint variational statements (70) and (71). Following an analogous procedure, a new stabilization term needs to be added to the variational statement related to the pressure constraint $\delta \Pi ^{\text {p}}_{\lambda _{\text {p}}}$ in order to provide a dependency of the residual component ${{\mathbf {{R}}}}_{\lambda _{\text {p}}}$ with respect to the Lagrange multipliers $\Lambda _{\text {p}}$. The modified residual component

80 $$\begin{aligned} \delta {\tilde{\Pi }}^{\text {p}}_{\lambda _{\text {p}}}(p,\delta {\lambda }_{\text {p}},\delta p)= & {} \delta \Pi ^{\text {p}}_{\lambda _{\text {p}}}(p,\delta {\lambda }_{\text {p}},\delta p)\nonumber \\&+ \int _{\partial D_{n}\cap \Gamma _{\text {D}}^{(s)}}\delta \lambda _{\text {p}}\tau _{\text {p}}\lambda _{\text {p}}\;d\Gamma = 0 ,\quad \forall \delta {{\lambda }}_{\text {p}} \in {{\mathcal {L}}}_{\text {p}}\nonumber \\ \end{aligned}$$

and essentially consists in the addition of a weak format of the pressure Lagrange multiplier identification in (37). It should be noted that, since the penalized term is part of the Euler-Lagrange equations of the variational principle (37), it will tend to zero upon mesh refinement. For this reason the stabilization procedure described in (80) is understood as a consistent penalty method in which, unlike other non-consistent penalty methods, the parameter $\tau _{\text {p}}>0$ can be made significantly small without affecting the quality of the obtained results. Obviously, arbitrarily large values of $\tau _{\text {p}}$ may lead to an ill-conditioning of the resulting system. However, as noted in the example reported in Sect. 6.1, results are remarkably insensitive to the value of $\tau _{\text {p}}$ since a large difference in such values barely affects the resulting pressure field.

The new modified residual

81 $$\begin{aligned} {\tilde{\text {R}}}_{\lambda _{\text {p}}}({\hat{p}}^{\text {D}},\Lambda _{\text {p}})={\text {R}}_{\lambda _{\text {p}}}({\hat{p}}^{\text {D}})+ \int _{\Gamma _n}\tau _{\text {p}}\Psi ^{\text {p}}\Lambda _{\text {p}}\;d\Gamma =0 \end{aligned}$$

and the linearized system in (79) can be rewritten as

82 $$\begin{aligned} \left[ \begin{array}{c} {{\mathbf {{R}}}}^{\text {u}}\\ {\text {R}}^{\text {p}}\\ \hline \tilde{{\mathbf {{R}}}}_{\lambda _{\text {u}}}\\ {\tilde{\text {R}}}_{\lambda _{\text {p}}} \end{array} \right] + \left[ \begin{array}{cc|cc} \dfrac{\partial {{\mathbf {{R}}}}^{\text {u}}}{\partial {\hat{{\mathbf {{u}}}}}} &{} \dfrac{\partial {{\mathbf {{R}}}}^{\text {u}}}{\partial {\hat{p}}}&{} {\dfrac{\partial {{\mathbf {{R}}}}^{\text {u}}}{\partial {\pmb {\Lambda }_{\text {u}}}}} &{} 0\\ \dfrac{\partial {\text {R}}^{\text {p}}}{\partial {\hat{{\mathbf {{u}}}}}} &{} \dfrac{\partial {\text {R}}^{\text {p}}}{\partial {\hat{p}}} &{} {{\mathbf {{0}}}}^{\text {T}} &{} \dfrac{\partial {\text {R}}^{\text {p}}}{\partial {\Lambda _{\text {p}}}}\\ \hline \dfrac{\partial \tilde{{\mathbf {{R}}}}_{\lambda _{\text {u}}}}{\partial {\hat{{\mathbf {{u}}}}}} &{} {{0}} &{} \dfrac{\partial \tilde{{\mathbf {{R}}}}_{\lambda _{\text {u}}}}{\partial {\pmb {\Lambda }_{\text {u}}}} &{} 0 \\ 0 &{} \dfrac{\partial {\tilde{\text {R}}}_{\lambda _{\text {p}}}}{\partial {\hat{p}}} &{} {{\mathbf {{0}}}}^{\text {T}} &{} \dfrac{\partial {\tilde{\text {R}}}_{\lambda _{\text {p}}}}{\partial {\Lambda _{\text {p}}}} \end{array} \right] \left[ \begin{array}{c} \Delta {\hat{{\mathbf {{u}}}}}\\ \Delta {\hat{p}}\\ \hline \Delta {\pmb {\Lambda }_{\text {u}}}\\ \Delta {\Lambda _{\text {p}}} \end{array} \right] ={{\mathbf {{0}}}}. \end{aligned}$$

#### Remark 3.3

Note that the stabilization parameter $\tau _{\text {p}}$ has no dimensions associated with it as opposed to the stabilization parameter $\tau$ utilized for the component ${{\mathbf {{R}}}}_{\lambda }$ and conveniently justified in [8].

The dual assembly in (82) is rewritten considering the discretized quantities for each domain $\Omega {(s)}$ as

83 $$\begin{aligned} \left[ \begin{array}{cccc} {{\mathbf {{K}}}}_{\text {dd}}^{(1)} &{} {\mathbf {{0}}} &{} {\mathbf {{0}}} &{} {{\mathbf {{K}}}}_{\text {d}\Lambda }^{(1)} \\ {\mathbf {{0}}} &{} \ddots &{} {\mathbf {{0}}} &{} \vdots \\ {\mathbf {{0}}} &{} {\mathbf {{0}}} &{} {{\mathbf {{K}}}}_{\text {dd}}^{(N_{\text {s}})}&{} {{\mathbf {{K}}}}_{\text {d}\Lambda }^{(N_{\text {s}})}\\ {{\mathbf {{K}}}}_{\Lambda \text {d}}^{(1)} &{} \dots &{} {{\mathbf {{K}}}}_{\Lambda \text {d}}^{(N_{\text {s}})}&{} {{\mathbf {{K}}}}_{\Lambda \Lambda } \\ \end{array}\right] \left[ \begin{array}{c} \Delta {{\mathbf {{d}}}}^{(1)} \\ \vdots \\ \Delta {{\mathbf {{d}}}}^{(N_{\text {s}})} \\ \Delta \pmb {\Lambda } \end{array}\right] = \left[ \begin{array}{c} {{\mathbf {{r}}}}_{\text {d}}^{(1)} \\ \vdots \\ {{\mathbf {{r}}}}_{\text {d}}^{(N_{\text {s}})}\\ {{\mathbf {{r}}}}_{\Lambda } \end{array}\right] , \end{aligned}$$

where

84 $$\begin{aligned}&\left[ \begin{array}{cc} {{\mathbf {{K}}}}_{\text {dd}}^{(s)} &{} {{\mathbf {{K}}}}_{\text {d}\Lambda }^{(s)} \\ {{\mathbf {{K}}}}_{\Lambda \text {d}}^{(s)} &{} {{\mathbf {{K}}}}_{\Lambda \Lambda } \\ \end{array}\right] = \left[ \begin{array}{cc} \dfrac{\partial \left( {{{\mathbf {{R}}}}_{\text {d}}({{\mathbf {{d}}}},{\pmb {\Lambda }})} \right) ^{(s)}}{\partial {{\mathbf {{d}}}}^{(s)}} &{} \dfrac{\partial \left( {{{\mathbf {{R}}}}_{\text {d}}({{\mathbf {{d}}}},{\pmb {\Lambda }})} \right) ^{(s)}}{\partial {\pmb {\Lambda }}^{(s)}}\\ \dfrac{\partial \left( {{\tilde{{\mathbf {{R}}}}}_{\lambda }({{\mathbf {{d}}}},{\pmb {\Lambda }})} \right) ^{(s)}}{\partial {{\mathbf {{d}}}}^{(s)}} &{} \dfrac{\partial {\tilde{{\mathbf {{R}}}}}_{\lambda }({{\mathbf {{d}}}},{\pmb {\Lambda }})}{\partial {\pmb {\Lambda }}} \end{array}\right] , \end{aligned}$$

85 $$\begin{aligned}&\left[ \begin{array}{c} {{\mathbf {{r}}}}_{\text {d}}^{(s)} \\ {{\mathbf {{r}}}}_{\Lambda } \\ \end{array}\right] = \left[ \begin{array}{c} -\left( {{{\mathbf {{R}}}}_{\text {d}}({{\mathbf {{d}}}},{\pmb {\Lambda }})} \right) ^{(s)}\\ -{\tilde{{\mathbf {{R}}}}}_{\lambda }({{\mathbf {{d}}}},{\pmb {\Lambda }}) \end{array}\right] \end{aligned}$$

and

**Fig. 7:**
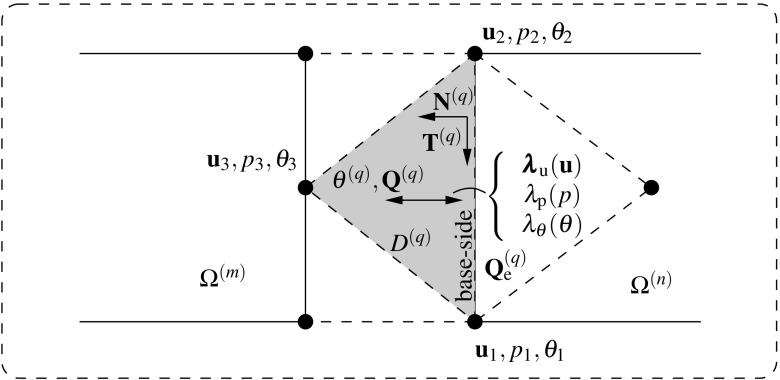
Lagrange multiplier identification at the discretized interface for the coupled thermomechanical ${{\mathbf {{u}}}}/p/\theta$ DIM formulation

86 $$\begin{aligned}&{{\mathbf {{d}}}}^{(s)}=\left[ \begin{array}{c} {\hat{{\mathbf {{u}}}}}^{(s)}\\ {\hat{p}}^{(s)} \end{array}\right] = {{\mathbf {{d}}}}({{\mathbf {{x}}}}_n)|{{\mathbf {{x}}}_n}\in \Omega ^{(s)}, \end{aligned}$$

87 $$\begin{aligned}&{\pmb {\Lambda }}^{(s)}=\left[ \begin{array}{c} {{\pmb {\Lambda }}_{\text {u}}}^{(s)}\\ {\Lambda _{\text {p}}}^{(s)} \end{array}\right] = {{\pmb {\Lambda }}}({{\mathbf {{x}}}}_n)|{{\mathbf {{x}}}_n}\in \Gamma _{\text {I}}^{(s)}=\Gamma _{\text {I}}\cap \Omega ^{(s)}, \end{aligned}$$

88 $$\begin{aligned} \left( {{{\mathbf {{R}}}}_{\text {d}}({{\mathbf {{d}}}},{\pmb {\Lambda }})} \right) ^{(s)}&=\left[ \begin{array}{c} {{{\mathbf {{R}}}}^{\text {u}}}^{(s)}({\mathbf {{d}}},{\pmb {\Lambda }})\\ {{{\mathbf {{R}}}}^{\text {p}}}^{(s)}({\mathbf {{d}}}) \end{array}\right] \nonumber \\&= \tilde{{\mathbf {{R}}}}_{\text {d}}({{\mathbf {{d}}}},{\pmb {\Lambda }}), \forall {{\mathbf {{d}}}}={{\mathbf {{d}}}}^{(s)}\quad \text {and} \quad \forall {\pmb {\Lambda }}={\pmb {\Lambda }}^{(s)}, \end{aligned}$$

89 $$\begin{aligned} \left( {{\tilde{{\mathbf {{R}}}}}_{\lambda }({{\mathbf {{d}}}},{\pmb {\Lambda }})} \right) ^{(s)}&=\left[ \begin{array}{c} {\tilde{{\mathbf {{R}}}}_{\lambda _{\text {u}}}}^{(s)}({\mathbf {{d}}},{\pmb {\Lambda }})\\ {\tilde{\text {R}}}_{\lambda _{\text {p}}}^{(s)}({\mathbf {{d}}},{\pmb {\Lambda }}) \end{array}\right] \nonumber \\&= {\tilde{{\mathbf {{R}}}}}_{\lambda }({{\mathbf {{d}}}},{\pmb {\Lambda }}),\; \forall {{\mathbf {{d}}}}={{\mathbf {{d}}}}^{(s)}\quad \text {and} \quad \forall {\pmb {\Lambda }}={\pmb {\Lambda }}^{(s)}. \end{aligned}$$

Different parallel solution strategies for solving such a system are briefly discussed in Sect. 5 together with an iterative scheme for a general non-linear problem.

## DIM for coupled thermomechanical ${{\mathbf {{u}}}}/p/\theta$ formulations

The coupled thermomechanical formulation utilized in this manuscript is explained in detail in Appendix 1 for the case of a monolithic approach. In this manner, the present section is exclusively focused on the necessary extensions to the DIM in order to tackle such mixed problems. The kernel of the coupled thermomechanical DIM consists in the identification of the appropriate Lagrange multipliers concerning the connection of the displacement, pressure and temperature fields across the interface (cf. Fig. 7). The main addition with respect to the mixed formulation detailed in Sect. 3 is the introduction of the temperature unknowns $\theta$ which are transferred through the corresponding Lagrange multipliers $\lambda _{\theta }$ located at each interface patch $D^{(q)}$. The temperature $\theta ^{(q)}$ at the interface patch $D^{(q)}$ is calculated by linearly interpolating the temperature unknowns $\theta _i^{D}$ as

90 $$\begin{aligned} \theta ^{\text {D}}({{\mathbf {{x}}}}_{n})\equiv {\theta }^{(q)}({{\mathbf {{x}}}}_{n})=\sum _{i=1}^{3}\mathbb {N}^{\theta \text {,D}}_i({{\mathbf {{x}}}}_{n})\theta _i^{\text {D}}, \quad \forall {{\mathbf {{x}}}}_{n}\in D_n^{(q)}. \end{aligned}$$

The heat flux defined at the interface patch $D^{(q)}$ is denoted as ${{\mathbf {{Q}}}}^{(q)}$ and ${{\mathbf {{Q}}}}_{\text {e}}^{(q)}$ refers to the heat flux at the FE adjacent to the base-side of the interface patch. In this manner, the Lagrange multiplier $\lambda _{\theta }$ is defined as the the projection of the adjacent flux ${{\mathbf {{Q}}}}_{\text {e}}^{(q)}$ to the normal ${{\mathbf {{N}}}}^{(q)}$ of the base-line as:

91 $$\begin{aligned} \lambda _{\theta }={{\mathbf {{Q}}}}_{\text {e}}^{(q)}\cdot {{\mathbf {{N}}}}^{(q)}. \end{aligned}$$

The thermal gap $g_{\theta }$ is defined for each discretized interface patch $D^{(q)}$ as

92 $$\begin{aligned} g_{\theta }=\frac{\partial \theta ^{(q)}}{\partial {N}^{(q)}}=\bar{{\mathbf {{\nabla }}}} \theta ^{(q)}\cdot {{\mathbf {{N}}}}^{(q)} \end{aligned}$$

and is understood as the temperature gradient projected onto the normal to the base side of the triangular patch ${{\mathbf {{N}}}}^{(q)}$. In order to provide a continuous heat flux across the interfaces the thermal gap is forced to be null. The effective temperature gap ${\bar{g}}_{\theta }$ is found normalizing the temperature gap $g_{\theta }$ as

93 $$\begin{aligned} {\bar{g}}_{\theta }=\frac{g_{\text {p}}}{\left| g_{\theta }\right| }. \end{aligned}$$

### Strong and weak forms of the coupled thermomechanical ${\mathbf{u}}/p/\theta$ DIM formulation

The strong form of the mixed ${\mathbf{u}}/p/\theta$ formulation introduced in detail in Appendix 1 is considered at each domain $\Omega ^{(s)}$ accounting for the interface restrictions. In this view, the strong form of the DIM thermomechanical formulation can be stated as:

94 $$\begin{aligned}&\text {FIND:}\left\{ \begin{aligned}&{{\mathbf {{u}}}}\in {\pmb {\mathcal {V}}}_{\text {u}}^{(s)}: \quad&\Omega _{n}^{(s)}\rightarrow \mathbb {R}^{2},\\&{p}\in {{\mathcal {V}}}_{\text {p}}^{(s)}: \quad&\Omega _{n}^{(s)}\rightarrow \mathbb {R},\\&\theta \in {{\mathcal {V}}}_{\theta }^{(s)}: \quad&\Omega _{n}^{(s)}\rightarrow \mathbb {R},\\&{\pmb {\lambda }}_{\text {u}}\in {\pmb {\mathcal {L}}}_{\text {u}}: \quad&D_{n}\rightarrow \mathbb {R}^{2},\\&{{\lambda }}_{\text {p}}\in {{\mathcal {L}}}_{\text {p}}: \quad&D_{n}\rightarrow \mathbb {R},\\&{\lambda }_{\theta }\in {{\mathcal {L}}}_{\theta }: \quad&D_{n}\rightarrow \mathbb {R}, \end{aligned} \right. \\&\text {FULFILLING:} \nonumber \end{aligned}$$

95 $$\begin{aligned}&\text {Equilibrium equation:} \quad \bar{\pmb {\nabla }}\cdot {{\mathbf {{P}}}}^{(s)}={{\mathbf {{0}}}}, \quad \text {in } \Omega ^{(s)}_{n} \end{aligned}$$

96 $$\begin{aligned}&\text {Constitutive pressure equation:} \quad \frac{p^{(s)}}{\kappa }=\frac{ln(J)^{(s)}}{J^{(s)}}, \quad \text {in } \Omega ^{(s)}_{n} \end{aligned}$$

97 $$\begin{aligned}&\text {Energy balance:} \quad \rho _0{\dot{U}}^{(s)}+\bar{\pmb {\nabla }}\cdot {{\mathbf {{Q}}}}^{(s)}={D}_{\text {int}}^{(s)}+\rho _0R^{(s)}, \quad \text {in } \Omega ^{(s)}_{n} \end{aligned}$$

98 $$\begin{aligned}&\text {Dirichlet's boundary conditions:}\quad {\mathbf{u}}^{(s)}={\hat{\mathbf{u}}^{(s)}}, \quad \text {in } \Gamma ^{(s)}_{\text {u}} \end{aligned}$$

99 $$\begin{aligned}&\text {Neumann's boundary conditions:}\nonumber \\&\quad \qquad {\mathbf{t}^{(s)}}={{\mathbf {{P}}}}^{(s)}\cdot {{\mathbf {{N}}}}^{(s)}={\hat{\mathbf{t}}^{(s)}}, \quad \text {in } \Gamma ^{(s)}_{\sigma } \end{aligned}$$

100 $$\begin{aligned}&\text {Lagrange multiplier identification:}\nonumber \\&\quad \quad \left\{ \begin{aligned} \lambda _{\text {N}}&= {t}_{\text {N}}^{(s)}\\ \lambda _{\text {T}}&= {t}_{\text {T}}^{(s)}\\ \lambda _{\text {p}}&=0\\ \lambda _{\theta }&={{\mathbf {{Q}}}}_{\text {e}}\cdot {{\mathbf {{N}}}} \end{aligned} \right. ,\quad \text {in }D_{n}, \end{aligned}$$

101 $$\begin{aligned}&\text {Compatibility constraints:}\nonumber \\&\quad \quad \left\{ \begin{aligned}&\bar{g}_{\text {N}}({\mathbf {{u}}}^{\text {D}})=0\\&\bar{g}_{\text {T}}({\mathbf {{u}}}^{\text {D}})=0\\&\bar{g}_{\text {p}}(p)=0\\&\bar{g}_{\theta }(\theta )=0\\ \end{aligned} \right. ,\quad \text {in }D_{n}, \end{aligned}$$

where the domain energy balance equation, the identification of the temperature Lagrange multiplier and the temperature gap restriction are the new ingredients compared to the strong form presented in Sect. 3.

The strong form in (95) to (101) is expressed through the equivalent variational principles in which the weak form presented in Sect. 3 is extended accounting for equations concerning the temperature field.

To this end, the solution ${\pmb {\mathcal {V}}}_{\square }$ and weighting $\hat{\pmb {\mathcal {V}}}_{\square }$ spaces for the displacements, pressure and temperature fields together with the Lagrange multiplier space ${\pmb {\mathcal {L}}}_{\square }$ for the solution and weighting functions read:

102 $$\begin{aligned}&{\pmb {\mathcal {V}}}_{\text {u}}^{(s)}:=\left\{ {{\mathbf {{u}}}}\Big /{{\mathbf {{u}}}}\Big |_{{\Omega }^{(s)}} \in {\mathcal {H}}^{1}(\Omega ^{(s)}), \quad {\mathbf{u}}^{(s)}={\hat{\mathbf{u}}}^{(s)} \quad \text {in}\quad \Gamma _{\text {u}}^{(s)}\right\} , \end{aligned}$$

103 $$\begin{aligned}&\hat{\pmb {\mathcal {V}}}_{\text {u}}^{(s)}:=\left\{ \delta {{\mathbf {{u}}}}\Big / \delta {{\mathbf {{u}}}}\Big |_{{\Omega }^{(s)}}\in {\mathcal {H}}^{1}(\Omega ^{(s)}), \quad \delta {\mathbf{u}}^{(s)}={{\mathbf {{0}}}} \quad \text {in}\quad \Gamma _{\text {u}}^{(s)}\right\} , \end{aligned}$$

104 $$\begin{aligned}&{{\mathcal {V}}}_{\text {p}}^{(s)}:=\left\{ p\Big /p\Big |_{\Omega ^{(s)}} \in {\mathcal {L}}^{2}(\Omega ^{(s)})\right\} , \end{aligned}$$

105 $$\begin{aligned}&\hat{{\mathcal {V}}}_{\text {p}}^{(s)}:=\left\{ \delta {p}\Big / \delta {p}\Big |_{\Omega ^{(s)}}\in {\mathcal {L}}^{2}(\Omega ^{(s)})\right\} , \end{aligned}$$

106 $$\begin{aligned}&{{\mathcal {V}}}_{\theta }^{(s)}:=\left\{ \theta \Big /\theta \Big |_{\Omega ^{(s)}} \in {\mathcal {L}}^{2}(\Omega ^{(s)}), \quad \theta ={\hat{\theta }} \quad \text {in}\quad \Gamma _{\theta }\right\} , \end{aligned}$$

107 $$\begin{aligned}&\hat{{\mathcal {V}}}_{\theta }^{(s)}:=\left\{ \delta {\theta }\Big / \delta {\theta }\Big |_{\Omega ^{(s)}}\in {\mathcal {L}}^{2}(\Omega ^{(s)})\right\} , \end{aligned}$$

108 $$\begin{aligned}&{\pmb {\mathcal {L}}}_{\text {u}}:={{\mathcal {L}}}^2(D), \end{aligned}$$

109 $$\begin{aligned}&{{\mathcal {L}}}_{\text {p}}:={{\mathcal {L}}}^2(D), \end{aligned}$$

110 $$\begin{aligned}&{{\mathcal {L}}}_{\theta }:={{\mathcal {L}}}^2(D), \end{aligned}$$

${\mathcal {H}}^{1}(\Omega )$ and ${{{L}}}^{2}(\text {D})$ being the Sovolev space of functions with square integrable derivatives and the Lebesgue space of square integrable functions, respectively. The variational statement for the mixed ${{\mathbf {{u}}}}/p/\theta$ formulation reads:

111 $$\begin{aligned}&\text {FIND:}\left\{ \begin{aligned}&{{\mathbf {{u}}}}\in {\pmb {\mathcal {V}}}_{\text {u}}^{(s)}: \quad&\Omega _{n}^{(s)}\rightarrow \mathbb {R}^{2},\\&{p}\in {{\mathcal {V}}}_{\text {p}}^{(s)}: \quad&\Omega _{n}^{(s)}\rightarrow \mathbb {R},\\&{\theta }\in {{\mathcal {V}}}_{\theta }^{(s)}: \quad&\Omega _{n}^{(s)}\rightarrow \mathbb {R},\\&{\pmb {\lambda }}_{\text {u}}\in {\pmb {\mathcal {L}}}_{\text {u}}: \quad&D_{n}\rightarrow \mathbb {R}^{2},\\&{{\lambda }}_{\text {p}}\in {{\mathcal {L}}}_{\text {p}}: \quad&D_{n}\rightarrow \mathbb {R},\\&{{\lambda }}_{\theta }\in {{\mathcal {L}}}_{\theta }: \quad&D_{n}\rightarrow \mathbb {R}, \end{aligned} \right. \\&\text {FULFILLING:} \nonumber \end{aligned}$$

112 $$\begin{aligned}&\delta \Pi ^{\text {u}}({{\mathbf {{u}}}},{\pmb {\lambda }}_{\text {u}},{\delta {{\mathbf {{u}}}}})=\delta \Pi _{\text {int,ext}}^{\text {u}}({{\mathbf {{u}}}},\delta {{\mathbf {{u}}}})\nonumber \\&\quad +\delta \Pi ^{\text {u}}_{\text {I}}({{\mathbf {{u}}}},{\pmb {\lambda }}_{\text {u}},{\delta {{\mathbf {{u}}}}})=0, \quad \forall \delta {{\mathbf {{u}}}} \in \hat{\pmb {\mathcal {V}}}_{\text {u}}^{(s)}, \end{aligned}$$

113 $$\begin{aligned}&\delta \Pi ^{\text {p}}(p,{\lambda }_{\text {p}},\delta {p})=\delta \Pi ^{\text {p}}_{\Omega }(p,\delta {p})+\delta \Pi ^{\text {p}}_{\text {I}}({{\lambda }_{\text {p}}},\delta p)\nonumber \\&\quad =0, \quad \forall \delta {p} \in \hat{{\mathcal {V}}}_{\text {p}}^{(s)}, \end{aligned}$$

114 $$\begin{aligned}&\delta \Pi ^{\theta }(\theta ,{\lambda }_{\theta },\delta {\theta })=\delta \Pi _{\text {int,ext}}^{\theta }(\theta , {\delta {\theta }})+\delta \Pi ^{\theta }_{\text {I}}({{\lambda }_{\theta },\delta \theta })=0, \quad \nonumber \\&\quad \qquad \forall \delta {\theta } \in \hat{{\mathcal {V}}}_{\theta }^{(s)}, \end{aligned}$$

115 $$\begin{aligned}&\text {AND}\nonumber \\&\delta \Pi _{\lambda _{\text {N}}}({{\mathbf {{u}}}}^{\text {D}},\delta {{\lambda }}_{\text {N}})=0 ,\quad \forall \delta {{\lambda }}_{\text {N}} \in {{\mathcal {L}}}_{\text {u}}, \end{aligned}$$

116 $$\begin{aligned}&\delta \Pi _{\lambda _{\text {T}}}({{\mathbf {{u}}}}^{\text {D}},\delta {{\lambda }}_{\text {T}})=0 ,\quad \forall \delta {{\lambda }}_{\text {T}} \in {{\mathcal {L}}}_{\text {u}}, \end{aligned}$$

117 $$\begin{aligned}&\delta \Pi _{\lambda _{\text {p}}}({p}^{\text {D}},\delta {{\lambda }}_{\text {p}})=0 ,\quad \forall \delta {{\lambda }}_{\text {p}} \in {{\mathcal {L}}}_{\text {p}}. \end{aligned}$$

118 $$\begin{aligned}&\delta \Pi _{\lambda _{\theta }}(\theta ^{\text {D}},{\delta \lambda }_{\theta })=0 ,\quad \forall \delta {{\lambda }}_{\theta } \in {{\mathcal {L}}}_{\theta }. \end{aligned}$$

The part of the thermal work corresponding to internal and external contributions

119 $$\begin{aligned} \delta \Pi ^{\theta }_{\text {int,ext}}(\theta , {\delta {\theta }})=-\delta \Pi ^{\theta }_{\text {int}}(\theta , {\delta {\theta }})+\delta \Pi ^{\theta }_{\text {ext}}({\delta {\theta }}) \end{aligned}$$

with

120 $$\begin{aligned}&\delta \Pi ^{\theta }_{\text {int}}(\theta , {\delta {\theta }})=\sum _{s=1}^{N_{s}}\left\{ \int _{\Omega _{n}^{(s)}}\delta \theta ^{(s)}c{\dot{\theta }}^{(s)}\;d\Omega \right\} \nonumber \\&\qquad +\left\{ \int _{\Omega _{n}^{(s)}}\left[ k\bar{\pmb {\nabla }}(\delta \theta ^{(s)})\cdot \bar{\pmb {\nabla }}(\theta ^{(s)})-\sqrt{\frac{2}{3}}\Delta \lambda \sigma _{\text {y}}(\theta ^{(s)}) \right] \;d\Omega \right\} \nonumber \\ \end{aligned}$$

and

121 $$\begin{aligned} \delta \Pi ^{\theta }_{\text {ext}}({\delta {\theta }})= & {} \sum _{s=1}^{N_{s}}\left\{ \int _{\Omega _{n}^{(s)}}\delta \theta ^{(s)}{\hat{R}}^{(s)}\;d\Omega \right. \nonumber \\&\left. + \int _{\Gamma _{n}^{(s)}}\delta \theta ^{(s)}\left[ -D^{(s)}\right] \;d\Gamma \right\} , \end{aligned}$$

where ${\hat{R}}^{(s)}$ and $D^{(s)}$ stand for the heat sources and the outward normal heat flux term applied at the interface $\Gamma _n$

The work performed at the interface by the Lagrange multipliers $\lambda _{\theta }$ reads

122 $$\begin{aligned} \delta \Pi ^{\theta }_{\text {I}}({{\lambda }_{\theta },\delta \theta })=\int _{D_{n}}\lambda _{\theta }\delta g_{\theta }(\theta ^{\text {D}})\;\textit{dD}, \end{aligned}$$

where $\delta g_{\theta }$ denotes the thermal gap variations.

#### Remark 4.1

The identification of the temperature Lagrange multipliers ${{\lambda }}_{\theta }$ stem from the variational statement corresponding to the weak form of the governing equations in (114). The expression in (122) concerning the interface work $\delta \Pi ^{\theta }_{\text {I}}$ developed by the temperature Lagrange multipliers can be further elaborated as:

123 $$\begin{aligned} \delta \Pi ^{\theta }_{\text {I}}({{\lambda }}_{\theta },\delta \theta )&=\int _{D_{n}}{{\lambda }}_{\theta } \delta {g}_{\theta } ^{\text {D}}\;\textit{dD}=\int _{D_{n}}{{\lambda }}_{\theta } \frac{\partial }{\partial {N}}\left( {\delta \theta ^{\text {D}}} \right) \;\textit{dD} \nonumber \\&=\int _{\partial D_{n}}\delta \theta ^{\text {D}}\lambda _{\theta } \;\textit{dD}-\int _{D_{n}}\delta \theta ^{\text {D}} \frac{\partial \lambda _{\theta } }{\partial {{N}}} \;\textit{dD}\nonumber \\ {}&=\int _{\partial D_{n}}\delta \theta ^{\text {D}} \lambda _{\theta } \;\textit{dD}, \end{aligned}$$

where integration by parts is employed considering the expression of the temperature gap in (92) and the constant character of the temperature Lagrange multipliers ${{\lambda }}_{\theta }$. In the limit where $h^{(q)}$ tends to zero, the expression in (123) can be written as

124 $$\begin{aligned} \delta \Pi ^{\theta }_{\text {I}}({{\lambda }}_{\theta } ,\delta \theta )=\lim _{h^{(q)}\rightarrow 0} \int _{\partial D_{n}}\delta \theta ^{\text {D}} \lambda _{\theta } \;\textit{dD} = \int _{\Gamma _{\text {I}}}\delta \theta \lambda _{\theta } \;d\Gamma . \end{aligned}$$

The total virtual work performed by the temperature variations reads

125 $$\begin{aligned}&\delta \Pi ^{\theta }(\theta ,\delta \theta )=-\sum _{s=1}^{N_{s}}\left\{ \int _{\Omega _{n}^{(s)}}\delta \theta ^{(s)}c{\dot{\theta }}^{(s)}\;d\Omega \right\} \nonumber \\&\quad -\left\{ \int _{\Omega _{n}^{(s)}}\left[ k\bar{\pmb {\nabla }}(\delta \theta ^{(s)})\cdot \bar{\pmb {\nabla }}(\theta ^{(s)})-\sqrt{\frac{2}{3}}\Delta \lambda \sigma _{\text {y}}(\theta ^{(s)}) \right] \;d\Omega \right\} \nonumber \\&\quad +\sum _{s=1}^{N_{s}}\left\{ \int _{\Omega _{n}^{(s)}}\delta \theta ^{(s)}{\hat{R}}^{(s)}\;d\Omega + \int _{\Gamma _{n}^{(s)}}\delta \theta ^{(s)}\left[ -D^{(s)}\right] \;d\Gamma \right\} \nonumber \\&\quad +\int _{\Gamma _{\text {I}}}\delta \theta \lambda _{\theta } \;d\Gamma =0, \quad \forall \delta \theta \in \hat{{\mathcal {V}}}_{\theta } ^{(s)}, \end{aligned}$$

and, therefore, the associated Euler-Lagrange equations with the corresponding natural boundary conditions can be identified as:

126 $$\begin{aligned}&\rho _0{\dot{U}}^{(s)}+\bar{\pmb {\nabla }}\cdot {{\mathbf {{Q}}}}^{(s)}={D}_{\text {int}}^{(s)}+\rho _0R^{(s)}, \quad \forall {{\mathbf {{x}}}} \in \Omega ^{(s)} \end{aligned}$$

127 $$\begin{aligned}&\lambda _{\theta }=D={{\mathbf {{Q}}}}_{\text {e}}\cdot {{\mathbf {{N}}}}, \quad \forall {{\mathbf {{x}}}} \in \Gamma _{\text {I}}, \end{aligned}$$

which concludes the proof that identifies the pressure Lagrange multipliers $\lambda _{\theta }$ at the interface.

The variational statement for the thermal constraint equations reads:

128 $$\begin{aligned} \delta \Pi _{\lambda _{\theta }}(\theta ^{\text {D}},\delta \lambda _{\theta })=\int _{D_{n}}\delta \lambda _{\theta } g_{\theta }(\theta ^{\text {D}})\;\textit{dD}=0 \end{aligned}$$

and basically enforces in a weak sense the restriction to the thermal gap $g_{\theta }=0$ (cf. equation (118)). In other words, the variational statement in (128) forces overall continuity of the heat flux across the interface.

As argued in [8] and briefly commented in Sect. 3.2, the independence of the restriction statement with respect to the corresponding Lagrange multiplier, e.g. independence of (128) with respect to $\lambda _{\theta }$, leads to a system prone to exhibit instabilities if the adopted solution field discretization does not satisfy the LBB condition [2]. To this end, an extra term is added to the thermal restriction variational statement in order to provide the dependence with $\lambda _{\theta }$ as

129 $$\begin{aligned} \delta \tilde{\Pi }_{\lambda _{\theta }}(\theta ^{\text {D}},\lambda _{\theta },\delta \lambda _{\theta })= \int _{\Gamma _{n}}\delta \lambda _{\theta } \tau _{\theta }\left( {{{\mathbf {{Q}}}(\theta ^{(s)})}\cdot {{\mathbf {{N}}}}{-}\lambda _{\theta }} \right) \;d\Gamma = 0. \end{aligned}$$

The extra term is based in the expression in (100) and enforces the temperature Lagrange multiplier $\lambda _{\theta }$ to be equal to the heat flux vector ${{\mathbf {{Q}}}}$ of the adjacent element projected on to the normal ${{\mathbf {{N}}}}^{(q)}$ of the base-side of the corresponding patch $D^{(q)}$ (cf. Fig. 7).

The stabilization parameter $\tau _{\theta }>0$ is defined as

130 $$\begin{aligned} \tau _{\theta }=\frac{\alpha L}{k}, \end{aligned}$$

$\alpha >0$ being an non-dimensional parameter, *L* stands for the length of the base-side of $D^{(q)}$ and *k* denotes the thermal conductivity coefficient.

### Discretization using FE and lambda-solvability of the thermal system

As seen in the monolithic formulation (cf. Appendix 1), the coupled thermomechanical problem is resolved in a staggered way and, for this reason, only the discretized temperature equations are outlined in this section. The final discretized system needs to be solved together with the mechanical system in (83) outlined in Sect. 3. A full iterative procedure for the solution of the coupled thermomechanical domain decomposition problem is outlined in Sect. 5.

A Galerkin-based spatial discretization is considered in which the solution field components and their variations at each domain $\Omega ^{(s)}$ are interpolated as indicated in (232) to (237) (cf. Appendix 1). The temperature Lagrange multipliers are interpolated as

131 $$\begin{aligned} {{\lambda }}_{\theta }({{\mathbf {{x}}}}_n)&=\sum _{b} {\Psi }^{\theta }_{b}({{\mathbf {{x}}}}_n){{\Lambda }}_{\theta ,b}\qquad \forall {{\mathbf {{x}}}}_n \in D_n,\end{aligned}$$

132 $$\begin{aligned} \delta {{\lambda }}_{\theta }({{\mathbf {{x}}}}_n)&=\sum _{b} {\Psi }^{\theta }_{b}({{\mathbf {{x}}}}_n)\delta {{\Lambda }}_{\theta ,b}\quad \quad \forall {{\mathbf {{x}}}}_n \in D_n, \end{aligned}$$

where the subscript *b* stands for the the discrete nodes corresponding to the Lagrange multipliers interpolation using the shape functions ${\Psi }^{\theta }={\Psi }^{\text {p}}$ defined at each interface patch $D^{(q)}$ as described in (67).

The corresponding FE approximations of the thermal variational statements in (119), (122) and (128) are denoted as follows:

133 $$\begin{aligned}&\delta \Pi ^{\theta }=\delta \Pi _{\text {int,ext}}^{\theta }(\theta , {\delta {\theta }})+\delta \Pi ^{\theta }_{\text {I}}({{\lambda }_{\theta },\delta \theta }) \approx \delta \Pi ^{\theta \text {,h}}(\hat{\theta }, {\delta \hat{\theta }},{\Lambda }_{\theta })\nonumber \\&~~~~~~~~= \, -\delta \Pi ^{\theta }_{\text {int,h}}(\hat{\theta }, {\delta \hat{\theta }})+ \delta \Pi ^{\theta }_{\text {ext,h}}({\delta \hat{\theta }})+ \delta \Pi ^{\theta \text {,h}}_{\text {I}}({{\Lambda }_{\theta },\delta \hat{\theta }}), \end{aligned}$$

134 $$\begin{aligned}&\delta \tilde{\Pi }_{\lambda _{\theta }}(\theta ^{\text {D}},\lambda _{\theta },\delta \lambda _{\theta })\approx \delta \tilde{\Pi }_{\lambda _{\theta }}^{\text {h}}(\theta ^{\text {D}},\Lambda _{\theta },\delta \Lambda _{\theta }). \end{aligned}$$

Considering that (133) and (134) hold for any virtual quantity $\delta {\hat{\theta }}$ and $\delta {{\Lambda }}_{\theta }$, the resulting discrete problem consists in finding the nodal quantities ${\hat{\theta }}$ and $\Lambda _{\theta }$ such that the following residual is nullified:

135 $$\begin{aligned}&{\text {R}}_{\theta }(\hat{\theta },{\Lambda }_{\theta })={\text {R}}^{\theta }_{\text {int,ext}}(\hat{\theta })+ {\text {R}}^{\theta }_{\text {I}}({\Lambda }_{\theta })=0, \end{aligned}$$

136 $$\begin{aligned}&{\text {R}}_{\lambda _{\theta }}(\theta ^{\text {D}},\Lambda _{\theta })=0, \end{aligned}$$

where

137 $$\begin{aligned}&{\text {R}}^{\theta }_{\text {int}}(\hat{\theta })=\int _{\Omega _{n}^{(s)}}\left[ {\mathbb {N}}^{\theta }[c\dot{\hat{\theta }}^{(s)}]+{{\mathbf {{B}}}}^{\text {T}}[k\bar{\pmb {\nabla }}{\hat{\theta }}^{(s)}]\right. \nonumber \\&\quad ~~~~~~~~~~~~~\left. -{{\mathbb {N}}^{\theta }}\left[ \sqrt{\frac{2}{3}}\sigma _{\text {y}}(\hat{\theta }^{(s)})\Delta \lambda \right] \right] \;d\Omega , \end{aligned}$$

138 $$\begin{aligned}&{\text {R}}^{\theta }_{\text {ext}}=\int _{\Omega _{n}^{(s)}}{{\mathbb {N}}^{\theta }}\hat{R}^{(s)}\; d\Omega - \int _{\Gamma _{n}^{(s)}}{{\mathbb {N}}^{\theta }}D^{(s)}\; d\Gamma , \end{aligned}$$

139 $$\begin{aligned}&{\text {R}}^{\theta ,h}_{\text {I}}({\Lambda }_{\theta })=\int _{D_{n}}\left[ \Psi \Lambda _{\theta }\left( {\bar{\pmb {\nabla }}({{\mathbb {N}}^{\theta }})\cdot {{\mathbf {{N}}}}^{(s)}} \right) \right] \; \textit{dD}, \end{aligned}$$

140 $$\begin{aligned}&{\text {R}}_{\lambda _{\theta }}(\theta ^{\text {D}},\Lambda _{\theta })=\int _{D_{n}}\left[ \bar{\pmb {\nabla }}{{\mathbb {N}}^{\theta }}\cdot {{\mathbf {{N}}}}^{(s)} \right] \theta ^{\text {D}}\;\textit{dD}\nonumber \\&~~~~~~~~~~~~~~~~~~~~~~~~ -\int _{\Gamma _{n}}\tau _{\theta }\left[ k{{\mathbf {{N}}}}^{(s)}\cdot \bar{\pmb {\nabla }}{{\mathbb {N}}^{\theta }}\hat{\theta }^{(s)}+\Psi \Lambda _{\theta }\right] \;d\Gamma , \end{aligned}$$

The discrete equations (135) and (136) are linearized and solved incrementally via a Newton-Raphson procedure leading to the following system:

141 $$\begin{aligned} \left[ \begin{array}{c} {\text {R}}^{\theta }\\ {\text {R}}_{\lambda _{\theta }} \end{array} \right] + \left[ \begin{array}{cc} \dfrac{\partial {{\text {R}}^{\theta }}}{\partial \hat{\theta }} &{} \dfrac{\partial {{\text {R}}^{\theta }}}{\partial {\Lambda _{\theta }}}\\ \dfrac{\partial {{\text {R}}_{\lambda _{\theta }}}}{\partial {\hat{\theta }}} &{} \dfrac{\partial {{\text {R}}_{\lambda _{\theta }}}}{\partial {\Lambda _{\theta }}} \end{array} \right] \left[ \begin{array}{c} \Delta {\hat{\theta }}\\ \Delta {\Lambda _{\theta }} \end{array} \right] ={{\mathbf {{0}}}}. \end{aligned}$$

Assembling the contributions for each domain $\Omega ^{(s)}$ the global system of equations reads:

142 $$\begin{aligned} \left[ \begin{array}{cccc} {{K}}_{\theta \theta }^{(1)} &{} {0} &{} {\mathbf {{0}}} &{} {{K}}_{\theta \Lambda _{\theta }}^{(1)} \\ {0} &{} \ddots &{} {0} &{} \vdots \\ {0} &{} {0} &{} {{K}}_{\theta \theta }^{(N_{\text {s}})}&{} {{K}}_{\theta \Lambda _{\theta }}^{(N_{\text {s}})}\\ {{K}}_{\Lambda _{\theta }\theta }^{(1)} &{} \dots &{} {{K}}_{\Lambda _{\theta }\theta }^{(N_{\text {s}})}&{} {{K}}_{\Lambda _{\theta }\Lambda _{\theta }} \\ \end{array}\right] \left[ \begin{array}{c} \Delta {\hat{\theta }}^{(1)} \\ \vdots \\ \Delta {\hat{\theta }}^{(N_{\text {s}})} \\ \Delta \Lambda _{\theta } \end{array}\right] = \left[ \begin{array}{c} {{r}}_{\text {d}}^{(1)} \\ \vdots \\ {{r}}_{\theta }^{(N_{\text {s}})}\\ {{r}}_{\Lambda _{\theta }} \end{array}\right] , \end{aligned}$$

where

143 $$\begin{aligned} \left[ \begin{array}{cc} {{K}}_{\theta \theta }^{(s)} &{} {{K}}_{\theta \Lambda _{\theta }}^{(s)} \\ {{K}}_{\Lambda _{\theta }\theta }^{(s)} &{} {{K}}_{\Lambda _{\theta }\Lambda _{\theta }} \\ \end{array}\right]&= \left[ \begin{array}{cc} \dfrac{\partial {{\text {R}}^{{\theta }^{(s)}}}}{\partial \hat{\theta }^{(s)}} &{} \dfrac{\partial {{\text {R}}^{{\theta }^{(s)}}}}{\partial {\Lambda _{\theta }}^{(s)}}\\ \dfrac{\partial {{\text {R}}_{\lambda _{\theta }}^{(s)}}}{\partial {\hat{\theta }^{(s)}}} &{} \dfrac{\partial {{\text {R}}_{\lambda _{\theta }}}}{\partial {\Lambda _{\theta }}} \end{array}\right] , \end{aligned}$$

144 $$\begin{aligned} \left[ \begin{array}{c} {{r}}_{\theta }^{(s)} \\ {{r}}_{\Lambda _{\theta }} \\ \end{array}\right]&= \left[ \begin{array}{c} -{{\text {R}}^{\theta }}^{(s)}\\ -{\text {R}}_{\lambda _{\theta }} \end{array}\right] \end{aligned}$$

and

145 $$\begin{aligned}&{\hat{\theta }}^{(s)}={\hat{\theta }}({{\mathbf {{x}}}}_n)|{{\mathbf {{x}}}_n}\in \Omega ^{(s)}, \end{aligned}$$

146 $$\begin{aligned}&{{\Lambda }_{\theta }}^{(s)}={{{\Lambda }_{\theta }}}({{\mathbf {{x}}}}_n)|{{\mathbf {{x}}}_n}\in \Gamma _{\text {I}}^{(s)}=\Gamma _{\text {I}}\cap \Omega ^{(s)}. \end{aligned}$$

Different parallel solution strategies for solving such a system are briefly discussed in Sect. 5 together with an iterative scheme for the general non-linear thermomechanical problem.

**Fig. 8:**
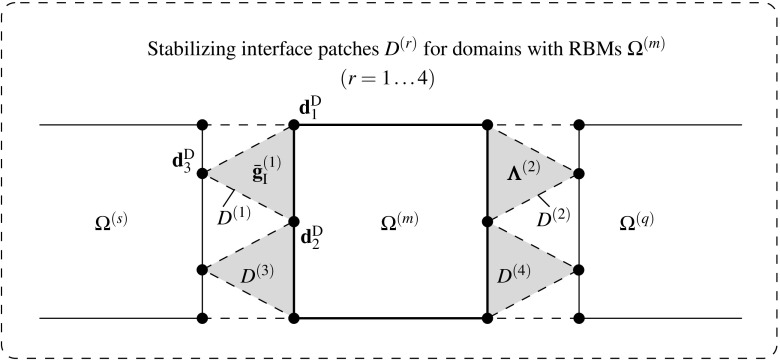
Handling domains $\Omega ^{(m)}$ with RBMs. Stabilizing interface patches $D^{(r)},\; r=1\dots 4$ correspond to the shadowed elements

## Parallel system solution strategies and non-linear iterative scheme

Both mechanical (83) and thermal systems (142) are expected to be large and suitable to be solved using parallel solvers. As argued in [8], both sparse systems can be resolved in a robust manner using direct parallel solvers such as multi-frontal or block-LU methods [34]. Considering that multifield problems may lead to ill conditioned systems, i.e. due to the contrast between stiffness coefficients, this choice might be a suitable one since they are based on independent simultaneous factorizations of the domain matrices and specific, i.e. problem dependent, preconditioners are not required. However, a considerable amount of memory is required and scalability is only expected in a moderate number of processors. As pointed out in [12, 23], block-LU solvers account for automatic load-balancing and multi-threading which is optimal when dealing with highly heterogeneous domains in terms of the number of DOF.

In order to obtain scalabilities in a massive number of processors it is convenient to recast the global systems (83) or (142) in terms of the interface flexibility problem^2^

147 $$\begin{aligned}&{{\mathbf {{F}}}}_{\text {I}}\Delta {\pmb {\Lambda }}={\Delta }{{\mathbf {{g}}}}_{\text {I}}, \end{aligned}$$

148 $$\begin{aligned}&{{\mathbf {{F}}}}_{\text {I}}={{\mathbf {{K}}}}_{\Lambda \Lambda }-{\sum _{s=1}^{N_{\text {s}}}}\left( {{{\mathbf {{K}}}}_{\Lambda \text {d}}^{(s)}\left( {{{\mathbf {{K}}}}_{\text {dd}}^{(s)}} \right) ^{-1}{{\mathbf {{K}}}}_{\text {d}\Lambda }^{(s)}} \right) , \end{aligned}$$

149 $$\begin{aligned}&{\Delta }{{\mathbf {{g}}}}_{\text {I}}={{\mathbf {{r}}}}_{\Lambda }-{\sum _{s=1}^{N_{\text {s}}}}\left( {{{\mathbf {{K}}}}_{\Lambda \text {d}}^{(s)}\left( {{{\mathbf {{K}}}}_{\text {dd}}^{(s)}} \right) ^{-1}{{\mathbf {{r}}}}_{\text {d}}^{(s)}} \right) , \end{aligned}$$

considering that the matrices ${{\mathbf {{K}}}}_{\text {dd}}^{(s)}$ can be inverted, i.e. they do not exhibit singularities arising from the existence of rigid body modes (RBMs). The flexibility of the interface ${{\mathbf {{F}}}}_{\text {I}}$ can be understood as the condensation of the domain stiffness matrices at the interface whereas $\Delta {{\mathbf {{g}}}}_{\text {I}}$ is, in turn, the condensed residual force vector at the interface $\Gamma _{\text {I}}$.

After solving the flexibility problem in (147) and obtaining the Lagrange multiplier field $\Delta \pmb {\Lambda }$, the domain displacement increments $\Delta {{\mathbf {{d}}}}^{(s)}$ can be independently calculated for each domain as

150 $$\begin{aligned} \Delta {{\mathbf {{d}}}}^{(s)}=\left( {{{\mathbf {{K}}}}_{\text {dd}}^{(s)}} \right) ^{-1}\left( {{{\mathbf {{r}}}}_{\text {d}}^{(s)}-{{\mathbf {{K}}}}_{\text {d}\Lambda }^{(s)}\Delta \pmb {\Lambda }} \right) . \end{aligned}$$

A blend of direct solvers are employed to independently compute the factorizations of the domain stiffness and an iterative solver is utilized for the solution of the interface problem in (147) which is never assembled in practice and, thus, requiring a considerably low memory profile [13, 14]. Since the resolution of the interface problem and the computation of the domain solution fields are inherently parallel tasks, the methodology scales well in massively parallel computers.

If one or more subdomains $\Omega ^{(s)}$ exhibit rigid body modes (RBMs), the corresponding matrices ${{\mathbf {{K}}}}_{\text {dd}}^{(s)}$ are not invertible and the expressions in (147) to (149) need to be modified considering the generalized inverse for each domain ${{{\mathbf {{K}}}}_{\text {dd}}^{(s)}}^{+}$. The new expressions basically extend the unknown field ${{\mathbf {{d}}}}$ accounting for the rigid body mode intensities ${\pmb {\alpha }}$ as explained in [8, 20, 22]. This methodology is general for the parallel processing of any dual system (83) or (142) but its implementation in commercial FE packages is regarded highly intrusive.

In this view, exploiting the novel features of the DIM method, i.e. a continuous fictitious discretization of the interface, a new methodology to handle rigid body modes has been proposed in [8] and adopted in this contribution. The method is described in detail in [8] and essentially adds a new term to the variational statements in (45) to (46) or  (112) to (114) which penalizes a function of the type $\frac{1}{2}\left| \left| \bar{{\mathbf {{g}}}}\right| \right| ^2$. It is important to mention that the new term is nullified in the solution since it is related with the Euler-Lagrange equations of the constraint variational principle, this giving rise to the term “consistent penalty”. The new variational statements in (45) to (46) and (112) to (114) read:

151 $$\begin{aligned}&\delta \tilde{\Pi }^{\text {u}}=\delta \Pi ^{\text {u}}+ \delta {\Pi }^{\text {u}}_{\text {RBM}}=\delta \Pi ^{\text {u}}+\sum _{r=1}^{N_{\text {r}}}c_{\text {u}}\int _{\Gamma _{\text {D}}^{(r)}}\delta {\bar{{\mathbf {{g}}}}_{\text {u}}}\cdot {\bar{{\mathbf {{g}}}}_{\text {u}}}\;d\Gamma =0, \end{aligned}$$

152 $$\begin{aligned}&\delta \tilde{\Pi }^{\text {p}}=\delta \Pi ^{\text {p}}+ \delta {\Pi }^{\text {p}}_{\text {RBM}}=\delta \Pi ^{\text {p}}+\sum _{r=1}^{N_{\text {r}}}c_{\text {p}}\int _{\Gamma _{\text {D}}^{(r)}}\delta {\bar{{g}}_{\text {p}}}\cdot {\bar{{g}}_{\text {p}}}\;d\Gamma =0, \end{aligned}$$

153 $$\begin{aligned}&\delta \tilde{\Pi }^{\theta }=\delta \Pi ^{\theta }+ \delta {\Pi }^{\theta }_{\text {RBM}}=\delta \Pi ^{\theta }+\sum _{r=1}^{N_{\text {r}}}c_{\theta }\int _{\Gamma _{\text {D}}^{(r)}}\delta {\bar{{g}}_{\theta }}\cdot {\bar{{g}}_{\theta }}\;d\Gamma =0, \end{aligned}$$

where $\Gamma _{\text {D}}^{(r)}$ corresponds to the interface segments such that $\Gamma _{\text {D}}^{(r)}=\Omega ^{(s)}\cap D^{(r)}$, $N_{\text {r}}$ representing the number of patches utilized to handle the RBMs (cf. Fig. 8) and $c_{\square }$ denotes the penalty coefficient utilized to enforce the new condition. A more detailed formulation considering the weak and discretized contributions of the stabilizing interface patches to the global system can be found in [8] for the irreducible formulation.

### Remark 5.1

Note that the term $c\dot{\theta }$ in (137) allows for the computation of $K^{-1}_{\theta \theta }$ since it behaves similarly than a mass matrix in a dynamic setting. However, for the choice of null specific heat $c=0$ the RBMs need to be eliminated with the addition of stabilizing interface contributions as indicated in (153) leading to a modified matrix of the type

154 $$\begin{aligned} \tilde{{\mathbf {{K}}}}_{\theta \theta }={{\mathbf {{K}}}}_{\theta \theta }+c_{\theta }\tilde{{\mathbf {{R}}}}^{\text {T}}_{\theta }{{\mathbf {{R}}}}_{\theta }, \end{aligned}$$

$c_{\theta }>0$ corresponding to the penalization term.

The linearized set of equations in (83) and (142) obtained with a Newton-like scheme is solved iteratively for each load/time step $\Delta t$ (cf. Newton-Krylov-Schur methods [9, 15]). In this view, two types of iterations can be identified. A first type refer to the solution of the non-linear problem with successive linear approximations and second type arise from the solution of the flexibility problem in (147) where usually Conjugate Gradient or GMRES iterates are considered. The Schur complements are utilized for the local solutions at each domain $\Omega ^{(s)}$ as indicated in (150).
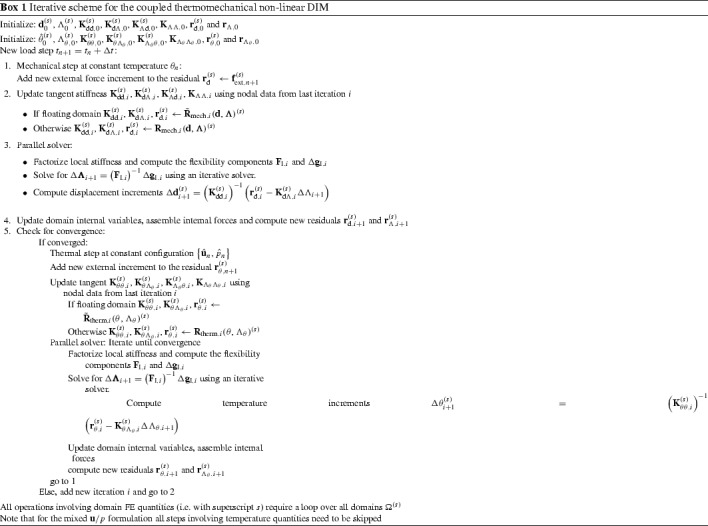


Assuming a fix domain decomposition and a given Delaunay interface discretization, the iterative scheme for the coupled thermomechanical non-linear DIM framework is summarized in Box 1.

## Framework validation through representative simulations

Two representative tests are selected to assess the performance of the multifield domain decomposition framework. Attention is focused on the continuity of the hybrid solution field at the interface, the convergence rate upon mesh refinement and the influence of the stabilization parameters as well as the activation of the Lagrange multipliers for the different solution fields at the interface. Finite strain theory and plane strain conditions are considered in all two dimensional examples.

### Mixed ${\mathbf {{u}}}/p$ formulation for incompressible problems

**Fig. 9:**
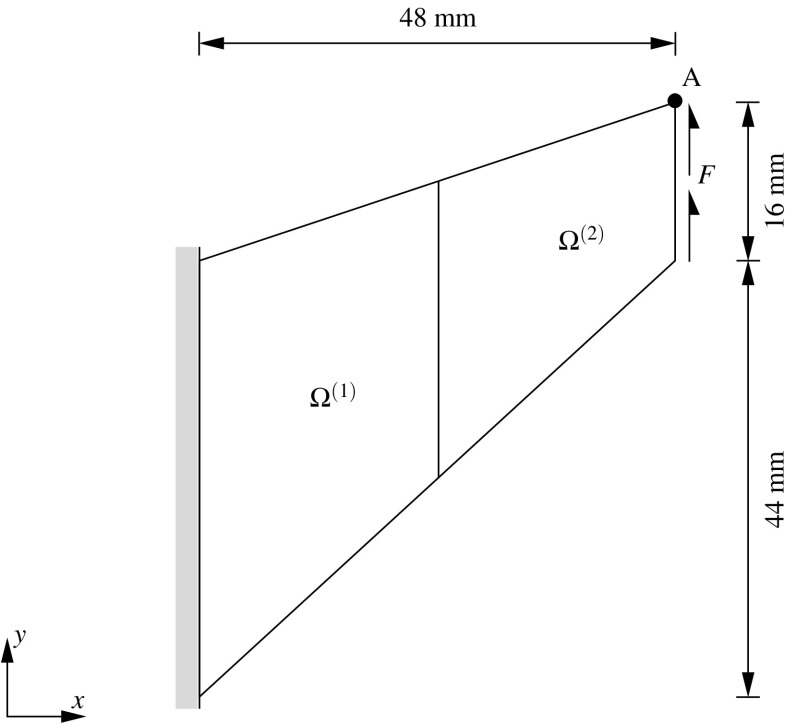
Geometry, boundary conditions and domain decomposition of the Cook’s membrane test

The mixed ${{\mathbf {{u}}}}/p$ formulation is tested in the context of the DIM by solving the Cook’s membrane problem. This test constitutes a well known benchmark problem for assessing the performance of the mixed ${{\mathbf {{u}}}}/p$ element in both compressible and incompressible conditions (cf. [26, 37]). The setup is basically sketched in Fig. 9 where a vertical force *F* is applied at one end of the membrane while the opposite end is fixed in both directions.

The membrane is considered elastic with Young’s modulus $E=70$ N/mm$^2$ and the Poisson’s ratio $\nu$ is set to 1 / 3 and nearly 0.5 for the compressible and incompressible case, respectively.

**Fig. 10:**
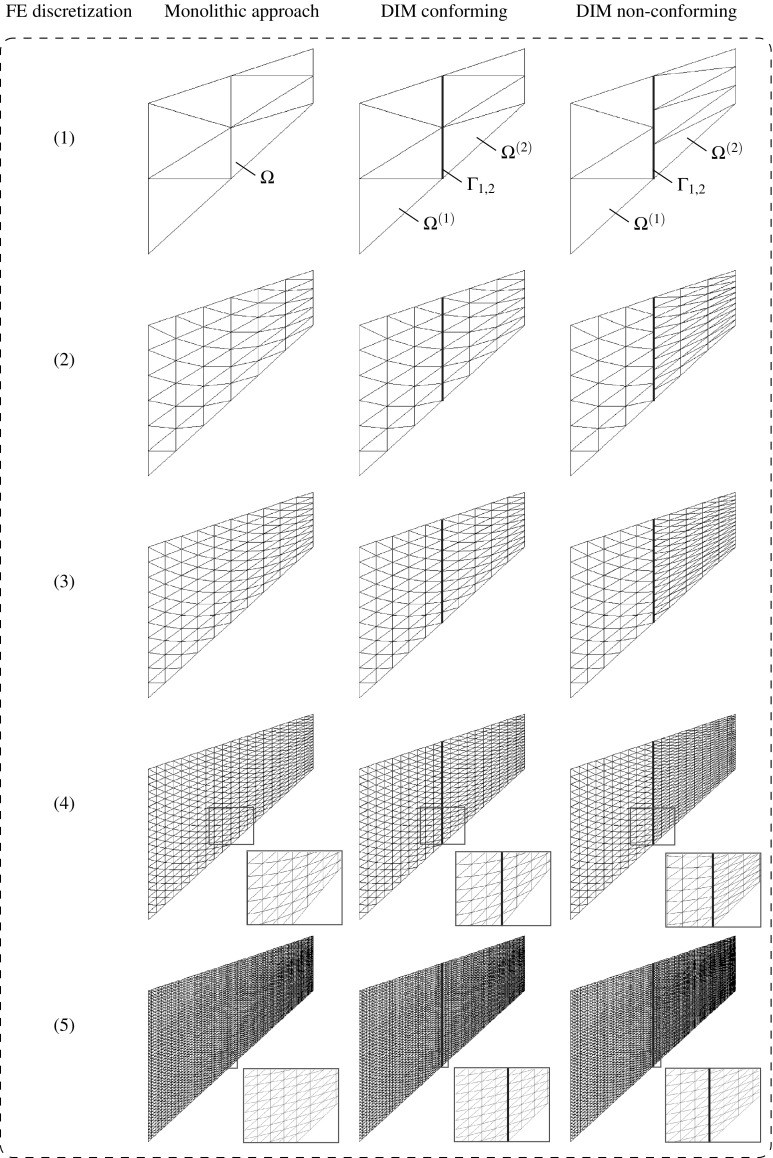
Finite element discretizations of the Cook’s membrane for the monolithic and DIM with conforming and non-conforming interfaces. The interface $\Gamma _{\text {I}}$ is depicted in blue and the *red rectangles* contain a zoom-in of the mesh around an interface region

In order to assess the convergence of the proposed methodology, five different discretization scenarios are considered. These concern a monolithic approach and the DIM on the body partitioned in two domains, i.e. $\Omega ^{(1)}$ and $\Omega ^{(2)}$ depicted in Fig. 9, with both conforming and non-conforming interfaces (cf. Table 1; Fig. 10).

**Table 1:** Number of finite elements for the five discretization scenarios considered at the Cook’s membrane test. Each discretization is particularized for the monolithic approach on the whole domain $\Omega$ and for the domain interface method (DIM) with conforming and non-conforming interfaces at partitions $\Omega ^{(1)}$ and $\Omega ^{(2)}$

Discretization	Monolithic approach	Domain interface method (DIM)			
		Conforming		Non-conforming	
		$\Omega ^{(1)}$	$\Omega ^{(2)}$	$\Omega ^{(1)}$	$\Omega ^{(2)}$
1	8	4	4	4	6
2	72	36	36	36	60
3	200	100	100	100	150
4	800	400	400	400	600
5	5000	2500	2500	2500	3000

**Fig. 11:**
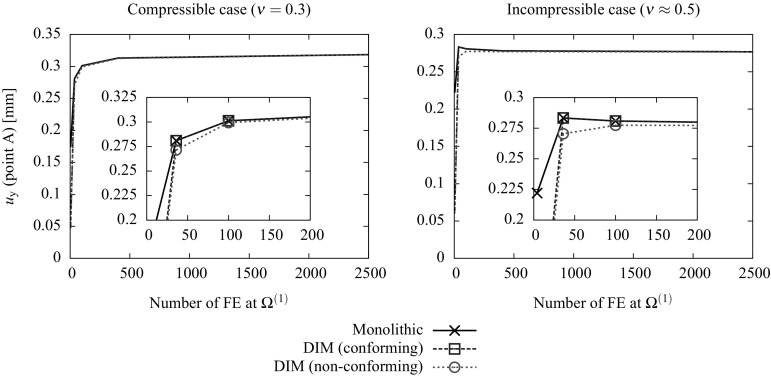
Convergence of the vertical displacement $u_{\text {y}}$ at point A upon different FE discretizations at the coarsest domain $\Omega ^{(1)}$ for the compressible (*left*) and incompressible (*right*) cases

The vertical displacement of point A (cf. Fig. 9) is monitored for both monolithic and domain decomposition analyses employing the five different FE discretizations. Figure 11 shows the convergence behaviour in terms of the vertical displacement for the compressible and incompressible cases upon different mesh refinements.

It is observed that all analyses provide an analogous convergent behaviour and the differences between the monolithic approach and the domain decomposition strategy are hardly visible beyond the third discretization, i.e. around 200–250 FE. In both compressible and incompressible analyses the DIM with non-conforming interfaces shows a slightly slower convergence with respect to the domain decomposition with conforming interfaces.

**Fig. 12:**
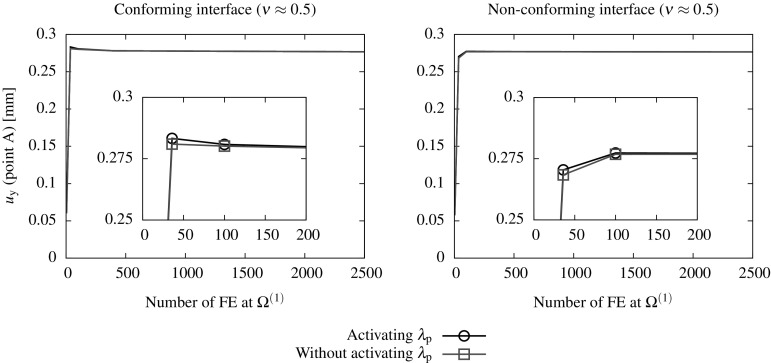
Influence of the activation of $\lambda _{\text {p}}$ on the convergence of the vertical displacement $u_{\text {y}}$ at point A upon different FE discretizations at the coarsest domain $\Omega ^{(1)}$. Results are plotted for the incompressible case employing both conforming (*left*) and non-conforming (*right*) interfaces

**Fig. 13:**
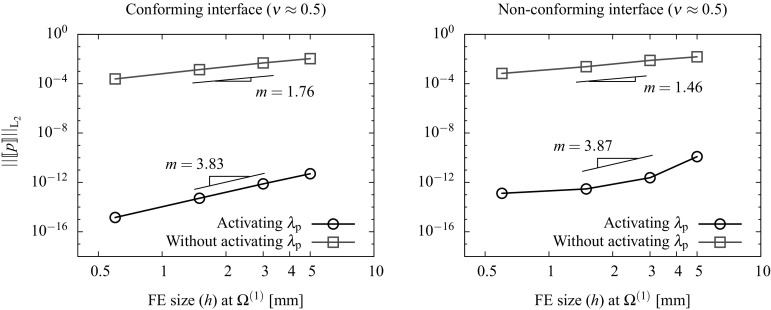
Influence of the activation of $\lambda _{\text {p}}$ on the convergence of the pressure transference across the interface upon different FE sizes *h* at the coarsest domain $\Omega ^{(1)}$. Results are plotted for the incompressible case employing both conforming (*left*) and non-conforming (*right*) interfaces

**Fig. 14:**
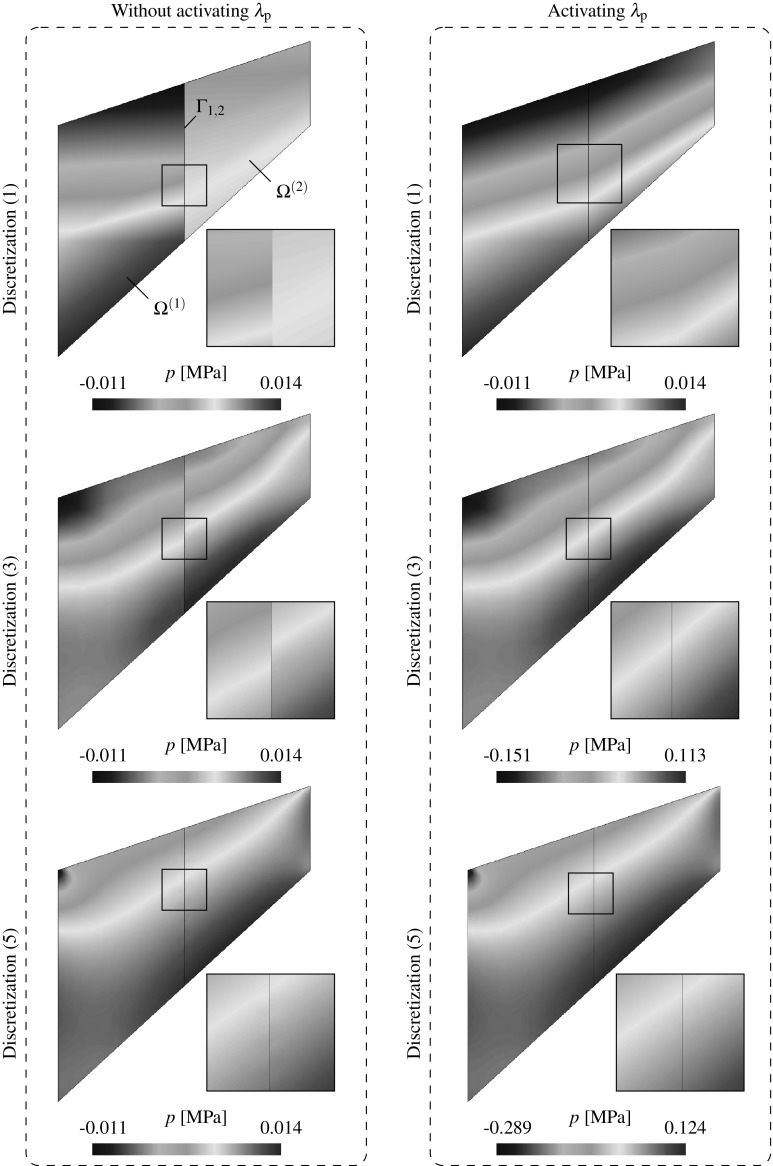
Influence of the activation of $\lambda _{\text {p}}$ on the pressure transference across the interface upon different FE discretizations. Results are plotted for the incompressible case employing non-conforming interfaces

**Fig. 15:**
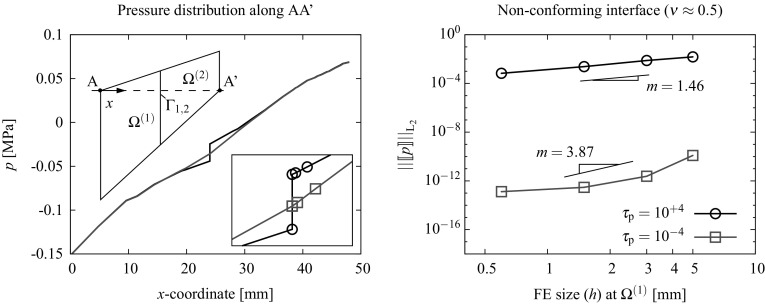
Sensitivity of the stabilization parameter $\tau _{\text {p}}$. *Left* Pressure distribution along the AA$^\prime$ segment across the non-conforming interface $\Gamma _{1,2}$ considering the FE discretization (3). *Right* convergence of the pressure transference across the interface upon different FE sizes *h* at the coarsest domain $\Omega ^{(1)}$. Results are plotted for the incompressible case employing non-conforming interfaces

The influence of the activation of the pressure Lagrange multiplier $\lambda _{\text {p}}$ is studied for the incompressible case employing both conforming and non-conforming interfaces (cf. Fig. 12).

It is observed that the activation of $\lambda _{\text {p}}$ hardly influences the displacement solution field and the monitored vertical displacement is almost identical in all cases. Consequently, the Lagrange multiplier $\lambda _{\text {p}}$ does not seem to play a relevant role in the correct transference of the displacements across the interfaces.

The influence of the activation of $\lambda _{\text {p}}$ on the correct transference of the pressure variable across the interface is studied by monitoring the norm of the pressure jump $\llbracket {p}\rrbracket$ at the interface $\Gamma _{1,2}$ between domains $\Omega ^{(1)}$ and $\Omega ^{(2)}$ as

155 $$\begin{aligned} \left| \left| \llbracket p\rrbracket \right| \right| _{\text {L}_2}=\displaystyle \left( {\int _{\Gamma _{1,2}}\left| p_2-P_{\Gamma _2}(p_1)\right| ^2 \; d\Gamma _{1,2}} \right) ^{1/2}, \end{aligned}$$

where $P_{\Gamma _2}(p_1)$ denotes the projection of the coarse domain interface pressure $p_1$ onto the fine interface discretization $\Gamma _2$. It can be observed that the error $\left| \left| \llbracket p\rrbracket \right| \right| _{\text {L}_2}$ reduces with decreasing element size *h* (cf. Fig. 13).

**Fig. 16:**
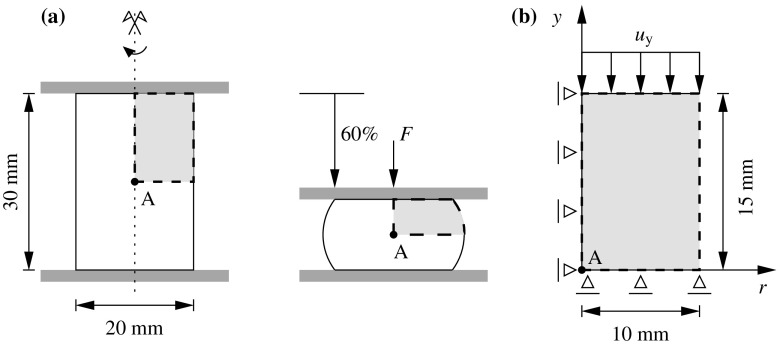
**a** Geometry and description of the problem ’Upsetting of a billet’. **b** Geometry and boundary conditions of the discretized quarter of the cylinder

**Fig. 17:**
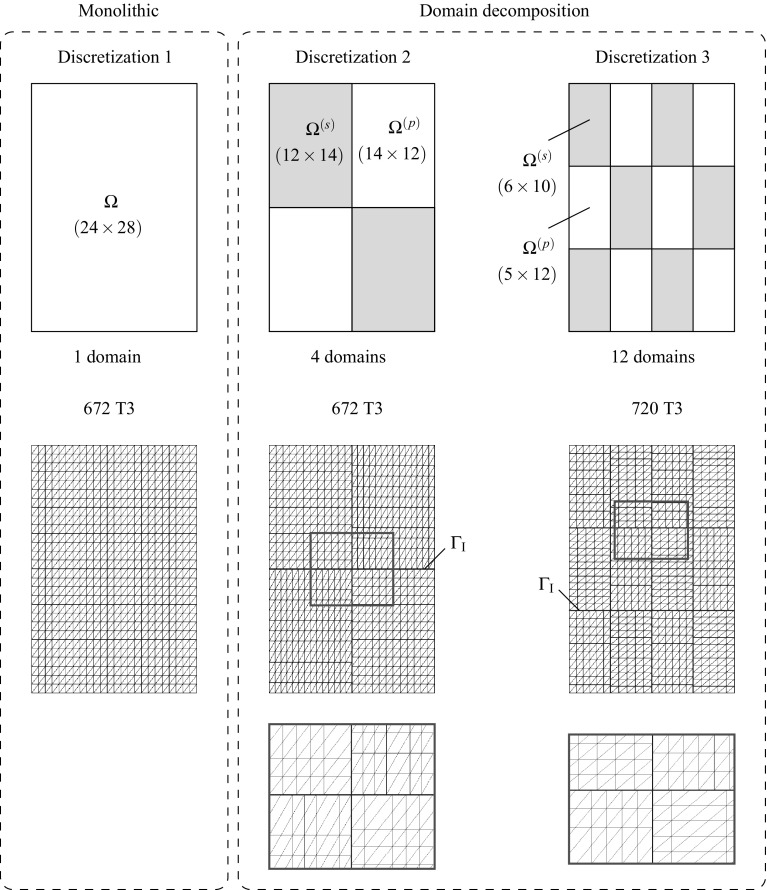
Finite element discretizations and domain decompositions for the bulk forming test. The *red rectangles* determine the close-up area

Moreover, the convergence rate is increased and the magnitude of the error is diminished when the pressure Lagrange multiplier $\lambda _{\text {p}}$ is activated. The benefits of the activation of $\lambda _{\text {p}}$ are illustrated in the pressure distribution plots across the interface $\Gamma _{1,2}$ for different mesh discretizations (cf. Fig. 14).

The deactivation of $\lambda _{\text {p}}$ leads to a pressure jump across the interface $\Gamma _{1,2}$. This effect is obviously less visible for the fine discretizations but the benefits of activating the pressure Lagrange multiplier are clear among all tested FE meshes.

The influence of the stabilization parameter $\tau _{\text {p}}$ is studied for the incompressible Cook membrane with a non-conforming interface. The pressure distribution along the segment AA$^\prime$ (cf. Fig. 15) is plotted for $\tau _{\text {p}}=10^{+4}$ and $\tau _{\text {p}}=10^{-4}$ employing the FE discretization (3) (cf. Table1; Fig. 10). Results depicted in Fig. 15 (left) clearly show that large values of $\tau _{\text {p}}$ lead to a pressure jump at the interface, located at $x=25$ mm, whilst low values of the stabilization parameter lead to a continuous pressure distribution along the intersection between AA$^\prime$ and $\Gamma _{1,2}$.

As shown in Fig. 15 (right), the error $\left| \left| \llbracket p\rrbracket \right| \right| _{\text {L}_2}$ diminishes upon mesh refinement for both values of the stabilization parameter. Moreover, for large values of $\tau _{\text {p}}$ the convergence rate decreases and the overall magnitude of the error increases. It is noted that a difference in eight orders of magnitude for $\tau _{\text {p}}$ leads to a pressure jump of around 0.02 Mpa.

### Coupled thermomechanical formulation applied to “bulk-forming” problems

In this example, a non-isothermal elastoplastic test is considered, in which a cylindrical metallic specimen is compressed until it experiences a 60% reduction of its initial height as depicted in Fig. 16a. The test is known as ’upsetting of a billet’ in the bulk metal forming field and is considered appropriate for the assessment of the correct transference of both temperature and pressure fields in the present framework. Adiabatic conditions are considered, i.e., no heat exchange is allowed between the body and its surrounding environment, and, therefore, the temperature increments are solely related to self heating induced by dissipation during large plastic deformation. Perfect stick is considered between the rigid tool and the specimen leading to ’barreling’ of the compressed billet.

As indicated in Fig. 16b, only a quarter of the specimen is considered due to the symmetry of the geometry and boundary conditions. In order to assess the correct transference of the pressure and temperature fields across the domain boundaries, the solution obtained with a monolithic approach, i.e. considering only one domain, is compared to the one obtained with four and twelve partitions with non-conforming interfaces as depicted in Fig. 17. A FE discretization of around 700 three-node axisymmetric linear elements is considered in all analyses.

The thermo-plastic model detailed in Appendix 1 is utilized with the material parameters summarized in Table 2.

The yield stress evolution is given by

156 $$\begin{aligned} \sigma _{\text {y}}(\alpha ,\theta )=\sigma _{0}(\theta )+h(\theta )\alpha \end{aligned}$$

with

157 $$\begin{aligned} \sigma _{0}(\theta )=\sigma _{0}(\theta _{0})\left( {1-w_{0}\left( {\theta -\theta _{0}} \right) } \right) , \end{aligned}$$

158 $$\begin{aligned} h_{0}(\theta )=h_{0}(\theta _{0})\left( {1-w_{\text {h}}\left( {\theta -\theta _{0}} \right) } \right) . \end{aligned}$$

The relations (156) to (158) describe a linear thermo-plastic softening widely used in metal plasticity for most steels in a temperature range from 300 to 400 K [36].

**Table 2:** Material parameters for the thermo-plastic model used in the bulk forming test

Bulk modulus	$\kappa$	166, 670	$\text {N}/\text {mm}^2$
Shear modulus	$\mu$	76, 920	$\text {N}/\text {mm}^2$
Flow stress	$\sigma _{0}$	700	$\text {N}/\text {mm}^2$
Hardening modulus	*h*	300	$\text {N}/\text {mm}^2$
Density	$\rho$	$7.8\times 10^{-9}$	$\text {N s}^2/\text {mm}^4$
Expansion coefficient	$\alpha$	$10^{-6}$	$\text {K}^{-1}$
Conductivity	*k*	45	$\text {N}/\text {s}\,\text {K}$
Capacity	*c*	$4.6\times 10^{8}$	$\text {mm}^2/\text {s}^2\,\text {K}$
Dissipation factor	$\chi$	0.9	
Flow stress softening	$w_{0}$	$3\times 10^{-4}$	$\text {K}^{-1}$
Hardening softening	$w_{\text {h}}$	$3\times 10^{-4}$	$\text {K}^{-1}$

**Fig. 18:**
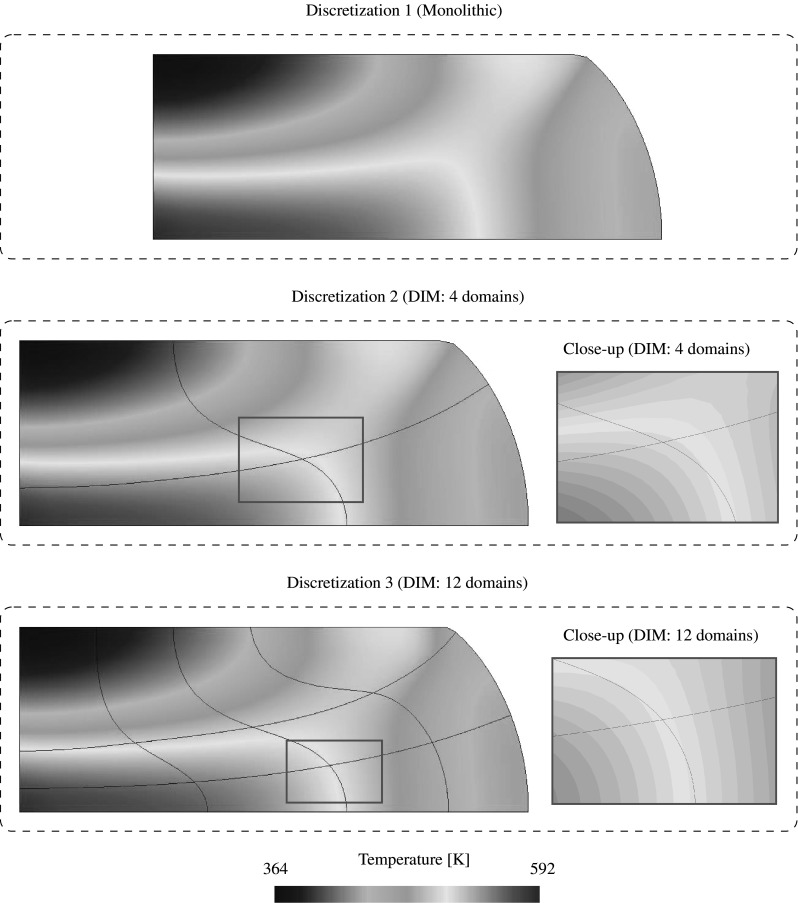
Temperature distribution at 60% height reduction for the monolithic and non-conforming domain decomposition analyses. No displacement magnification is applied at the deformed configurations

The temperature distribution for the three discretizations depicted in Fig. 17 are plotted in Fig. 18 at the final time step ($t=1\,\text {s}$). Note that the temperature fields of the domain decomposition analysis are remarkably comparable and equivalent to the one of the reference monolithic analysis. The close-ups at the non-conforming interface regions show a continuous temperature field which evidences its correct transference throughout the domains.

**Fig. 19:**
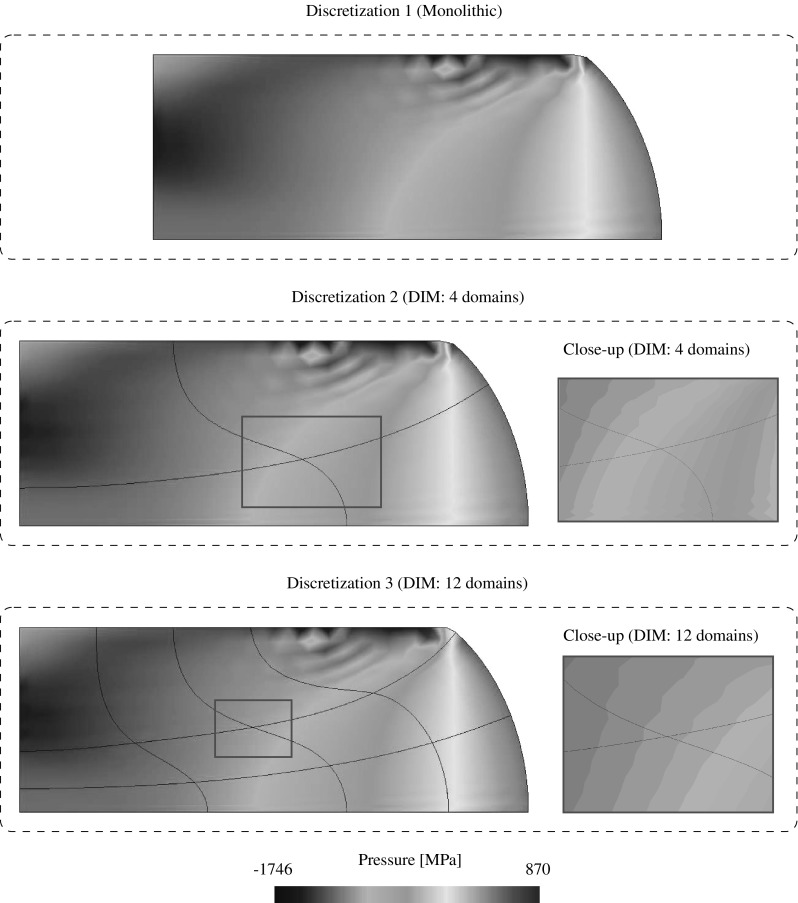
Pressure distribution at 60% height reduction for the monolithic and non-conforming domain decomposition analyses. No displacement magnification is applied at the deformed configurations

Figure 19 shows the pressure distribution for the three discretizations at the final time step ($t=1\,\text {s}$). As it was observed for the temperature field, the distribution of the pressure is continuous in all domain decomposition analyses across the non-conforming interfaces. It is observed that the pressure field at the top right corner of the specimen could be improved by using a finer discretization in both monolithic and domain decomposition approaches. However, the aim of the analyses is to proof that the results obtained with the domain decomposition framework are practically equivalent between them, and that no differences are observed when compared to the reference monolithic solution which is actually reflected in Fig. 19. Results shown in the close-ups in Figs. 18 and 19 evidence that the proposed domain decomposition framework provides a correct transference of the different fields in the mixed formulation.

**Fig. 20:**
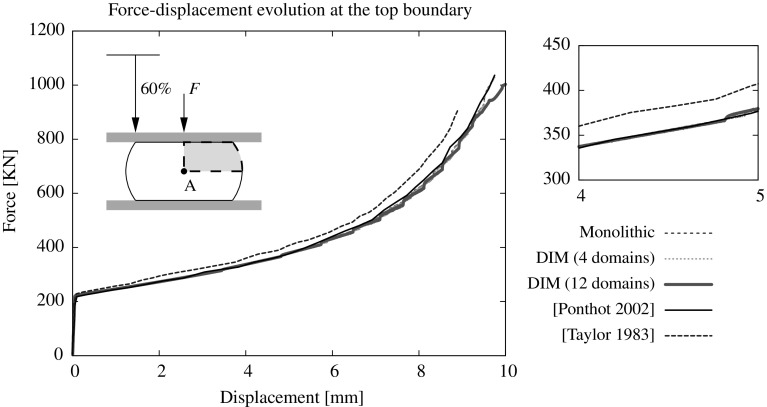
Evolution of the applied force against the displacement at the top part of the cylinder until 66.7% height reduction

The mechanical response of the billet, considering the applied force and displacement at the top of the specimen, is shown in Fig. 20. The reference responses reported by Ponthot [38], Taylor and Becker [29] are considered together with the monolithic and domain decomposition tests with four and twelve decompositions, respectively. The mechanical response of the domain decomposition method does not depend of the number of decompositions and conformity of the interfaces and is in agreement with the monolithic response which, in turn, shows that the selected constitutive model matches the results provided in literature, particularly the ones by Ponthot [29].

## Conclusions

The Domain Interface Method (DIM) presented in [8] is extended in this contribution for the case of mixed formulations encountered in multiphysics problems. Particularly, the domain decomposition framework is assessed in a large deformation setting for the particular case of mixed displacement/pressure formulations in incompressible materials and coupled thermomechanical problems with applications to bulk forming processes.

The extended DIM method for multiphysics problems essentially relies in an explicit discretization of the interface by means of a zero-thickness Delaunay triangulation as in the original methodology. This strategy allows setting a full tied contact in the most demanding scenarios, e.g. between geometrically incompatible interfaces. Continuity of the mixed solution field is satisfied across domains through the incorporation of Lagrange Multipliers. A Nitsche method is adopted in which a stabilization term is added at the constraint equations of the mixed field, i.e. one stabilization term per field. The incorporation of the stabilization term avoids the appearance of zero diagonal terms at the global system and instabilities are avoided if the LBB condition is not fulfilled by the chosen discretization. It is important to remark that, since the penalized term is part of the Euler-Lagrange equations of the variational principle, it will vanish upon mesh refinement (consistent penalty method).

The formulation presented in this manuscript is based on the identification of all Lagrange multipliers to connect the mixed field and their consideration in the weak variational form together with the corresponding consistent stabilization term. This technology is first assessed for a mixed displacement/pressure formulation to tackle incompressible materials in which a Polynomial Pressure Projection method is employed to account for the stability of the resulting systems within each domain. It is demonstrated that the pressure field is transferred with continuity across the non-conforming interfaces only if the Lagrange multiplier concerning the pressure field is taken into account, i.e. the continuity of the displacement field at the interface, enforced by the displacement Lagrange multipliers, does not guarantee the continuity of the pressure field at the interface. In fact, this effect vanishes upon mesh refinement since the stabilization term is no longer needed and vanishes too. It should be highlighted that the sensitivity of the results to the stabilization parameter is, however, very small. For the mixed displacement/pressure formulation, a variation of eight orders of magnitude in the pressure stabilization parameter would cause an insignificant jump in the pressure field.

The assessment of the DIM formulation for multiphysics problems is completed with a coupled thermomechanical problem with applications to bulk metal forming processes. In this case, a mixed displacement/pressure/temperature field is considered together with the corresponding Lagrange multipliers at the interface. Continuity of the three fields is obtained across all interfaces and the distribution of the mixed field is in very good agreement with the reference monolithic solutions. The mechanical response resulting from the upsetting of a billet studied in this example is remarkably close to those reported in literature which validates both the adopted constitutive model and domain decomposition technique.

An alternative non-intrusive methodology to handle RBMs was introduced in the original contribution presenting the DIM method. This methodology essentially adds an extra stabilization term to the energy functional with contributions of all adjacent interface patches avoiding, in this manner, the calculation of a pseudo-inverse at floating domains. As a result, the band structure of the global system is preserved and, for this reason, possible parallel solution strategies employed in other dual domain decomposition methods can still be employed. In the analyses considering the mixed displacement/pressure problem no singular modes may arise from the pressure field. However, in the thermomechanical problem, singular temperature modes with constant temperature distribution may arise at floating domains. An extra stabilization term analogous to the term restricting the RBMs should be considered but in the present formulation the subdomain matrices with diagonal temperature coefficients can still be inverted due to the introduction of a transitory term which acts in a similar way than the mass matrix in a dynamics problem.

The DIM method for mixed fields inherits all advantages outlined for the irreducible formulation, i.e. the interface connections are set up as a result of the connectivity in the automatic Delaunay triangulation and do not rely in user criteria for the master and slave DOFs or intermediate interface projections as it is done in established techniques such as the mortar approach. For this reason, the methodology shows potential to be used in cases where completely different discretizations are needed among domains since different physical phenomena are tacking place therein. The use of the DIM method could be specially advantageous when a particular domain discretization needs to be changed at a certain stage of the problem in order to improve the simulation of the physical process.

## Appendix 1: Mixed ${\mathbf{u}}/p$ formulation for incompressible problems

The standard irreducible formulation does not suffice for incompressible and nearly incompressible elastic materials, i.e. with Poisson’s ratio $\nu >0.4$. A discretization considering linear approximations using triangular elements can lead to highly oscillatory results [42]. Since the main problem in an irreducible formulation resides in the determination of the mean stress, i.e. the pressure *p*, it is convenient to separate it from the total stress field and treat it as an extra unknown in the constitutive equations. This is accomplished by decomposing the total stress into the deviatoric and spheric counterparts in the equilibrium equations and considering the constitutive equation relating the pressure and volumetric strain.

### Strong and weak forms of the mixed ${\mathbf{u}}/p$ formulation

A Neo-Hookean Hyperelastic constitutive model [7] is adopted in this study leading to the following strong form of the problem in which the body forces have been neglected:

159 $$\begin{aligned}&\text {FIND:}\left\{ \begin{aligned}&{{\mathbf {{u}}}}({{\mathbf {{x}}}}_{n}): \quad&\Omega _{n}\rightarrow \mathbb {R}^{2}, \\&p({{\mathbf {{x}}}}_{n}): \quad&\Omega _{n}\rightarrow \mathbb {R}, \end{aligned} \right. \\&\text {FULFILLING:} \nonumber \end{aligned}$$

160 $$\begin{aligned}&\text {Equilibrium equation:} \quad \bar{\pmb {\nabla }}\cdot {{\mathbf {{P}}}}={{\mathbf {{0}}}}, \quad \text {in } \Omega _{n} \end{aligned}$$

161 $$\begin{aligned}&\text {Constitutive pressure equation:} \quad \frac{p}{\kappa }=\frac{ln(J)}{J}, \quad \text {in } \Omega _{n} \end{aligned}$$

162 $$\begin{aligned}&\text {Dirichlet's boundary conditions:}\quad {\mathbf{u}}={\hat{\mathbf{u}}}, \quad \text {in } \Gamma _{u} \end{aligned}$$

163 $$\begin{aligned}&\text {Neumann's boundary conditions:}\quad {\mathbf{t}}={\hat{\mathbf{t}}}, \quad \text {in } \Gamma _{\sigma }, \end{aligned}$$

${\bar{\pmb {\nabla }}}$ being the material Nabla operator containing derivatives with respect to the previous configuration, ${{\mathbf {{P}}}}$ the first Piola-Kirchoff stress tensor, ${\hat{\mathbf{t}}}$ and ${\hat{\mathbf{u}}}$ the prescribed tractions and displacements at the corresponding boundaries $\Gamma _{\sigma }$ and $\Gamma _{u}$, respectively. The bulk modulus is denoted by $\kappa$ and $J=\left| \left| \mathbf{f}\right| \right|$ is the determinant of the deformation gradient tensor.

Equation (160) is solved at the current configuration, $\Omega _{n+1}$, considering the splitting of the Cauchy stress tensor into the spheric and deviatoric counterparts leading to

164 $$\begin{aligned} \bar{\pmb {\nabla }}\cdot {{\mathbf {{s}}}}+\bar{\pmb {\nabla }}{p}={{\mathbf {{0}}}}, \end{aligned}$$

with

165 $$\begin{aligned}&p=\frac{1}{3}tr({\pmb {\sigma }}), \end{aligned}$$

166 $$\begin{aligned}&{\mathbf {{s}}}=dev({\pmb {\sigma }}). \end{aligned}$$

The weak form of the problem stated in (160) to (163) is expressed through the corresponding variational principle. The solution ${\pmb {\mathcal {V}}}_{\square }$ and weighting $\hat{\pmb {\mathcal {V}}}_{\square }$ spaces for the displacements and pressure fields read:

167 $$\begin{aligned}&{\pmb {\mathcal {V}}}_{\text {u}}:=\left\{ {{\mathbf {{u}}}}\Big /{{\mathbf {{u}}}}\Big |_{\Omega } \in {\mathcal {H}}^{1}(\Omega ), \quad {\mathbf{u}}={\hat{\mathbf{u}}} \quad \text {in}\quad \Gamma _{\text {u}}\right\} , \end{aligned}$$

168 $$\begin{aligned}&\hat{\pmb {\mathcal {V}}}_{\text {u}}:=\left\{ \delta {{\mathbf {{u}}}}\Big / \delta {{\mathbf {{u}}}}\Big |_{\Omega }\in {\mathcal {H}}^{1}(\Omega ), \quad \delta {\mathbf{u}}={{\mathbf {{0}}}} \quad \text {in}\quad \Gamma _{\text {u}}\right\} , \end{aligned}$$

169 $$\begin{aligned}&{\pmb {\mathcal {V}}}_{\text {p}}:=\left\{ p\Big /p\Big |_{\Omega } \in {\mathcal {L}}^{2}(\Omega )\right\} , \end{aligned}$$

170 $$\begin{aligned}&\hat{\pmb {\mathcal {V}}}_{\text {p}}:=\left\{ \delta {p}\Big / \delta {p}\Big |_{\Omega }\in {\mathcal {L}}^{2}(\Omega )\right\} , \end{aligned}$$

${\mathcal {H}}^{1}(\Omega )$ and ${{{L}}}^{2}(\text {D})$ being the Sovolev space of functions with square integrable derivatives and the Lebesgue space of square integrable functions, respectively. The variational statement for the mixed ${{\mathbf {{u}}}}/p$ formulation reads:

171 $$\begin{aligned}&\text {FIND:}\left\{ \begin{aligned}&{{\mathbf {{u}}}}\in {\pmb {\mathcal {V}}}_{\text {u}}: \quad&\Omega _{n}\rightarrow \mathbb {R}^{2},\\&{p}\in {\pmb {\mathcal {V}}}_{\text {p}}: \quad&\Omega _{n}\rightarrow \mathbb {R},\\ \end{aligned} \right. \\&\text {FULFILLING:} \nonumber \end{aligned}$$

172 $$\begin{aligned}&\delta \Pi _{\text {int,ext}}^{\text {u}}({{\mathbf {{u}}}},\delta {{\mathbf {{u}}}})=-\int _{\Omega _{n}}{{\mathbf {{P}}}}: \bar{\pmb {\nabla }}(\delta {{\mathbf {{u}}}})\;d\Omega +\int _{\Gamma _{\sigma }}\hat{{\mathbf {{t}}}}\cdot \delta {{\mathbf {{u}}}}\;d\Gamma \nonumber \\&\quad =-\int _{\Omega _{n}}{J\left( {\underbrace{dev\left( {\pmb {\sigma }} \right) }_{{\mathbf {{s}}}}+p{{\mathbf {{1}}}}} \right) }: \bar{\pmb {\nabla }}(\delta {{\mathbf {{u}}}})\;d\Omega \nonumber \\&\quad +\int _{\Gamma _{\sigma }}\hat{{\mathbf {{t}}}} \cdot \delta {{\mathbf {{u}}}}\;d\Gamma =0, \quad \forall \delta {{\mathbf {{u}}}} \in \hat{\pmb {\mathcal {V}}}_{\text {u}}, \end{aligned}$$

173 $$\begin{aligned}&\text {AND}\nonumber \\&\delta \Pi ^{\text {p}}(p,\delta {p}){=}\int _{\Omega _{n}}\delta p\left( {\frac{ln(J)}{J}{-}\frac{1}{\kappa }p} \right) \;d\Omega {=}0, \quad \forall \delta p \in \hat{\pmb {\mathcal {V}}}_{\text {p}}, \end{aligned}$$

### Discretization using FE

A Galerkin-based spatial discretization is considered in which the solution field components and their variations are interpolated as:

174 $$\begin{aligned} {{\mathbf {{u}}}}({{\mathbf {{x}}}}_n)&=\sum _{a} {\mathbb {N}}_a^{\text {u}}({{\mathbf {{x}}}}_n){\hat{{\mathbf {{u}}}}}_{a}\quad \forall {{\mathbf {{x}}}}_n \in \Omega _{n},\end{aligned}$$

175 $$\begin{aligned} \delta {{\mathbf {{u}}}}({{\mathbf {{x}}}}_n)&=\sum _{a} {\mathbb {N}}_a^{\text {u}}({{\mathbf {{x}}}}_n)\delta {\hat{{\mathbf {{u}}}}}_{a}\quad \forall {{\mathbf {{x}}}}_n \in \Omega _{n},\end{aligned}$$

176 $$\begin{aligned} {p}({{\mathbf {{x}}}}_n)&=\sum _{a} {\mathbb {N}}_a^{\text {p}}({{\mathbf {{x}}}}_n){\hat{p}}_{a}\quad \forall {{\mathbf {{x}}}}_n \in \Omega _{n},\end{aligned}$$

177 $$\begin{aligned} \delta {p}({{\mathbf {{x}}}}_n)&=\sum _{a} {\mathbb {N}}_a^{\text {p}}({{\mathbf {{x}}}}_n)\delta {\hat{p}}_{a}\quad \forall {{\mathbf {{x}}}}_n \in \Omega _{n}, \end{aligned}$$

where the subscript *a* denotes the discrete nodes corresponding to the displacement interpolation and ${\mathbb {N}}^{\text {mec}}$ refer to the shape functions utilized for the displacement and pressure fields.

The FE approximation of the variational statements in (172) and (173) considering the additional stabilization term in (183) can be written using the above interpolations (174) to (177) as follows:

178 $$\begin{aligned} \delta \Pi ^{\text {u}}({{\mathbf {{u}}}},\delta {{\mathbf {{u}}}})\approx \,&\delta \Pi ^{\text {u,h}}({\hat{{\mathbf {{u}}}}},\delta {\hat{{\mathbf {{u}}}}})= \delta \Pi ^{\text {u,h}}_{\text {int}}({\hat{{\mathbf {{u}}}}},\delta {\hat{{\mathbf {{u}}}}})+\delta \Pi ^{\text {u,h}}_{\text {ext}}({\hat{{\mathbf {{u}}}}},\delta {\hat{{\mathbf {{u}}}}}), \end{aligned}$$

179 $$\begin{aligned} \delta \Pi ^{\text {p}}({{p}},\delta {{p}})\approx \,&\delta \Pi ^{\text {p,h}}({\hat{p}},\delta {\hat{p}}), \end{aligned}$$

Considering that (178) and (179) hold for any virtual displacement $\delta {{\mathbf {{u}}}}$ and pressure $\delta {p}$, the residual vector of the variational principle accounting for the stabilization term is composed by

180 $$\begin{aligned} {{\mathbf {{R}}}}^{\text {u}}({\hat{{\mathbf {{u}}}}},{\hat{p}}) =&\int _{\Omega _{n}}\sum _{a} \left( {\left( {\hat{p}{{\mathbf {{1}}}}+dev[\pmb {\tau }]} \right) {{\mathbf {{F}}}}^{-\text {T}}{\bar{\nabla }{\mathbb {N}}^{\text {u}}_{a}}} \right) \;d\Omega \nonumber \\&\quad -\int _{\Gamma } \sum _{a}\left( {{{\mathbb {N}}^{\text {u}}_{a}}{\hat{t}}} \right) \;d\Gamma =0, \end{aligned}$$

181 $$\begin{aligned} {\text {R}}^{\text {p}}(\hat{p}) =&\int _{\Omega _{n}}\sum _{a} \left( {{\mathbb {N}}^{\text {p}}_{a}\frac{\ln (J)}{J}} \right) \;d\Omega \nonumber \\&\quad +\hat{p}\int _{\Omega _{n}}\frac{1}{\kappa }\sum _{a} \left( {{\mathbb {N}}^{\text {p}}_{a} {{\mathbb {N}}^{\text {p}}_{a}}^{\text {T}}} \right) \;d\Omega =0, \end{aligned}$$

The discrete equations (180) and (181) are linearized and solved incrementally via a Newton-Raphson procedure leading to the following system:

182 $$\begin{aligned} \left[ \begin{array}{c} {{\mathbf {{R}}}}^{\text {u}}\\ {\text {R}}^{\text {p}} \end{array} \right] + \left[ \begin{array}{cc} \dfrac{\partial {{\mathbf {{R}}}}^{\text {u}}}{\partial {\hat{{\mathbf {{u}}}}}} &{} \dfrac{\partial {{\mathbf {{R}}}}^{\text {u}}}{\partial {\hat{p}}}\\ \dfrac{\partial {\text {R}}^{\text {p}}}{\partial {\hat{{\mathbf {{u}}}}}} &{} \dfrac{\partial {\text {R}}^{\text {p}}}{\partial {\hat{p}}} \end{array} \right] \left[ \begin{array}{c} \Delta {\hat{{\mathbf {{u}}}}}\\ \Delta {\hat{p}} \end{array} \right] ={{\mathbf {{0}}}} \end{aligned}$$

### Stabilization of the pressure field

The mixed displacement/pressure (${\mathbf{u}}/p$) formulation presents oscillations for incompressible and nearly incompressible materials. Such oscillations arise when the same interpolation order utilized in both the displacements and pressure fields does not satisfy the LBB condition [2].

In the present study the Polynomial pressure projection (PPP) [6] is employed since, in contrast with other methods, it does not require a stabilization parameter depending on the mesh size or the calculation of higher-order derivatives.

The following projection term is added to the weak form of the constitutive equation involving the incompressibility condition (161)

183 $$\begin{aligned} \int _{\Omega _{n}}\frac{\beta }{\mu }\left( {p-\bar{p}} \right) \left( {\delta p-\delta \bar{p}} \right) \;d\Omega \end{aligned}$$

with

184 $$\begin{aligned}&\bar{p}=\frac{1}{3}\left( {p_1+p_2+p_3} \right) , \end{aligned}$$

185 $$\begin{aligned}&\delta \bar{p}=\frac{1}{3}\left( {\delta p_1+\delta p_2+\delta p_3} \right) , \end{aligned}$$

$\beta$ and $\mu$ being the stabilization parameter and shear modulus, respectively, and $p_1$ to $p_3$ denote the corresponding nodal pressure values of the mixed element. Application of the projection operator to the weak form of the constitutive equation involving the pressure term removes the instability arising when pressures and displacements are interpolated employing shape functions of the same order [6].

## Appendix 2: Coupled thermomechanical formulation (mixed ${\mathbf{u}}/p/\theta$)

In the following equations the strong and weak format of the coupled termomechanical problem for a single domain is outlined with the corresponding adopted constitutive relations and the staggered solution algorithm of the discretized FE quantities.

### Strong form of the coupled thermomechanical problem

The strong form of the coupled thermomechanical problem neglecting the body forces can be expressed as

186 $$\begin{aligned}&\text {FIND:}\left\{ \begin{aligned}&{{\mathbf {{u}}}}({{\mathbf {{x}}}}_{n}): \quad&\Omega _{n}\rightarrow \mathbb {R}^{2}, \\&p({{\mathbf {{x}}}}_{n}): \quad&\Omega _{n}\rightarrow \mathbb {R}, \\&\theta ({{\mathbf {{x}}}}_{n}): \quad&\Omega _{n}\rightarrow \mathbb {R}, \end{aligned} \right. \\&\text {FULFILLING:}\nonumber \end{aligned}$$

187 $$\begin{aligned}&\text {Equilibrium equation:} \quad \bar{\pmb {\nabla }}\cdot {{\mathbf {{P}}}}={{\mathbf {{0}}}}, \quad \text {in } \Omega _{n} \end{aligned}$$

188 $$\begin{aligned}&\text {Constitutive pressure equation:} \quad \frac{p}{\kappa }=\frac{\ln (J)}{J}, \quad \text {in } \Omega _{n} \end{aligned}$$

189 $$\begin{aligned}&\text {Energy balance:} \quad \rho _0\dot{U}+\bar{\pmb {\nabla }}\cdot {{\mathbf {{Q}}}}={D_{\text {int}}}+\rho _0R \end{aligned}$$

190 $$\begin{aligned}&\text {Dirichlet's boundary conditions:}\quad {\mathbf{u}}={\hat{\mathbf{u}}}, \quad \text {in } \Gamma _{u} \end{aligned}$$

191 $$\begin{aligned}&\text {Neumann's boundary conditions:}\quad {\mathbf{t}}={\hat{\mathbf{t}}}, \quad \text {in } \Gamma _{\sigma }, \end{aligned}$$

with $\rho _0$ being the initial density, ${\bar{\pmb {\nabla }}}$ being the material Nabla operator containing derivatives with respect to the original configuration, ${{\mathbf {{P}}}}$ being the first Piola-Kirchhoff stress tensor, $\dot{U}$ is the internal energy, ${{\mathbf {{Q}}}}$ denotes the nominal heat flux vector and *R* stands for the internal generated heat.

#### Remark 7.1

Since incompressibility is assumed in all constitutive relations, the splitting of the Cauchy stress tensor in the current configuration is considered together with the constitutive pressure equation (188) in the remaining of the formulation.

### Dissipation inequalities

The momentum and energy balance equations appearing in the strong form (187) to (191) are restricted by the the second law of thermodynamics from which the following dissipation inequalities can be written [35]:

192 $$\begin{aligned} D_{\text {int}}&={{\mathbf {{P}}}}:\dot{{\mathbf {{F}}}}+\theta \dot{\eta }-\dot{U}\ge 0, \end{aligned}$$

193 $$\begin{aligned} D_{\text {con}}&=\frac{1}{\theta }\pmb {\nabla }\theta \cdot {{\mathbf {{Q}}}}\le 0, \end{aligned}$$

where $D_{\text {int}}$ and $D_{\text {con}}$ denote the internal dissipation and dissipation due to conduction, respectively and ${\mathbf {{F}}}$ stands for the deformation gradient tensor. The entropy is denoted by $\eta$ and *U* and ${\mathbf {{Q}}}$ denote the internal energy and nominal heat flux, respectively. The expression in (192), also referred to as the Clausius Plank form of the second law of thermodynamics, indicates that the internal energy can never increase at the expense of decreasing stress power. The restriction of the dissipation by conduction in (193) indicates that the heat flux adopts the opposite sense of the temperature gradient which can be trivially verified by considering the expression of the heat flux according to Fourier’s law:

194 $$\begin{aligned} {{\mathbf {{Q}}}}=-k{\bar{\pmb {\nabla }}}\theta , \end{aligned}$$

with *k* representing the positive thermal conductivity coefficient.

### Thermomechanical model for $J_2$ flow theory

The following form of the free energy $\Psi$, i.e. the part of the internal energy *U* amenable to be transformed into work at constant temperature and defined as $\Psi =U-\theta \eta$, is considered for the adopted thermomechanical model at finite strains [35, 36]

195 $$\begin{aligned} \Psi =\underbrace{T(\theta )+M(\theta ,J)}_{\text {thermal}}+\underbrace{U(J)+W({\bar{{\mathbf {{b}}}}}^{\text {e}})}_{\text {hyperelastic}}+\underbrace{K({\bar{e}}^{\text {p}},\theta )}_{\text {hardening}}. \end{aligned}$$

The volume preserving part of the left Cauchy-Green tensor ${{\mathbf {{b}}}}^{\text {e}}$ is calculated as

196 $$\begin{aligned} {\bar{{\mathbf {{b}}}}}^{\text {e}}&=(J^{\text {e}})^{-\dfrac{2}{3}}{{\mathbf {{b}}}}^{\text {e}}, \end{aligned}$$

197 $$\begin{aligned} {{\mathbf {{b}}}}^{\text {e}}&={{\mathbf {{F}}}}({{\mathbf {{C}}}}^{\text {p}})^{-1}{{\mathbf {{F}}}}^{\text {T}}. \end{aligned}$$

The internal hardening variable is denoted ${\bar{e}}^{\text {p}}$ and $T(\theta )$ refers to the potential for the purely thermal entropy defined as

198 $$\begin{aligned} T(\theta )=c\left[ \theta -\theta _0-\theta \log \left( {\frac{\theta }{\theta _0}} \right) \right] , \end{aligned}$$

where *c* and $\theta _0$ correspond to the heat capacity and reference temperature, respectively. The function $M(\theta ,J)$ describes the thermomechanical coupling due to thermal expansion

199 $$\begin{aligned} M(\theta ,J)=\left( {\theta -\theta _0} \right) \left[ -3\alpha U'(J) \right] , \end{aligned}$$

$\alpha$ being the linear coefficient of thermal expansion. The explicit expression for $T(\theta )$ in (198) and $M(\theta ,J)$ in (199) are obtained under the assumption of constant specific heat capacity $c\ge 0$. The volumetric and deviatoric contributions of the elastic free energy are denoted as *U*(*J*) and $W({\bar{{\mathbf {{b}}}}}^{\text {e}})$, respectively and are defined as

200 $$\begin{aligned} U(J)&=\kappa \left[ \frac{1}{2}\left( {J^2-1} \right) -\ln (J) \right] ,\end{aligned}$$

201 $$\begin{aligned} W({\bar{{\mathbf {{b}}}}}^{\text {e}})&=\frac{1}{2}\mu \left[ \text {tr}({\bar{{\mathbf {{b}}}}}^{\text {e}})-3 \right] , \end{aligned}$$

with $\kappa >0$ and $\mu >0$ being the bulk and shear moduli, respectively.

The potential describing isotropic hardening $K({\bar{e}}^{\text {p}},\theta )$ presented in [39] and adopted in [36] is considered in this contribution as:

202 $$\begin{aligned}&K({\bar{e}}^{\text {p}},\theta )=\frac{1}{2}h(\theta )(\bar{e}^{\text {p}})^2-\left[ \sigma _0(\theta )-\sigma _{\infty }(\theta )\right] H(\bar{e}^{\text {p}}), \end{aligned}$$

203 $$\begin{aligned}&H(\bar{e}^{\text {p}})=\left\{ \begin{aligned} \bar{e}^{\text {p}}-\frac{1-\bar{e}^{\delta \bar{e}^{\text {p}}}}{\delta },&\quad \text {for}\quad \delta \ne 0 \\ 0, \quad \quad&\quad \text {for}\; \delta =0, \end{aligned} \right. , \end{aligned}$$

204 $$\begin{aligned}&\sigma _0(\theta )=\sigma _0(\theta _0)\left[ 1-\omega _0(\theta -\theta _0) \right] , \end{aligned}$$

205 $$\begin{aligned}&\sigma _\infty (\theta )=\sigma _\infty (\theta _0)\left[ 1-\omega _{\text {h}}(\theta -\theta _0) \right] , \end{aligned}$$

206 $$\begin{aligned}&h(\theta )=h(\theta _0)\left[ 1-\omega _{\text {h}}(\theta -\theta _0) \right] , \end{aligned}$$

where $\delta$ is the saturation exponent and $\sigma _0(\theta )$, $\sigma _\infty (\theta )$ and $h(\theta )$ control the linear thermal softening. The initial yield stress is represented by $\sigma _0(\theta _0)$, $\sigma _\infty (\theta _0)$ denotes the final saturation hardening stress and $h(\theta _0)$ represents the linear hardening modulus. The reference temperature $\theta _0$ is considered in expressions (204) to (206) and $\omega _0$ and $\omega _{\text {h}}$ denote the flow stress softening and hardening-softening parameters, respectively. The potential in (203) describing isotropic hardening is able to model materials exhibiting a combination of linear and saturation-type hardening. Other related models existing in literature can be found in the works of Johnson and Cook [19] and Bäker [3].

#### Remark 7.2

It is important to note that the spherical and deviatoric counterparts of the Kirchhoff stress tensor ${\pmb {\tau }}$ can be written through the energy functions $M(\theta ,J)$, *U*(*J*) and $W({\bar{{\mathbf {{b}}}}}^{\text {e}})$ [36] as

207 $$\begin{aligned}&{\pmb {\tau }}=pJ{\mathbf {{1}}}+dev\{{\pmb {\tau }}\}, \end{aligned}$$

208 $$\begin{aligned}&p=U'(J)+\partial _{\text {J}}M(J,\theta ), \end{aligned}$$

209 $$\begin{aligned}&dev\{{\pmb {\tau }}\}=2dev\{{\bar{{\mathbf {{b}}}}}^{\text {e}}\partial _{{\bar{{\mathbf {{b}}}}}^{\text {e}}} W({\bar{{\mathbf {{b}}}}}^{\text {e}})\}. \end{aligned}$$

The thermoelastic domain $\Phi$ can be expressed in terms of the true Kirchhoff stresses through the function $\phi$ as

210 $$\begin{aligned} \Phi :=\{({\pmb {\tau }},{\beta },\theta ):\phi ({\pmb {\tau }},{\beta },\theta )\le 0\}, \end{aligned}$$

where $\theta$ is the absolute temperature and ${\beta }$ is related to the internal variables as

211 $$\begin{aligned} \beta =-K'({\bar{e}}^{\text {p}},\theta ). \end{aligned}$$

In this study the Mises yield criterion, typically employed in metal plasticity, is adopted which can be formalized as

212 $$\begin{aligned} \phi ({\pmb {\tau }},{\beta },\theta )=\left| \left| dev\{ {\pmb {\tau }} \}\right| \right| + \sqrt{\frac{2}{3}}[\beta -\sigma _{\text {y}}(\theta )]\le 0, \end{aligned}$$

where $\sigma _{\text {y}}(\theta )>0$ denotes the flow stress. The evolution equation for the internal variables can be written as:

213 $$\begin{aligned}&L_{\text {v}}{{\mathbf {{b}}}}^{\text {e}} = -2 \lambda \partial _{\tau }\phi ({\pmb {\tau }},\beta ,\theta )\cdot {{\mathbf {{b}}}}^{\text {e}}, \end{aligned}$$

214 $$\begin{aligned}&\dot{\bar{e}}^{\text {p}} =\lambda \partial _{\beta }\phi ({\pmb {\tau }},\beta ,\theta ), \end{aligned}$$

215 $$\begin{aligned}&\dot{\eta }^{\text {p}} =\lambda \partial _{\theta }\phi ({\pmb {\tau }},\beta ,\theta ), \end{aligned}$$

where $L_{\text {v}}{{\mathbf {{b}}}}^{\text {e}}$ represents the Lie derivative of the left Cauchy-Green tensor ${{\mathbf {{b}}}}^{\text {e}}$ (cf. [36]) and $\lambda \ge 0$ is the consistency parameter satisfying the Kuhn-Tucker relations:

216 $$\begin{aligned} \lambda \ge 0, \quad \quad \phi ({\pmb {\tau }},\beta ,\theta )\le 0, \quad \quad \lambda \phi ({\pmb {\tau }},\beta ,\theta )=0. \end{aligned}$$

The evolution of the thermal response can be derived from the equivalent form of the energy balance equation in (189) as

217 $$\begin{aligned} \theta \dot{\eta }=-\pmb {\nabla }\cdot {{\mathbf {{Q}}}}+R+D_{\text {mec}}, \end{aligned}$$

$\eta$ being the entropy per unit of initial volume, *Q* is the nominal heat flux (cf. (194)), *R* denotes the internal generated heat and $D_{\text {mec}}$ refers to the mechanical dissipation satisfying

218 $$\begin{aligned} D_{\text {int}}:=D_{\text {mec}}+D_{\text {ther}}\ge 0, \end{aligned}$$

where $D_{\text {therm}}$ and $D_{\text {int}}$ are the thermal and internal dissipation, respectively. The temperature evolution equation can be obtained by manipulating the constitutive entropy equation [11] and reads

219 $$\begin{aligned} c\dot{\theta }=-{\bar{\pmb {\nabla }}}{{\mathbf {{Q}}}}+R+D_{\text {mech}}-H, \end{aligned}$$

*c* being the specific heat at constant strain and constant internal variables per unit of reference volume and *H* stands for the structural elastoplastic heat [36].

### Weak form of the coupled thermomechanical model

The weak form of the problem outlined in (187) to (191) is expressed through the corresponding variational principle. The solution ${\pmb {\mathcal {V}}}_{\square }$ and weighting $\hat{\pmb {\mathcal {V}}}_{\square }$ spaces for the displacements, pressure and temperature field read:

220 $$\begin{aligned}&{\pmb {\mathcal {V}}}_{\text {u}}:=\left\{ {{\mathbf {{u}}}}\Big /{{\mathbf {{u}}}}\Big |_{\Omega } \in {\mathcal {H}}^{1}(\Omega ), \quad {\mathbf{u}}={\hat{\mathbf{u}}} \quad \text {in}\quad \Gamma _{\text {u}}\right\} , \end{aligned}$$

221 $$\begin{aligned}&\hat{\pmb {\mathcal {V}}}_{\text {u}}:=\left\{ \delta {{\mathbf {{u}}}}\Big / \delta {{\mathbf {{u}}}}\Big |_{\Omega }\in {\mathcal {H}}^{1}(\Omega ), \quad \delta {\mathbf{u}}={{\mathbf {{0}}}} \quad \text {in}\quad \Gamma _{\text {u}}\right\} , \end{aligned}$$

222 $$\begin{aligned}&{\pmb {\mathcal {V}}}_{\text {p}}:=\left\{ p\Big /p\Big |_{\Omega } \in {\mathcal {L}}^{2}(\Omega ), \right\} , \end{aligned}$$

223 $$\begin{aligned}&\hat{\pmb {\mathcal {V}}}_{\text {p}}:=\left\{ \delta {p}\Big / \delta {p}\Big |_{\Omega }\in {\mathcal {L}}^{2}(\Omega )\right\} , \end{aligned}$$

224 $$\begin{aligned}&{\pmb {\mathcal {V}}}_{\theta }:=\left\{ {\theta }\Big /{\theta }\Big |_{\Omega } \in {\mathcal {L}}^{2}(\Omega ), \quad {\theta }={\hat{\theta }} \quad \text {in}\quad \Gamma _{\theta }\right\} , \end{aligned}$$

225 $$\begin{aligned}&\hat{\pmb {\mathcal {V}}}_{\theta }:=\left\{ \delta {\theta }\Big / \delta {\theta }\Big |_{\Omega }\in {\mathcal {L}}^{2}(\Omega ), \quad \delta {\theta }={0} \quad \text {in}\quad \Gamma _{\theta }\right\} , \end{aligned}$$

${\mathcal {H}}^{1}(\Omega )$ and ${{{L}}}^{2}(\text {D})$ being the Sovolev space of functions with square integrable derivatives and the Lebesgue space of square integrable functions, respectively. The variational statement for the coupled thermomechanical model reads:

226 $$\begin{aligned}&\text {FIND:}\left\{ \begin{aligned}&{{\mathbf {{u}}}}\in {\pmb {\mathcal {V}}}_{\text {u}}: \quad&\Omega _{n}\rightarrow \mathbb {R}^{2}, \\&{p}\in {\pmb {\mathcal {V}}}_{\text {p}}: \quad&\Omega _{n}\rightarrow \mathbb {R},\\&{\theta }\in {\pmb {\mathcal {V}}}_{\theta }: \quad&\Omega _{n}\rightarrow \mathbb {R},\\ \end{aligned} \right. \\&\text {FULFILLING:} \nonumber \end{aligned}$$

227 $$\begin{aligned}&\delta \Pi _{\text {int,ext}}^{\text {u}}({{\mathbf {{u}}}},\delta {{\mathbf {{u}}}})=-\int _{\Omega _{n}}{{\mathbf {{P}}}}: \bar{\pmb {\nabla }}(\delta {{\mathbf {{u}}}})\;d\Omega {+}\int _{\Gamma _{\sigma }}\hat{{\mathbf {{t}}}}\cdot \delta {{\mathbf {{u}}}}\;d\Gamma = 0, \nonumber \\&\qquad \qquad ~~~~~~~~~~~~~~~~~~~~ \forall \delta {{\mathbf {{u}}}} \in \hat{\pmb {\mathcal {V}}}_{\text {u}}, \end{aligned}$$

228 $$\begin{aligned}&\delta \Pi ^{\text {p}}(p,\delta {p})=\int _{\Omega _{n}}\delta p\left( {\frac{ln(J)}{J}{-}\frac{1}{\kappa }p} \right) \;d\Omega {=}0, \quad \forall \delta p \in \hat{\pmb {\mathcal {V}}}_{\text {p}}, \end{aligned}$$

229 $$\begin{aligned}&\text {AND}\nonumber \\&\delta \Pi _{\text {int,ext}}^{\theta }(\theta ,\delta \theta ){=}-\delta \Pi ^{\text {int}}_{\text {therm}}(\theta ,\delta \theta )+\delta \Pi ^{\text {ext}}_{\text {therm}}(\theta ,\delta \theta )=0,\nonumber \\&\qquad \qquad ~~~~~~~~~~~~~~~~~~~~ \forall \delta \theta \in \hat{\pmb {\mathcal {V}}}_{\theta }, \end{aligned}$$

230 $$\begin{aligned}&\delta \Pi ^{\text {int}}_{\text {therm}}(\theta ,\delta \theta )=\int _{\Omega _{n}}\left( {\delta \theta c \dot{\theta }} \right) \;d\Omega \nonumber \\&\quad ~~~~~~~~~~~~~~~~~~~~~~~~~~+\int _{\Omega _{n}}\left( {k\pmb {\nabla }\delta \theta \cdot \pmb {\nabla }\theta -\sqrt{\frac{2}{3}}\Delta \lambda \sigma _{\text {y}}(\theta )} \right) \;d\Omega , \end{aligned}$$

231 $$\begin{aligned}&\delta \Pi ^{\text {ext}}_{\text {therm}}(\theta ,\delta \theta )=\int _{\Omega _{n}}\left( {\delta \theta \hat{R}} \right) \;d\Omega +\int _{\Gamma _{n}}\left( {\delta \theta (-D)} \right) \;d\Omega , \end{aligned}$$

where $\hat{R}$ denotes the heat sources and *D* the heat flux acting at the surface $\Gamma _n$.

A Galerkin-based spatial discretization is considered in which the solution field components and their variations are interpolated as:

232 $$\begin{aligned} {{\mathbf {{u}}}}({{\mathbf {{x}}}}_n)&=\sum _{a} {\mathbb {N}}_a^{\text {u}}({{\mathbf {{x}}}}_n){\hat{{\mathbf {{u}}}}}_{a}\quad \forall {{\mathbf {{x}}}}_n \in \Omega _{n},\end{aligned}$$

233 $$\begin{aligned} \delta {{\mathbf {{u}}}}({{\mathbf {{x}}}}_n)&=\sum _{a} {\mathbb {N}}_a^{\text {u}}({{\mathbf {{x}}}}_n)\delta {\hat{{\mathbf {{u}}}}}_{a}\quad \forall {{\mathbf {{x}}}}_n \in \Omega _{n},\end{aligned}$$

234 $$\begin{aligned} {p}({{\mathbf {{x}}}}_n)&=\sum _{a} {\mathbb {N}}_a^{\text {p}}({{\mathbf {{x}}}}_n){\hat{p}}_{a}\quad \forall {{\mathbf {{x}}}}_n \in \Omega _{n},\end{aligned}$$

235 $$\begin{aligned} \delta {p}({{\mathbf {{x}}}}_n)&=\sum _{a} {\mathbb {N}}_a^{\text {p}}({{\mathbf {{x}}}}_n)\delta {\hat{p}}_{a}\quad \forall {{\mathbf {{x}}}}_n \in \Omega _{n},\end{aligned}$$

236 $$\begin{aligned} {\theta }({{\mathbf {{x}}}}_n)&=\sum _{a} {\mathbb {N}}_a^{\theta }({{\mathbf {{x}}}}_n){\hat{\theta }}_{a}\quad \forall {{\mathbf {{x}}}}_n \in \Omega _{n},\end{aligned}$$

237 $$\begin{aligned} \delta {\theta }({{\mathbf {{x}}}}_n)&=\sum _{a} {\mathbb {N}}_a^{\theta }({{\mathbf {{x}}}}_n)\delta {\hat{\theta }}_{a}\quad \forall {{\mathbf {{x}}}}_n \in \Omega _{n}, \end{aligned}$$

where the subscript *a* denotes the discrete nodes corresponding to the displacement interpolation. Functions ${\mathbb {N}}^{\text {u}}$, ${\mathbb {N}}^{\text {p}}$ and ${\mathbb {N}}^{\theta }$ refer to the shape functions utilized for the displacements, pressure and thermal fields.

The FE approximation of the virtual work expression in (227) to (229) can be written using the above interpolations (232) to (237) as follows:

238 $$\begin{aligned} \delta \Pi ^{\text {u}}({{\mathbf {{u}}}},\delta {{\mathbf {{u}}}})\,\approx \,&\delta \Pi ^{\text {u,h}}({\hat{{\mathbf {{u}}}}},\delta {\hat{{\mathbf {{u}}}}})= \delta \Pi ^{\text {u,h}}_{\text {int}}({\hat{{\mathbf {{u}}}}},\delta {\hat{{\mathbf {{u}}}}})+\delta \Pi ^{\text {u,h}}_{\text {ext}}({\hat{{\mathbf {{u}}}}},\delta {\hat{{\mathbf {{u}}}}}), \end{aligned}$$

239 $$\begin{aligned} \delta \Pi ^{\text {p}}({{p}},\delta {{p}})\,\approx \,&\delta \Pi ^{\text {p,h}}({\hat{p}},\delta {\hat{p}}), \end{aligned}$$

240 $$\begin{aligned} \delta \Pi ^{\theta }({\theta },\delta {\theta })\,\approx \,&\delta \Pi ^{\theta ,\text {h}}({\hat{\theta }},\delta {\hat{\theta }})= \delta \Pi ^{\theta ,\text {h}}_{\text {int}}({\hat{\theta }},\delta {\hat{\theta }})+\delta \Pi ^{\theta ,\text {h}}_{\text {ext}}({\hat{\theta }},\delta {\hat{\theta }}), \end{aligned}$$

Considering that (238) to  (240) hold for any virtual displacement $\delta {{\mathbf {{u}}}}$, pressure $\delta {p}$ and temperature $\delta {\theta }$, the residual vector of the variational principle is composed by

241 $$\begin{aligned} {{\mathbf {{R}}}}^{\text {u}}({\hat{{\mathbf {{u}}}}},{\hat{p}}) =&\int _{\Omega _{n}}\sum _{a} \left( {\left( {\hat{p}{{\mathbf {{1}}}}+dev[\pmb {\tau }]} \right) {{\mathbf {{F}}}}^{-\text {T}}{\bar{\nabla }{\mathbb {N}}^{\text {u}}_{a}}} \right) \;d\Omega \nonumber \\&\quad -\int _{\Gamma } \sum _{a}\left( {{{\mathbb {N}}^{\text {u}}_{a}}{\hat{t}}} \right) \;d\Gamma =0, \end{aligned}$$

242 $$\begin{aligned} {\text {R}}^{\text {p}}(\hat{p}) =&\int _{\Omega _{n}}\sum _{a} \left( {{\mathbb {N}}^{\text {p}}_{a}\frac{\ln (J)}{J}} \right) \;d\Omega \nonumber \\&\quad +\hat{p}\int _{\Omega _{n}}\frac{1}{\kappa }\sum _{a} \left( {{\mathbb {N}}^{\text {p}}_{a} {{\mathbb {N}}^{\text {p}}_{a}}} \right) \;d\Omega =0, \end{aligned}$$

243 $$\begin{aligned} {\text {R}}^{\theta }(\hat{\theta }) =&\int _{\Omega _{n}}\sum _{a}\left[ {\mathbb {N}}^{\theta }_{a} c \dot{\hat{\theta }}\right. \nonumber \\&\quad \left. + \left( {\bar{\nabla }{\mathbb {N}}^{\theta }_{a}} \right) ^{\text {T}}\left( {k{\bar{\nabla }}\hat{\theta }} \right) - {\mathbb {N}}^{\theta }_{a}\left( {\sqrt{\frac{2}{3}}\sigma _{\text {y}}\hat{\theta }\Delta \lambda } \right) \right] \;d\Omega \nonumber \\&\quad -\int _{\Omega _{n}}\sum _{a} \left( {{\mathbb {N}}^{\theta }_{a} \hat{R}} \right) \;d\Omega - \int _{\Gamma _{n}}\sum _{a} \left( {{\mathbb {N}}^{\theta }_{a} {D}} \right) \;d\Gamma =0, \end{aligned}$$

### Coupled thermomechanical algorithm

The coupled discrete equations (241)–(243) are linearized and solved incrementally via a Newton-Raphson procedure. In fact, a staggered strategy is employed in which the mechanical problem is solved at constant temperature and the thermal problem is solved keeping a constant configuration. The algorithm in Box 2 summarizes the staggered resolution strategy on the linearized set of equations.

## Footnotes

1 In the sequel, all summations of integrals over the interface patches $D_{n}^{(q)}$, i.e. with *q* from 1 to the total number of interface patches $N_{\text {q}}$, are denoted as integrals over $D_{n}$ skipping the summation over *q* in order to simplify the notation.

2 Note that the the subscripts utilized in equations (148) and (149) refer directly to the subscripts in (83) but the thermal system in (142) can be treated in the same way by considering $\theta$ instead of *d* and $\Lambda _{\theta }$ instead of $\Lambda$.
